# Supplementary material for: Addressing alcohol-related harms in the local night-time economy: a qualitative process evaluation from a complex systems perspective
Source: BMJ Open. 2022 Aug 25;12(8):e050913. doi: 10.1136/bmjopen-2021-050913 (PMC9422880; doi:10.1136/bmjopen-2021-050913)
Supplement: Supplementary data [file bmjopen-2021-050913supp001.pdf]

Supplementary Material 1: Reporting Checklist

Standards for Reporting Qualitative Research (SRQR)*

http://www.equator-network.org/reporting-guidelines/srqr/

Page no(s).

Title and abstract

**Title** - Concise description of the nature and topic of the study Identifying the study as qualitative or indicating the approach (e.g., ethnography, grounded theory) or data collection methods (e.g., interview, focus group) is recommended	First page
**Abstract** - Summary of key elements of the study using the abstract format of the intended publication; typically includes background, purpose, methods, results, and conclusions	First page

Introduction

**Problem formulation** - Description and significance of the problem/phenomenon studied; review of relevant theory and empirical work; problem statement	Introduction, paraphs 1-2
**Purpose or research question** - Purpose of the study and specific objectives or questions	Introduction, final paragraph

Methods

**Qualitative approach and research paradigm** - Qualitative approach (e.g., ethnography, grounded theory, case study, phenomenology, narrative research) and guiding theory if appropriate; identifying the research paradigm (e.g., postpositivist, constructivist/ interpretivist) is also recommended; rationale**	Methods, paragraph 1
**Researcher characteristics and reflexivity** - Researchers' characteristics that may influence the research, including personal attributes, qualifications/experience, relationship with participants, assumptions, and/or presuppositions; potential or actual interaction between researchers' characteristics and the research questions, approach, methods, results, and/or transferability	Methods, paragraph 6
**Context** - Setting/site and salient contextual factors; rationale**	Introduction, paragraph 1; Methods paragraph 2
**Sampling strategy** - How and why research participants, documents, or events were selected; criteria for deciding when no further sampling was necessary (e.g., sampling saturation); rationale**	Methods, paragraphs 3-10; Tables 3 and 4
**Ethical issues pertaining to human subjects** - Documentation of approval by an appropriate ethics review board and participant consent, or explanation for lack thereof; other confidentiality and data security issues	Methods, paragraph 11

**Data collection methods** - Types of data collected; details of data collection procedures including (as appropriate) start and stop dates of data collection and analysis, iterative process, triangulation of sources/methods, and modification of procedures in response to evolving study findings; rationale**	Methods, paragraphs 3-10
**Data collection instruments and technologies** - Description of instruments (e.g., interview guides, questionnaires) and devices (e.g., audio recorders) used for data collection; if/how the instrument(s) changed over the course of the study	Methods, paragraphs 6-10; Supplementary material 2
**Units of study** - Number and relevant characteristics of participants, documents, or events included in the study; level of participation (could be reported in results)	Tables 3 and 4
**Data processing** - Methods for processing data prior to and during analysis, including transcription, data entry, data management and security, verification of data integrity, data coding, and anonymization/de-identification of excerpts	Methods, paragraphs 13-15
**Data analysis** - Process by which inferences, themes, etc., were identified and developed, including the researchers involved in data analysis; usually references a specific paradigm or approach; rationale**	Methods, paragraphs 13-15; Supplementary Material 3
**Techniques to enhance trustworthiness** - Techniques to enhance trustworthiness and credibility of data analysis (e.g., member checking, audit trail, triangulation); rationale**	Methods, paragraphs 14-15; Supplementary Material 3

Results/findings

**Synthesis and interpretation** - Main findings (e.g., interpretations, inferences, and themes); might include development of a theory or model, or integration with prior research or theory	Results
**Links to empirical data** - Evidence (e.g., quotes, field notes, text excerpts, photographs) to substantiate analytic findings	Results

Discussion

**Integration with prior work, implications, transferability, and contribution(s) to the field -** Short summary of main findings; explanation of how findings and conclusions connect to, support, elaborate on, or challenge conclusions of earlier scholarship; discussion of scope of application/generalizability; identification of unique contribution(s) to scholarship in a discipline or field	Discussion, paragraphs 1-10
**Limitations** - Trustworthiness and limitations of findings	Discussion, paragraphs 11-14

Other

**Conflicts of interest** - Potential sources of influence or perceived influence on study conduct and conclusions; how these were managed	Conflict of interest statement

**Funding** - Sources of funding and other support; role of funders in data collection, interpretation, and reporting	Funding statement

*The authors created the SRQR by searching the literature to identify guidelines, reporting standards, and critical appraisal criteria for qualitative research; reviewing the reference lists of retrieved sources; and contacting experts to gain feedback. The SRQR aims to improve the transparency of all aspects of qualitative research by providing clear standards for reporting qualitative research.

**The rationale should briefly discuss the justification for choosing that theory, approach, method, or technique rather than other options available, the assumptions and limitations implicit in those choices, and how those choices influence study conclusions and transferability. As appropriate, the rationale for several items might be discussed together.

Reference:

O'Brien BC, Harris IB, Beckman TJ, Reed DA, Cook DA. **Standards for reporting qualitative research: a synthesis of recommendations.** *Academic Medicine*, Vol. 89, No. 9 / Sept 2014 DOI: 10.1097/ACM.0000000000000388

Supplementary Material 2: Data collection instruments

Interview topic guide: service providers (community safety and police)

1. Introduction:

- Study introduction, confidentiality, audio recording
- Any questions?

2 Can you tell me a bit about [*organisation/team*]? How do you operate?
Probe around: interaction with other parts of the organisation; interaction with other services; intelligence generation

3. As an [position], what does your role entail?
Probe around: typical shift; generating taskings; usual activities

4. Can you tell me a bit more about the people you tend to work with or encounter on patrols?
Probe around: drinkers in particular – drinking habits, purchasing habits, location of drinking, behaviours associated with drinking; co-occurrence of drinking and other behaviours

5. From your perspective, what are the major alcohol-related challenges in [LA]?
Probe around: specific population groups; different types of venues; specific venues; specific areas of LA; specific drinks

- **How do you think [LA] compares to other areas?**

5. Can you tell me about the Late Night Levy?

- **How did the levy come about in [LA]?**
    - *Probe around:* supporters/opponents, who drove the implementation, etc.
- **What is the aim of the levy?**
    - *Probe around:* targeting of intervention (drinkers, retailers)

6. How has the levy changed the services you provide?
Probe around: working with other services, deployment of resources; focus of shifts/taskings

7. Thinking about the people you encounter and the venues you engage with, do you think the initiative has had any impacts on them?
For drinkers, probe around: consumption and behaviour (intoxication to ASB/crime)
For retailers: opening hours, engagement with police/council/community safety; management practices

8. Have you seen any other impacts of the initiative, either health or non-health related?
Probe around: why these have occurred

10. Over the past few years, have you been aware of, or involved in, any other new alcohol initiatives or services?
Probe around: interaction with the levy, impacts on retailers and drinkers

11. Wrap up and end

Interview topic guide: drinkers

1. To start off, can you tell me a bit about yourself?
Probe around: age, work, resident/visitor

2. What do you like about going out/drinking in [LA]?
Probe around: usual places they go and why; what they drink; who they are usually with; drinking at home vs. out

3. Have you noticed any changes in [LA] and the places to go out here?
Probe around: types of places, types of people drinking in them, reasons for changes

- **Are there any changes you want to see here to make [LA] a better place to go out?**

4. Have changed where you like to go out or where you buy drinks over the past few years?
Probe around: changes to drinking patterns – locations, amount, types of drink, time they drink, etc.

5. Do you think there are any problems with alcohol in [LA]?
Probe around: specific types of people, parts of the LA, specific venues, etc.

- **Have you ever been moved on whilst drinking on the streets or come into contact with police? Ever been ejected from a premise?**

6. [LA] has a Late Night Levy in place – have you heard about it?

- If yes, **what do you think the levy is trying to do?**
  - *Probe around:* aims, reason to bring it in, if they have seen any changes
- If not, explain levy: **what do you think about an initiative like that?**
  - *Probe around:* community safety/police presence, venue opening times, types of venues in area, prices, what changes in might bring in, etc

7. Have you noticed any changes in LA's night time environment?
Probe around: times places are open; drink offers, how venues are managed – e.g. door staff, tolerance for rowdiness/fighting/etc, police on street, community safety on street, etc

8. Wrap-up and end

Observation template: patrols

Fieldworker:
Patrol Officers:
Date:
Patrol time and length:

Pre-patrol briefing (if applicable):

- Specific aims of the patrol
- Areas to be patrolled
- Specific venues/locations to visit
- Previous incidents requiring follow-up action

How the patrol operates:

- Area of patrol
- Means of patrol (on foot, in car, etc.)
- How officers determine how to focus their attention (walk around and spot incidents, respond to calls, etc.)

Incidents encountered: for each incident with drinking involved (or suspected):

- Location of incident
- Name of premise (if applicable)
- Individual or group encountered
- Characteristics of the individual/group (age, gender, dress, etc.)
- Behaviour of individual/group (drunk and disorderly, aggressive, subdued, unconscious, etc.)
- How patrol officers interact with individual/group encountered:
- Individual/group reaction to intervention
- Outcome of intervention
- Other services involved (police, ambulance premise staff, etc.)
- Patrol officers' interpretation of incident

Non-drinking incidents encountered:

- Types of incidents
- Types of individuals/groups involved

Patrol staff:

- Characteristics (age, gender, physical characteristics)
- Length of time working on this patrol
- Types of individuals/groups that attract their attention
- Types of individuals/groups that do not attract their attention
- Impressions of problem drinking (types of people, areas of LA, types of premises, specific venues, specific drinks) and evolution over time
- Perceptions of LNL (aim, achieving aim? how? changes in how the service has been delivered? Relationships with other services; public, venues)
- Knowledge and impressions of other alcohol interventions: Cumulative Impact Policies, Reducing the Strength – others; interaction with the levy

Researcher reflexivity

Supplementary Material 3: Coding framework

This supplementary material file presents our coding framework, which is described in tabular form and visually represented in the form of a map of the local system. The map is a synthesis of stakeholder perceptions and was used to generate hypotheses that the second phase of the evaluation examines in more detail. The map is comprised of a range of variables that emerged from the analysis of local and national data generated and collected in Phase 1 of the evaluation. To identify and define variables of interest, two researchers (EM and ME) independently generated and subsequently reconciled a list of variables that, from the perspective of system stakeholders, characterise the system of inquiry. A description of each variable is provided in the table, along with the other system variables that system stakeholders perceived each variable to be directly related to and/or from. The final column in the table provides an excerpt of data to illustrate each variable. The figure depicts these variables visually on a system map. Each variable is represented by a bubble; the lines represent relationships to other system variables and whether the two variables are positively (solid line) or inversely (dashed line) related to each other. The system represented in the diagram is bounded so that it contains the elements operating within the geographical area of the LA. However, the local system is a complex system and therefore these boundaries should be considered 'open'.

Table 1: System variables and relationships within the local system

System	Variable	Description	Connected to/from (positive or inverse relationship)	Example(s) (source)
National	National support for NTE regulation	The degree to which national policymakers support the introduction and extension of regulation in the NTE.	To: LA powers to regulate NTE and alcohol licensing (+); societal alcohol-related economic/health harms (+)<br><br>From: alcohol-industry lobbying / discourse-shaping activity (-)	"The Government intends to rebalance the licensing regime to enable local 'Licensing Authorities' (LAs) and the police to clamp down on alcohol-related crime and disorder, particularly late at night; to allow wider considerations and the views of local communities to be taken into account in licensing decisions; to protect children from the harm of alcohol; and introduce a late night levy to help pay for other costs caused by late-night drinking." (Impact assessment for Police Reform and Social Responsibility Bill 2010)
	LA powers to regulate NTE and alcohol licensing	The specific powers available to LAs to regulate the NTE and control the provision of alcohol through alcohol licensing powers, such as the LNLs, CIPs, and EMROs.	To: LA support for the levy (+); alcohol outlet density (-)<br><br>From: national support for NTE regulation (+)	"The late night levy is a discretionary power enabling licensing authorities in England and Wales to raise a contribution towards policing the late-night economy from holders of premises licences or club premises certificates." (House of Commons, Late Night Levy Briefing, 2015)
	Societal alcohol-related economic and health harms	The societal harms associated with alcohol, including the associated healthcare costs, productivity losses and population-level health harms.	To: national support for NTE regulation (+)<br><br>From: alcohol consumption (+)	"Alcohol misuse also costs the United Kingdom economy an estimated £7.3 billion a year in lost productivity and the National Health Service in England an estimated £3.5 billion a year. In England, over 15,000 people die from alcohol-related illnesses each year." (Next steps on delivering the Government's Alcohol Strategy, 2013)
	Alcohol industry lobbying / discourse-shaping activity	Activities the alcohol industry engages in to lobby for and frame debates in terms favourable to their corporate interests.	To: industry profitability (+) ; national support for NTE regulation (-)<br><br>From: support for PPP schemes (+)	"We believe that the Council should maintain its current voluntary best practice approach which is delivering real results and crucially is focused on eliminating the source of the problems rather than simply paying for any clean up.[…] This is in line with the National Alcohol Strategy which states that targeted action taken voluntarily by pubs and clubs themselves is most effective in curbing irresponsible drinking and associated drunken violence. The Home Secretary, Theresa May, in publishing the strategy suggested that a legislative approach, either national or local, was a 'sledgehammer' which all too often misses its target and that a partnership approach was more effective." (Consultation response, trade organisation representing on-licence premises).
National	Industry profitability	The financial gains realised by the alcohol industry.	To: business rates/licence fees (+)<br><br>From: alcohol-industry lobbying / discourse-	"[The LNL] will impose a significant further cost burden on the hospitality industry in the [local] area when the overall costs the industry must pay whether food, drink, labour and taxes contribute to rise and customers' real incomes shrink impacting on profitability". (Consultation response, operator of managed pubs).

**System**	**Variable**	**Description**	**Connected to/from** (positive or inverse relationship)	**Example(s) (source)**
			shaping activity (+); customer numbers (+)	
Local	Alcohol outlet density	Concentration of premises selling alcohol for consumption on or off the premises.	To: alcohol consumption (+)<br><br>From: LA powers to regulate the NTE and alcohol licensing (-); mixed land use (+)	"The number of licenced premises continues to grow rapidly so that [LA] has one of the highest densities of pubs, bars, clubs and off licences in the country and second highest in London after the City of Westminster." (Licensing Policy 2013-2017)<br><br>"I: So from your perspective, what would you say are the major kind of alcohol related challenges in [LA]?<br>R: *There are too many licensed premises."* (Interview, Police licensing officer)
	Alcohol consumption	The amount of alcohol an individual consumes. Can be measured in the context of a single drinking event, or to describe patterns of consumption.	To: crime / ASB / disturbance (+); alcohol-related health harms (+)<br><br>From: alcohol outlet density (+) ; alcohol availability (+)	"Drinking above recommended maximum limits has become much more common over the past two decades, particularly among younger and middle aged men and women of all social groups. This trend is mirrored in [LA]. […] The effects of the increase in alcohol consumption seen over the past two decades are now being seen in the significant increases in alcohol-related attendances and admissions in the NHS." (Licensing Policy 2011-2014)<br><br>"Alcohol consumption is increasingly identified as a major factor behind violent crime and disorder in the borough with serious consequences to victims, businesses and local communities." (Licensing Policy 2013-2017)
	Alcohol-related health harms	Individual health harms experienced as a result of alcohol consumption.	To: LA support for the levy (+); emergency service usage (+)<br><br>From: alcohol consumption (+); crime / ASB / disturbance (+); police and community safety presence (-)	"In reviewing our Licensing Policy we have been mindful that [LA's] residents suffer from high levels of alcohol-related ill health and early deaths." (Licensing Policy 2013-2017)<br><br>"Studies assessing the effectiveness of limiting the density of alcohol outlets showed greater alcohol outlet density to be associated with increased alcohol consumption and harms including injury, violence, crime and medical harm. Small numbers of concentrated problematic nightlife venues often cause a large proportion of alcohol-related harm, violence and injuries in city centres." (Public Health Report 2012)
Local	Mixed land use	The degree to which areas within the LA are mixed residential and commercial.	To: residential reputation (-); alcohol outlet density (+)<br><br>From: population change (+)	"[LA name] is a densely occupied area, with no clear delineation between residential and commercial premises and the Licensing Authority will need to carefully balance the conflicting needs of residents, patrons and businesses in relation to the introduction of flexible opening hours for the sale and supply of alcohol and late night refreshments." (Licensing Policy 2011-2014)

**System**	**Variable**	**Description**	**Connected to/from** (positive or inverse relationship)	**Example(s) (source)**
	Population change	The demographic characteristics of the local population and its rate of change.	To: mixed land use (+)<br><br>From: residential reputation (+)	"[LA], however, is undergoing a process of rapid change and this is likely to continue. The number of people living here has grown from 178,000 in 2001 to an estimated 199,130 in 2010. One of the reasons for this is the increase in the number of young adults who are moving into inner London, and starting families. […] This has turned many parts of the borough, which were previously exclusively commercial, into mixed-use hubs incorporating commercial and residential premises in very close proximity." (Licensing Policy 2011-2014)
	Residential reputation	The extent to which the local area is viewed as a desirable place to live.	To: population change (+); council tax (+)<br><br>From: perceptions of safety (+); mixed land use (-); nightlife reputation (+/-); strength of the local economy (+)	"I spoke to the public health lead for alcohol in [neighbouring LA] last week and he was saying this, exactly. […] Having all those bars, having all those people drinking, that's what they call regeneration, whereas in [this LA], my impression what they call regeneration is a good place to live, not a good place to party." (Interview, Public Health specialist)<br><br>"[LA name's] nightlife is marvellous and one of the reasons I love being a resident here." (Consultation response, resident)
	Nightlife reputation	The extent to which a local area is viewed as a desirable place to go out.	To: residential reputation (+/-); customer numbers (+)<br><br>From: perceptions of safety (+); diversity of NTE offer(+)	[Participant] almost always goes out in [LA] when he goes out. He likes the variety of places and that you can get fairly cheap drinks and still be in Zone 1. (Excerpt from fieldnotes)<br><br>"[LA] has a reputation for its vibrant nightlife and the council, along with its partners the Metropolitan Police and London Fire Brigade recognise that many people are working hard to ensure that [LA]'s clubs, pubs and bars provide a safe and attractive venue for customers." (Licensing Policy 2011-2014)
	Cost of policing and managing the NTE	The resources required to manage and regulate the NTE; includes police, community safety, street cleaning, licensing and trading standards.	To: LA support for the levy (+)<br><br>From: emergency service usage (+); crime / ASB / disturbance (+)	"[LA] has a well established night-time economy that has continued to grow since the introduction of the Licensing Act in 2005. The number of late-night and 24 hours premises is high and they are spread across the borough. The costs of policing the late night economy are substantial." (Consultation response, Executive Member for Community Safety).

System	Variable	Description	Connected to/from (positive or inverse relationship)	Example(s) (source)
Local	LA support for the levy	The degree to which key stakeholders responsible for local area, support the need to introduce the levy; includes elected members and responsible authorities.	To: Late Night Levy<br><br>From: LA powers to regulate NTE and alcohol licensing (+); cost of policing and managing the NTE (+); alcohol-related health harms (+); strength of the local economy (-); litter / vomit / urine / graffiti (+); support for PPP schemes (-)	"The supporting statement for the consultation at Appendix A sets out why the Council, supported by the police, believes the levy is necessary and this is still felt to be strong and compelling." (Consultation response, Executive Member for Community Safety).
	Perceptions of safety	The extent to which individuals feel safe in their local environment; includes both residents and visitors perceptions.	To: residential reputation (+); nightlife reputation (+)<br><br>From: police and community safety presence (+)	"[LA] is a safe place to socialise, this is my perception and common among my going out friends" (Consultation response)<br><br>"I've lived in a lot of places – [LA] and, in particular, [popular NTE area] are hardly a war zone!" (Consultation response)
	Emergency service use	The use of emergency services, including ambulances, A&E and police.	To: cost of policing and managing the NTE (+)<br><br>From: police and community safety presence (-); alcohol-related health harms (+)	"Generally, as density of licensed premises in LA increases so does the number of ambulance callouts (shown on map) and also levels of alcohol-related crime." (Public Health Report, 2012)
	Crime / ASB/ Disturbance	The prevalence of crime, anti-social behaviour, disturbance and nuisance that is fuelled by alcohol consumption.	To: alcohol-related health harms (+); cost of policing and managing the NTE (+)<br><br>From: alcohol consumption (+); police and community safety presence (-); numbers of intoxicated individuals on the street (+)	"A comparison of alcohol related violence prior to deregulating licensing hours in 2004 with 2011 shows that in 2004 alcohol related crime peaked between the hours of 11pm and midnight. By 2011 the peak hours for alcohol related crime had expanded and shifted to midnight to 5am with a corresponding 600% increase in alcohol related crime." (Licensing Policy 2013-2017)<br><br>"I'm very pleased to see [name] council taking this initiative. I live in [area] which has more than enough pubs and clubs and where noisy customers in the street are a problem in the small hours." (Consultation response, resident)
Local	Police and community safety presence	The visible presence of police and community safety officers on	To: emergency service usage (-); alcohol-related	"From October 2012, a new late night levy will empower local areas to make those businesses that sell alcohol late into the night contribute towards the

System	Variable	Description	Connected to/from (positive or inverse relationship)	Example(s) (source)
		the streets and in and around licensed premises.	health harms (-); crime / ASB / disturbance (-); perceptions of safety (+); litter / vomit / urine / graffiti (-)<br><br>From: resources for managing / policing the NTE (+)	cost of policing and wider local authority action. This will help enable visible and proactive policing at targeted locations where there are local needs." (The Government's Alcohol Strategy, 2012)<br>"The proposals we have for spending the levy are to: • have a uniformed presence patrolling at weekends to deal with enforcement issues, drunkenness, street urination, rowdy and nuisance behaviour and assistant to vulnerable individuals identified. • Provide additional policing to support operations targeting crime and disorder. […]" (LNL Consultation, 2013)
	Resources for managing / policing the NTE	The funds, staff and equipment needed to police and manage the NTE.	To: police and community safety presence (+) ; street cleaning services (+)<br><br>From: Late Night Levy; business rates / licence fees (+); council tax (+); failure of PPP schemes (-)	"The fees set for licensed premises have not increased since their introduction in 2005. During the intervening eight years, the demands for services have increased along with the number of premises opening late and it is now necessary to look to alternative ways of financing the services needed to manage the impacts." (LNL Consultation, 2013)<br>"[The Late Night Levy] could potentially curb some of the more dangerous behaviour and sales of alcohol, as well as provide the Council and Police with additional resources in recognition of the extra costs involved in policing, monitoring and cleaning the areas around premises with late alcohol licences." (Consultation response, political party)
	Business rates / license fees	The fees that premises pay to the LA; the business rate is based on the rateable value of the premise and the license fee is applicable for all premises with a license to sell alcohol for on- or off-premise consumption.	To: resources for managing / policing the NTE (+)<br><br>From: premise closures (-); industry profitability (+)	"Like I said, I don't want boarded up buildings. I can't have a whole area of [LA]. Because I live here, it's not good for the economy, it's not good for crime and disorder, you know. It's, it's not good, you know, it's not good for the Council, you know, we don't get business rates if premises are empty." (Interview, Licensing officer)<br>"The council and police should already be budgeting for such a problem. Bars and pubs already pay a licence fee to the council – what's that currently paying for?" (Consultation response, licence holder)
	Council tax	Tax paid by households to LA based on the value of the property to fund LA services.	To: resources for managing / policing the NTE (+)<br><br>From: residential reputation (+)	"Business rates/council tax is already astronomically high so I don't see why this shouldn't already be covered?" (Consultation response)
Local	Support for PPP schemes	The degree to which alcohol retailers and the alcohol industry support voluntary partnerships with the police and local authority, as well as other	To: failure of PPP schemes (+); alcohol-industry lobbying / discourse-shaping activity (+); LA support for the	"It penalises the hard work that many operators have undertaken in partnerships with the local authority and the police." (Consultation response, licence holder)<br>"[LA] has engaged in active partnership working with its licensed premises so as to ensure high standards of management that will prevent nuisance

System	Variable	Description	Connected to/from (positive or inverse relationship)	Example(s) (source)
		businesses, including the BIDs, PubWatch, Best Bar None, etc.	levy (-)<br><br>From: Late Night Levy	and crime. It has participated in Central Government initiatives, held training events and developed a number of best practice schemes, including the Community Alcohol Partnership programme […] There are six Pubwatches operating throughout the borough though this still sees only a small percentage of businesses actively engaging with the Council and police. Despite this engagement and the standards achieved within premises, [LA] continues to have a high level of alcohol related crime." (Consultation response, Executive Member for Community Safety).
	Failure of PPP schemes	The number of PPP schemes that licensees choose to no longer support.	To: street cleaning services (-); resources for managing / policing the NTE (-); customer numbers (-)<br><br>From: support for PPP schemes (-)	"It is also unrealistic in the current economic climate to expect operators to have funds available to support involvement in voluntary initiatives AND pay the late night levy. Where no discount is available to encourage participation in such schemes and to provide some financial relief for doing so, such schemes will wither on the vine as membership falls away." (Consultation response, Pub company)
	Opening hours	The hours until which a premise is licensed to sell alcohol.	To: alcohol availability (+); clustering of closing times (-); Late Night Levy<br><br>From: Late Night Levy	"[…] we understand that a large number of our members' business will choose to voluntarily restrict their hours. Generally, it is only dedicated late night businesses which will generate sufficient revenues after midnight to justify retaining licensing hours within the levy period. Many traditional pubs or restaurants will have later closing times but will not regularly use them and, as the levy is based on permission not use, will therefore voluntary relinquish them rather than face an additional cost." (Consultation response, trade association representing on-licence trade)
	Clustering of closing times	The degree to which premises close at the same time.	To: numbers of intoxicated individual on streets (+); diversity of NTE offer (-)<br><br>From: opening hours (-)	"One consequence of the levy is likely to be that a significant number of premises will reduce their hours to 12 midnight resulting in a return to a non staggered closing time culture, contrary to government policy." (Consultation response, supermarket chain).
Local	Number of intoxicated individuals on the street	The number of people out on the street who have been drinking.	To: crime / ASB / disturbance (+); litter / vomit / urine / graffiti (+)<br><br>From: clustering of closing times (+)	"The Council should also consider the risk of a defacto uniform terminal hour for premises arising as operators cut back their premises licences to cease at whenever the levy starts to bite. We have highlighted the actual experience of [LA with a levy] above. This will mean more customers on the street at the same time with resultant pressure on resources such as taxis, fast food outlets and policing." (Consultation response, Pub company)
	Alcohol availability	The ease and convenience of purchasing availability;	To: diversity of NTE offer (+); customer numbers	"Availability of and access to alcohol has an important influence on levels of alcohol consumption. Generally speaking, changes in the availability of

System	Variable	Description	Connected to/from (positive or inverse relationship)	Example(s) (source)
		availability is comprised of the density of licensed premises (physical availability), the hours in which alcohol is sold (temporal availability), and the cost of alcoholic beverages (economic availability).	(+); alcohol consumption (+)<br><br>From: opening hours (+); premise closures (-)	alcohol tend to be reflected sooner or later in changes in levels of alcohol consumption and alcohol-related harm." (Public Health Report, 2012)
	Premise closures	The number of alcohol-retailing premises who cease operating.	To: alcohol availability (-); business rates / licence fees (-); Late Night Levy<br><br>From: Late Night Levy	"There is a very real risk that [LA] Council will be responsible for premises closures, loss of jobs and income from rates (with higher outgoings in dealing with those people put out of work) from over-taxing those premises already struggling to cope with the burden they currently have." (Consultation response, Pub and bar operator)
	Street cleaning services	The services to keep streets clean, including clearing rubbish and recycling, washing away vomit and urine, and removing graffiti.	To: litter / vomit / urine / graffiti (-)<br><br>From: resources for managing / policing the NTE (+)	"The proposals we have for spending the levy are to: […] provide additional cleaning and service such as litter removal, graffiti removal and cleaning." (LNL Consultation, 2013)
	Litter/vomit/urine/graffiti	Quality of the physical environment; physical manifestations of incivility.	To: LA support for the levy (+)<br><br>From: street cleaning services (-); police and community safety presence (-); number of intoxicated individuals on the street (+)	"the Licensing Authority has seen an increase in concerns raised by local residents, Councillors and local businesses about the impact that the night time economy is having on the local environment in this area. Typical issues of concern include: • Public urination • Litter • Noise nuisance from patrons of licensed premises • Drug dealing • Thefts • Damage to property and vehicles • Obstruction of the public highway" (Licensing Policy, 2011-2014)<br><br>"For instance, a significant majority [of] litter on the streets at night comes from premises not licensed to tell alcohol at that time, such as takeaways and shops with late opening hours. It is illogical and unfair to make payment for cleaning up the sole responsibility of premises selling alcohol." (Consultation response, Pub company and brewer).
Local	Customer numbers	Number of patrons frequenting licensed premises.	To: footfall (+); industry profitability (+)<br><br>From: alcohol availability (+), nightlife reputation (+); failure of PPP schemes (-)	"Any reduction to its licensed hours made by venue operators will unquestionably have a detrimental impact on other local businesses in the area, including [LA's] many eateries, as there are likely to be fewer customers in the area for a shorter period of time, thus further affecting the financially lucrative late night economy." (Consultation response, licensee).
	Footfall	The number of individuals in the LA on a given night.	To: attractive area for businesses (+)	"We are bound to ask whether the Council has considered the consequences of closures not just to the licence holders in [LA] but the migration of customers into the neighbouring boroughs which will not only

System	Variable	Description	Connected to/from (positive or inverse relationship)	Example(s) (source)
			From: customer numbers (+)	affect the earlier evening trade but will also have the effect of damaging other businesses through lower footfall in that earlier evening." (Consultation response, Hospitality company)
	Attractive area for business	The degree to which an area is perceived as attractive for a business, includes number of residents and visitors, other types of establishments and 'friendliness' of Council to businesses.	To: local jobs (+); diversity of NTE offer (+)<br><br>From: footfall (+)	"The Council should consider it decision very carefully before [LA] becomes a place that becomes unattractive as a place to trade. [Pub company] will decide its future expansion and growth plans within London on the basis of how business friendly the incumbent Council is in any particular area." (Consultation response, pub company)
	Local jobs	The number of jobs in a local area, some of which are in the alcohol-retailing and hospitality sector.	To: strength of the local economy (+)<br><br>From: attractive area for businesses (+)	"This is an unfair tax on vulnerable businesses who provide wealth and jobs for the borough." (Consultation response, licence holder)<br><br>"But we're a poorer borough, we need people to have jobs, we don't want boarded up or empty premises". (Interview, Licensing officer)
	Strength of the local economy	The degree to which there are wealth generating activities in the LA. The NTE is one contributor to the local economy.	To: LA support for the levy (-); residential reputation (+)<br><br>From: local jobs (+)	"The council recognises that the entertainment and alcohol industry contributes to the borough by providing a variety of opportunities for entertainment as well as employment and career opportunities." (Licensing Policy 2011-2014)<br><br>"This is a significant additional cost for businesses to bear and it will affect profitability and viability for many. Some will choose to close earlier to avoid paying this, with knock on effects on turnover, GVA to the local economy and employment patterns." (Consultation response, trade association representing on-licence trade)
Local	Diversity of NTE offer	Different types of premises operating in the NTE, including: pubs, bars, clubs, restaurants, live music venues, off-licences, supermarkets, clubs, hotels and the characteristics of those premises (e.g. catering to different types of clientele).	To: nightlife reputation (+)<br><br>From: alcohol availability (+); clustering of closing times (-); attractive area for businesses (+)	"The council is keen to preserve a diverse mix of premises through the borough and wants to work with businesses, residents and partners through its Licensing Policy to resist the saturation of licensable premises of similar types within identified local areas." (Licensing Policy 2011-2014)<br><br>"We feel that there has not been enough research carried out into the benefits of a mixed and diverse night time economy in [LA} which could stagnate on the introduction of a levy. Premises where alcohol does not contribute significantly to profit but is offered after midnight may well close early. This will harm the diversity of offer, leaving only larger pubs and nightclubs willing to pay the levy." (Consultation response, pub company and brewer)

Abbreviations: A&E: Accident and Emergency; BID: Business Improvement District; CIP: Cumulative Impact Policy; EMRO: Early Morning Restriction Order; LA: Local Authority; LNL: Late Night Levy; NTE: night time economy; PPP: public-private partnership

Figure 1: system map

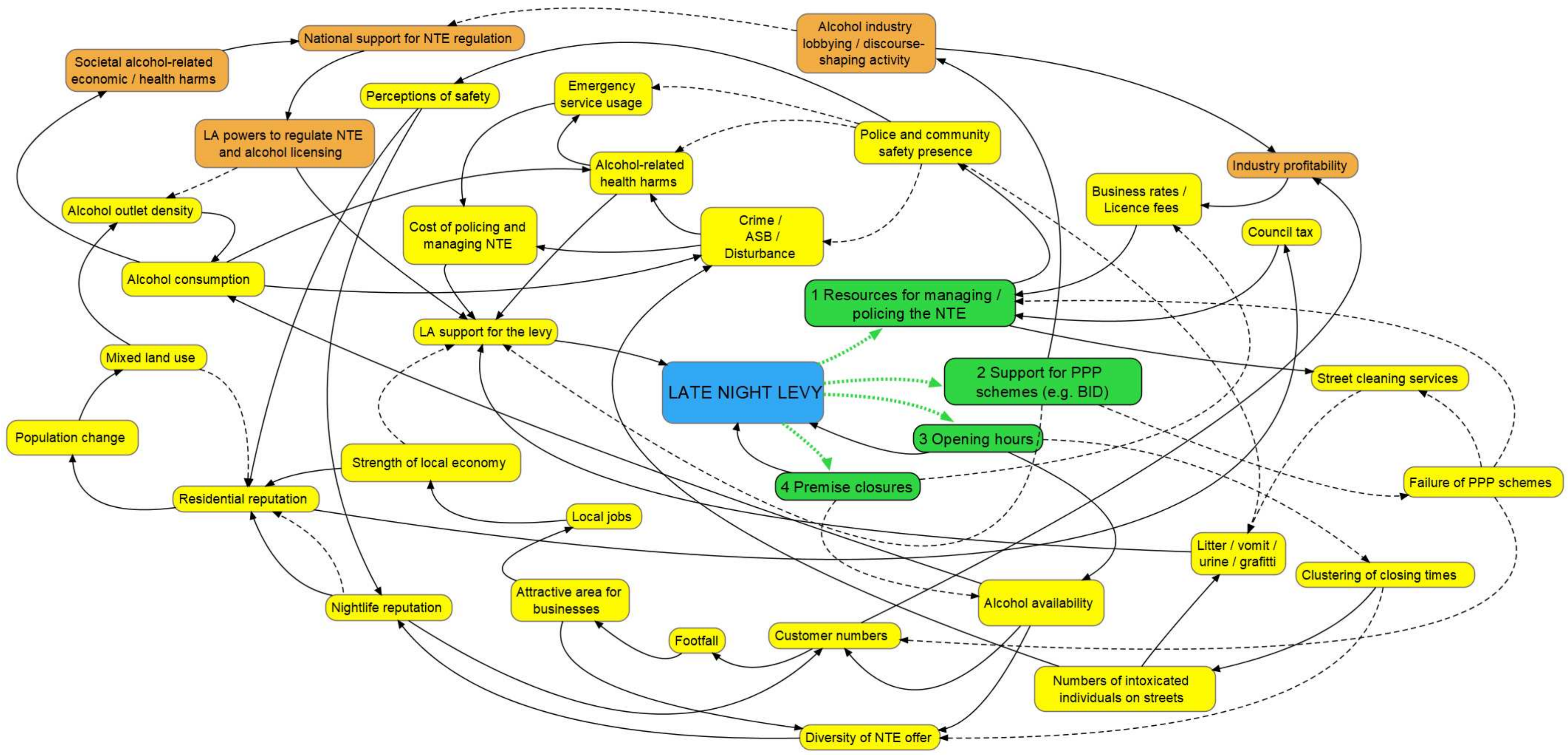

Orange bubbles = national variables; Yellow bubbles = local variables; Green bubbles = immediate theorised impacts stemming from levy introduction
Solid line: positive relationship between variables; Dashed line: inverse relationship between variables; Dotted green line: theorised impacts stemming from the levy introduction
