# Supplementary material for: Addressing alcohol-related harms in the local night-time economy: a qualitative process evaluation from a complex systems perspective
Source: BMJ Open. 2022 Aug 25;12(8):e050913. doi: 10.1136/bmjopen-2021-050913 (PMC9422880; doi:10.1136/bmjopen-2021-050913)
Supplement: Author's manuscript [file bmjopen-2021-050913.draft_revisions.pdf]

BMJ Open is committed to open peer review. As part of this commitment we make the peer review history of every article we publish publicly available.

When an article is published we post the peer reviewers' comments and the authors' responses online. We also post the versions of the paper that were used during peer review. These are the versions that the peer review comments apply to.

The versions of the paper that follow are the versions that were submitted during the peer review process. They are not the versions of record or the final published versions. They should not be cited or distributed as the published version of this manuscript.

BMJ Open is an open access journal and the full, final, typeset and author-corrected version of record of the manuscript is available on our site with no access controls, subscription charges or pay-per-view fees (http://bmjopen.bmj.com).

If you have any questions on BMJ Open's open peer review process please email info.bmjopen@bmj.com

BMJ Open

Addressing alcohol-related harms in the local night-time economy: a qualitative process evaluation from a complex systems perspective

Journal:	*BMJ Open*
Manuscript ID	bmjopen-2021-050913
Article Type:	Original research
Date Submitted by the Author:	03-Mar-2021
Complete List of Authors:	McGill, Elizabeth; London School of Hygiene & Tropical Medicine, Department of Health Services Research and Policy<br>Marks, Dalya; London School of Hygiene & Tropical Medicine, Department of Public Health, Environments and Society<br>Petticrew, Mark; London School of Hygiene & Tropical Medicine, Department of Public Health, Environments and Society<br>Egan, Matt; London School of Hygiene & Tropical Medicine, Department of Public Health, Environments and Society
Keywords:	PUBLIC HEALTH, QUALITATIVE RESEARCH, STATISTICS & RESEARCH METHODS

SCHOLARONE™
Manuscripts

I, the Submitting Author has the right to grant and does grant on behalf of all authors of the Work (as defined in the below author licence), an exclusive licence and/or a non-exclusive licence for contributions from authors who are: i) UK Crown employees; ii) where BMJ has agreed a CC-BY licence shall apply, and/or iii) in accordance with the terms applicable for US Federal Government officers or employees acting as part of their official duties; on a worldwide, perpetual, irrevocable, royalty-free basis to BMJ Publishing Group Ltd ("BMJ") its licensees and where the relevant Journal is co-owned by BMJ to the co-owners of the Journal, to publish the Work in this journal and any other BMJ products and to exploit all rights, as set out in our licence.

The Submitting Author accepts and understands that any supply made under these terms is made by BMJ to the Submitting Author unless you are acting as an employee on behalf of your employer or a postgraduate student of an affiliated institution which is paying any applicable article publishing charge ("APC") for Open Access articles. Where the Submitting Author wishes to make the Work available on an Open Access basis (and intends to pay the relevant APC), the terms of reuse of such Open Access shall be governed by a Creative Commons licence – details of these licences and which Creative Commons licence will apply to this Work are set out in our licence referred to above.

Other than as permitted in any relevant BMJ Author's Self Archiving Policies, I confirm this Work has not been accepted for publication elsewhere, is not being considered for publication elsewhere and does not duplicate material already published. I confirm all authors consent to publication of this Work and authorise the granting of this licence.

For peer review only

Addressing alcohol-related harms in the local night-time economy: a qualitative process evaluation from a complex systems perspective

Elizabeth McGill[1*], Dalya Marks[2], Mark Petticrew[2], Matt Egan[2]

1. Department of Health Services, Research and Policy; London School of Hygiene & Tropical Medicine; London, United Kingdom
2. Department of Public Health, Environments and Society; London School of Hygiene & Tropical Medicine; London, United Kingdom

*corresponding author
London School of Hygiene & Tropical Medicine
15-17 Tavistock Place
London WC1H 9SH
elizabeth.mcgill@lshtm.ac.uk

Word count: 8,657 (excluding abstract, tables, figures and references)

For peer review only

For peer review only - http://bmjopen.bmj.com/site/about/guidelines.xhtml

Abstract

Objectives: English local authorities (LAs) are interested in reducing alcohol-related harms and may use discretionary powers such as the Late Night Levy (LNL) to do so. This study aims to describe and visualise the system in which the LNL operates and to explore how the system, its actors and the intervention adapt and co-evolve over time.

Design: A process evaluation from a complex systems perspective, using qualitative methods.

Setting: A London LA, with a high density of residential and commercial properties, which implemented the LNL in 2014.

Participants: Data were generated through interviews with LNL implementers and alcohol consumers, observations in bars and during LNL patrols, and documentary review.

Intervention: The LNL allows LAs to charge late-night alcohol retailers to manage and police the night-time economy (NTE).

Results: The local system into which the LNL is introduced is characterised by many interrelated variables. Stakeholders theorised the levy to increase resources for policing and managing the NTE, thereby decreasing anti-social behaviour, crime and litter. Stakeholders also theorised that the levy could have unintended consequences by reducing support for public-private partnerships and forcing venues to vary their hours or to close, thereby undermining the levy's viability, clustering closing times, reducing NTE diversity and producing negative economic impacts. In the first two years, levy-funded patrols developed relationships with the licensed trade and the public. The LNL did not undermine public-private partnerships and while some premises varied their hours, these changes did not undermine the intervention's viability, nor significantly cluster venue closing times, nor obviously damage the area's reputation for having a diverse NTE.

Conclusions: This study demonstrates the application of a framework for process evaluation from a complex systems perspective. The evaluation could usefully be extended to measure alcohol-related outcomes and to further consider the interplay between the national and local systems.

Keywords: alcohol; complex systems; qualitative research; process evaluation

Strengths and limitations of this study

- This study used a novel design, drawing on a complex systems perspective, to understand the mechanisms by which the intervention may generate system-wide changes.

For peer review only - http://bmjopen.bmj.com/site/about/guidelines.xhtml

- We generated data through a range of qualitative methods, including interviews, observations and documentary review which allowed us to collect data from a wide range of sources.
- We include data from implementers, NTE users, business owners and staff but not health service workers.
- The evaluation occurred after the intervention started, although many of the documents reviewed were produced prior to implementation.

For peer review only

For peer review only - http://bmjopen.bmj.com/site/about/guidelines.xhtml

INTRODUCTION

In England, alcohol misuse is the largest risk factor for poor health and early mortality for adolescents and adults aged 15-49 years (1), a pattern that is mirrored globally (2). In addition to the health harms associated with alcohol consumption, alcohol contributes to broader societal harms including crime, violence, anti-social behaviour and disorder (3), many of which occur within the context of the night time economy (NTE) (4).

At the turn of the 21st century in England, there was growing public discourse and concern about the rise of the "alcohol-fuelled, consumption-driven, night-time high street" (5 p.466) which was characterised by clusters of late-night establishments and a tension between those enjoying nights out, those employed within or profiting from the NTE and those impacted by violence, anti-social behaviour and nuisance (6,7). A series of sweeping legislative and regulatory changes were made, with claims made that this would create a safer NTE, while generating economic benefits to businesses, the people they employed and to governments (6,8). These changes included the Licensing Act 2003 (enacted in 2005) which transferred responsibility for alcohol licensing from magistrates to local authorities (LAs) and removed fixed closing times for alcohol-retailing venues (8). The sale of alcohol in England is therefore overseen by LAs, also referred to as Councils, through licensing, trading standards and planning bodies (9). In this context, LAs have access to a range of discretionary powers to tackle alcohol-related harms, including Cumulative Impact Policies and Early Morning Restriction Orders, both of which were introduced in the Licensing Act 2003.(10) Another discretionary power, which will be the focus of this evaluation, is the Late Night Levy (LNL) which was introduced in the 2011 Police Reform and Social Responsibility Act. The Act allows Councils to charge alcohol-retailers who have a licence to sell alcohol between midnight and 6 am a fee; the revenue is split between the LA and the police to manage the NTE.

Public health researchers have become increasingly interested in applying a complex systems perspective to analysing the multiple interactions that lead to patterns of health behaviour, outcomes and inequalities across communities (11-13). Where LA's choose to implement the LNL, it is introduced locally into a complex system that interacts with regional, national and international systems. A system is a group of elements, bounded in some way, that interact with each other (14,15). A complex system is one that is characterised by unpredictability and change over time (16,17). Complex systems exhibit emergent properties that cannot be reduced to the behaviour of the individual system elements (18). Elements within a system respond to internal and external system inputs; these responses may feedback on the inputs themselves, either amplifying or dampening their impacts, which may, in turn, create unanticipated or unintended effects (19,20). Analysing a complex system encompasses making sense of the system's trajectory, considering how

For peer review only - http://bmjopen.bmj.com/site/about/guidelines.xhtml

it is influenced by its previous history and the interactions between its elements (17,21). Key concepts from a complex systems perspective, which we consider in this paper, are defined in Table 1.

Table 1: Complex systems concepts

Concept	Definition
**Elements**	Components within a system ('agents', institutions, resources, etc.) (17)
**Boundaries**	The 'limits' or 'bounds' of a given system; boundary judgements may be made by system actors (first-order) or researchers (second-order) (15,22)
**Levels**	The structure of the system; levels may operate horizontally and/or vertically depending on boundary decisions (20,23)
**Relationships and interactions**	Connections between different system elements, within and across system levels, and between elements and the broader context (24)
**Local rules**	The norms and principles that guide interactions between system elements and drive system behaviour (25)
**Perspectives**	The different ways actors within the system may view the system, their goals and actions and boundary decisions (26)
**Non-linearity**	Inputs into a system may lead to a non-correspondingly-sized impact (22)
**Feedback**	Responses that either amplify or dampen the impacts stemming from an intervention and may alter the intervention itself (19)
**Adaptation**	The ways in which system elements and the system as a whole behave in response to internal and external inputs (16)
**Emergent properties**	The emergent, collective behaviour of a system that cannot be reduced to its individual parts (27)
**Co-evolution**	The changes to a system and the broader systems in which it is located, over time (17)
**Unintended consequences**	Processes and impacts that were unanticipated at the design stage of an intervention (20)
**System trajectories**	The evolution of a system over time, which is path dependent or constrained in some ways due to its history (17,21)

Complex systems are characterised by their open boundaries and as a result, they interact with, influence, and are influenced by, other systems (28). From a geographical perspective, they can be characterised by both horizontal and vertical complexity. Horizontal complexity refers to the relationships between system elements, and between systems within the same geographical scale. Vertical complexity refers to the relationships and interactions across geographical scales, with, for example, an emphasis on how international and national systems may influence, constrain and shape local systems (29,30). A recent scoping review of complex systems perspectives applied to alcohol consumption and prevention found that much of the research in this field is conducted in sub-local (e.g. individual, families, social networks) or local (e.g. neighbourhood, town, cities) systems. Far less consideration is given to the ways that the local systems interact with the national or international systems (30).

For peer review only - http://bmjopen.bmj.com/site/about/guidelines.xhtml

A complex systems perspective applied to public health evaluation involves analysing the multiple ways in which a complex system and an intervention interact and influence each other to generate health impacts and health inequalities (31-34). Evaluators might consider interventions as ‘events’ within systems that have the ability to disrupt system behaviour, generating evolving and adaptive patterns of behaviour and emergent outcomes (21,35).

In public health, process evaluations have traditionally been used to understand the mechanisms by which interventions leads to impact, the influence of the broader context on observed variations in impact, as well as to assess intervention fidelity and the quality of implementation (36). Applying a complex systems perspective to a process evaluation can be used to first describe the system, understand its elements, boundaries and the ‘rules’ or norms that govern the behaviour of its elements and the ways in which they interact each other. Following the introduction of an intervention such as the LNL, a process evaluation with a complex systems perspective then aims to understand the mechanisms by which the elements within the system, and the system as a whole adapt and co-evolve in response.

This process evaluation was conducted in one London LA with the dual aims of 1) describing and visualising the system into which the LNL is introduced; and 2) exploring how the intervention acts as an event within the system, with an emphasis on understanding how the system, its actors and the intervention adapt and co-evolve over time.

METHODS

Study design and data generation

We applied a framework for process evaluation using a complex systems perspective to data we collected on the LNL in one LA (32). This evaluation framework consists of two phases: Phase 1 involves producing a static system description and developing theories of how the system may change in response to the intervention; Phase 2 analyses the system as it undergoes change following implementation. The evaluation approach is adaptive and theories of change (ToCs) generated in Phase 1 are intended to inform the evaluative focus of Phase 2. In Phase 2, evaluators should be open to exploring unintended processes that stem from the intervention, that may not have been considered at the design stage or in Phase 1 of the evaluation.

Intervention and setting

The LNL was designed to “empower local areas to charge businesses that supply alcohol late into the night for the extra enforcement costs that the night-time economy generates for police and licensing authorities” (37 p.1). The intervention aims to prevent and address disturbance and crime associated

For peer review only - http://bmjopen.bmj.com/site/about/guidelines.xhtml

with late night drinking. The power is discretionary and LAs can choose, following a period of local consultation, to implement a levy on all establishments in the on- and off-trade that have a licence to sell alcohol between midnight and 6 am. The amount each premise pays is set out in a nationally-determined fee schedule based on the rateable value of the premise and the degree to which the premise was primarily alcohol-led (Table 2). Individual LAs may exempt certain types of premises, such as those operating within a Business Improvement District (BID), and/or offer reductions for premises engaging in schemes such as Best Bar None or PubWatch. BIDs, Best Bar None and PubWatch are business- and alcohol industry-led schemes and businesses voluntarily participate in them. These initiatives are supported by public bodies, including LAs or the Home Office. As shorthand, these schemes will be referred to as public-private partnership (PPP) schemes. The revenue from the levy, following the deduction of administrative costs, must be split with a minimum of 70% going to the police and the remainder to the LA. In 2011, The Home Office estimated that the levy would likely be viable in 94 of the 378 LAs across England and Wales and generate a total net revenue of £12.1m per year (38). The legislation was enacted in 2011 and Newcastle City Council was the first to adopt the levy in November 2013.

Table 2: Late Night Levy charges

**Rateable value**	A: No rateable value - £4,300	B: £4,301 - £33,000	C: £33,001 - £87,000	D: £87,001 - £125,000	E: £125,001 + above	D x 2 multiplier applied to premises in category D that are primarily / exclusively alcohol-led	E x 3 multiplier applied to premises in category E that are primarily / exclusively alcohol-led
**Annual levy charge**	£299	£768	£1,259	£1,365	£1,493	£2,730	£4,440

Source: Home Office 2015 (39)

The LA that is the subject of this process evaluation, held a local consultation on the levy at the end of 2013 and implemented the LNL in late 2014. The levy hours are set at 00:01 to 06:00 and businesses that demonstrate commitment to best practice, as defined by the LA, are eligible for a 30% reduction of the levy fee. Businesses that are a member of the local BID, which requires members to pay a levy separate to the LNL, are neither exempt from the levy nor granted an automatic reduction in the fee. The Metropolitan Police and the LA chose to pool the net amount of levy payments to deliver one broad programme consisting of two different strands: i) additional dedicated police resource to coordinate NTE policing and conduct support and enforcement activity; and ii) a visible street-based patrol service delivered by a police-accredited community safety

For peer review only - http://bmjopen.bmj.com/site/about/guidelines.xhtml

company four nights per week to give assistance to the licensed trade and members of the public. A Late Night Levy Board with representation from licensees oversees the use of funds raised through the levy.

Sampling and data generation

A complex systems perspective encourages evaluators to consider the intervention as a multi-stage process that, in the instance of the LNL, began with changes in national policy, then a local consultation, and finally local implementation. Local delivery processes could continue to interact with national (or other 'non-local') developments. However, this evaluation focuses primarily on the local system: a focus on horizontal complexity. This local focus represents a 'secondary boundary judgement' (22); that is one that is made by evaluators (compared to a 'first order boundary judgement' which is made by actors operating within the system).

The sampling strategy aimed to capture a range of different actors and perspectives within the national and local systems in order to contrast how different actors perceive, respond and adapt to the introduction of the intervention. A large number of system stakeholders were participants in this study but they did not contribute to it design. Given the evaluative focus on the LNL in one LA, the sampling strategy was designed to primarily collect data from local actors through interviews, observations and a documentary analysis. However, recognising that complex systems are open systems, the sampling strategy was intentionally wider than the local system and the documentary analysis also included national data in order to analyse vertical systemic relationships.

In this process evaluation, Phase 1 focuses on the period prior to local implementation, which included the national policy change and the local consultation. Phase 2 focuses on the local implementation stage and is the stage at which we became involved in evaluation. Data collection for Phase 1 was largely retrospective, but based on primary documentary sources generated during the earlier time period. Phase 2 was based on interviews, observations and document analysis collected during the first two years of the levy's implementation.

A range of data collection methods were utilised, including: a review of national and local documents, interviews with those implementing and delivering the LNL locally (n=12), interviews with users of the NTE (n=9), observations of community safety patrols (28.5 hours) which included informal conversations with patrol officers (n=10) and observations in pubs and bars (6 hours). Table 3 shows the documents analysed and their publication dates; Table 4 provides details of the primary data collection. To preserve participant anonymity, generic job roles are presented to remove

For peer review only - http://bmjopen.bmj.com/site/about/guidelines.xhtml

identifying information. Data collection and fieldwork was conducted by EM, a research fellow with experience of a range of qualitative methods and analysis.

Table 3: Documents in documentary review

	Title	Organisation (Date)
National documents	Police Reform and Social Responsibility Bill Research Paper 10/81	House of Commons Library (2010)
	Impact Assessment for the alcohol measures in the Police Reform and Social Responsibility Bill	Home Office (2011)
	Police Reform and Social Responsibility Act (2011)	Act of Parliament (2011)
	The Government's Alcohol Strategy	Home Office (2012)
	Next steps following the consultation on delivering the Government's Alcohol Strategy	Home Office (2013)
	Amended guidance on the late night levy	Home Office (2015)
	The late night levy	House of Commons Library (2015)
	Modern Crime Prevention Strategy	Home Office (2016)
	Policing and Crime Bill: Changes to the Late Night Levy – Impact Assessment	Home Office (2016)
Local documents	Annual Public Health Reports (n=5)	Council (2011 – 2016/17)
	Licensing Policies (n=2)	Council (2011 – 2017)
	LNL Consultation	Council (2013)
	LNL Consultation Responses (n=338)	Council (2014)
	LNL Written Consultation Responses (n=31)	Council (2014)
	LNL Council Meeting Minutes	Licensing Committee (2014)
	LNL Year 1 and Year 2 Reports	Council (2016; 2017)
	LNL Year 1 and Year 2 Reports	Community Safety Company
	BID Annual Reports	BID Board (2015/2016; 2016/2017)

Table 4: Primary data collection

	Participants	Number (details)	Year
Interviews (n=21) (10.4 hours)	Local authority managers and officers relevant to licensing and public health	4 (one individual interview; three interviewed as a group)	2014
	Police officers	3 (individual interviews)	2016
	Community safety officers	5 (two individual interviews; two interviewed as a pair)	2014, 2016
	Users of the NTE	9 (interviewed in pairs or one group of three; Fridays between 20:00 – 21:30)	2016
Observations and informal conversations (35.5 hours)	LNL-funded, community safety patrols; 5 different officers	2 (18.5 hours; 5 officers; Friday 21:00 – 7:00 and Saturday 21:30 – 8:00)	2016
	Non-levy, community safety patrols; 5 different officers	3 (10 hours; 5 officers; Tuesday 6:00-9:00; Wednesday 13:00-20:00)	2016

For peer review only

For peer review only - http://bmjopen.bmj.com/site/about/guidelines.xhtml

	Quarterly review meeting (Local authority managers; community safety company managers)	1 (1 hour; 4 participants)	2016
	Pubs and bars (observation only)	4 (6 hours; Fridays between 19:30 – 22:00)	2016

Documents were identified through online searches which included searches of national and local government websites for documents about the LNL, alcohol and health and crime and safety. In addition, Google searches were undertaken using the term ‘late night levy’. Documents were included if they shed light on the rationale and process for developing and implementing the levy, or reported on the levy following implementation. All documents are located in the public domain. Some of the documents included what might be considered ‘outputs’ in a process evaluation and short-term social and health impacts following intervention implementation. The analysis of these data focused on how they were presented, for what purposes, by which actors and how they suggested early indictors of change stemming from the intervention. We report some of the data from these documents in our Results section.

Interviews with professionals implementing and delivering the LA’s LNL followed a topic guide and asked participants about alcohol-related challenges, their experience of the LNL and the system in which the intervention is located. Topic guides were semi-structured to allow the participant scope to guide the conversation based on their experiences and understanding of the local system and the intervention. The interviews were audio-recorded and transcribed.

Observations were conducted during five community safety patrols partly funded by the LNL in which addressing alcohol consumption and associated harms was either a primary or secondary focus of the patrol. During the patrols, the fieldworker engaged in informal conversations with patrol staff and observed their actions and engagement with individuals or groups, including staff from licensed premises, police officers, users of the NTE, street drinkers and rough sleepers. In total, ten officers conducted the patrols, two of whom were also formally interviewed prior to the patrols. Throughout each patrol, the fieldworker wrote notes when appropriate and, where possible, captured direct quotations from patrol officers. An additional observation was conducted during a LNL review meeting between managers from the LA and community safety company.

In order to better understand how users of the NTE experience the local alcohol system and the LNL, interviews were conducted in pubs and bars. Nine participants were recruited from alcohol-retailing venues; the fieldworker approached groups of 2-3 drinkers for interviews about the local area, particularly its NTE and their views on the LNL. Due to the setting, the fieldworker did not take notes during the interview or record the discussion. Notes, including any direct quotations, were written immediately following each interview.

For peer review only - http://bmjopen.bmj.com/site/about/guidelines.xhtml

Ethical approval for this study was obtained from the s with particular attention paid to consent and safety issues around collecting data in situations where alcohol is consumed.

Patient and public involvement

No patients or public involved.

Analysis

Phase 1

The framework for process evaluation from a complex systems perspective using qualitative methods suggests several questions to guide Phase 1 of the evaluation: 1) What is the system of interest and what are its boundaries? 2) What are the characteristics of the system and how does it behave at the initial timepoint? 3) In what ways could the intervention lead to changes within the system, including changes that may be unanticipated or unintended (32)? The 'Intervention and Setting' section above sets out the local system of interest and its boundaries, which for this evaluation, are the geographical boundaries of the LA.

The analysis began with an in-depth reading of all transcripts, fieldnotes and documents and a deductive approach to coding the data was undertaken, guided by a number of concepts from systems thinking which included: elements, boundaries, levels, relationships and interactions, perspectives and history (see Table 1). The coding process was used to make sense of the national and local histories that created the conditions for the development and implementation of the LNL, the goals of different actors and how their perspectives influenced their views towards the levy. The data were then utilised to develop theories of change, with a particular emphasis on stakeholder perspectives and how they aligned or contradicted each other in order to understand how the intervention may lead to changes within, and beyond, the system into which it is introduced.

A system map was developed utilising Visual Understanding Environment (VUE) software (40) (Figure 1). The choice of the geographical boundary was reflected in the map, but the analysis also sought to understand the influences on the implementation of the levy from beyond the LA geographical boundaries. A list of variables relevant to the LNL, nationally and locally, was independently generated by two researchers (EM and ME) from the coded data. The variables and the relationships between them were then represented visually on a map, noting whether variables were positively or inversely related. The map was used to visualise the system, aid understanding of the 'initial conditions' into which the LNL was introduced and to generate hypotheses on the ways in which the levy was theorised to generate change within the local system. The analysis of Phase 1 was completed before the Phase 2 analysis so that it could inform the analytical focus for Phase 2.

For peer review only - http://bmjopen.bmj.com/site/about/guidelines.xhtml

For peer review only

Phase 2

In Phase 2 of the process evaluation framework, the evaluator seeks to understand how the system and the intervention itself change following implementation, exploring the mechanisms by which change occurs (32). The theories of change developed in Phase 1 were used to guide the analysis. In Phase 2, the focus of the evaluation was on the new actors that were introduced into the system with levy funding. There was a simultaneous focus on the system elements and the system as a whole, considering how they adapt and co-evolve over time, disrupting the local system rules and patterns of behaviour. The coding and analysis were led by EM, with analytical discussions taking place across the research team. NVivo 12 was used to aid the data analysis (41).

RESULTS

Phase 1: system description and theories of change

What are the characteristics of the system and how does it behave at the initial timepoint?

The local system structure and the relationships within it are presented in Figure 1. The system map is comprised of a range of variables that characterise the local system; each variable is represented by a bubble; the lines represent relationships to other system variables and whether the two variables are positively (solid line) or inversely (dashed line) related to each other. A description of each variable is provided in Table 5, along with the other system variables it is directly related to and/or from. The final column in the table provides an excerpt of data to illustrate each variable. The system represented in the diagram is bounded so that it contains the elements operating within the geographical area of the LA. However, the local system is a complex system and therefore these boundaries should be considered 'open'.

Figure 1 about here

For peer review only - http://bmjopen.bmj.com/site/about/guidelines.xhtml

For peer review only

Table 5: System variables and relationships within the local system

System	Variable	Description	Connected to/from (positive or inverse relationship)	Example(s) (source)
National	National support for NTE regulation	The degree to which national policymakers support the introduction and extension of regulation in the NTE.	To: LA powers to regulate NTE and alcohol licensing (+); societal alcohol-related economic/health harms (+)<br><br>From: alcohol-industry lobbying / discourse-shaping activity (-)	"The Government intends to rebalance the licensing regime to enable local 'Licensing Authorities' (LAs) and the police to clamp down on alcohol-related crime and disorder, particularly late at night; to allow wider considerations and the views of local communities to be taken into account in licensing decisions; to protect children from the harm of alcohol; and introduce a late night levy to help pay for other costs caused by late-night drinking." (Impact assessment for Police Reform and Social Responsibility Bill 2010)
	LA powers to regulate NTE and alcohol licensing	The specific powers available to LAs to regulate the NTE and control the provision of alcohol through alcohol licensing powers, such as the LNLs, CIPs, and EMROs.	To: LA support for the levy (+); alcohol outlet density (-)<br><br>From: national support for NTE regulation (+)	"The late night levy is a discretionary power enabling licensing authorities in England and Wales to raise a contribution towards policing the late-night economy from holders of premises licences or club premises certificates." (House of Commons, Late Night Levy Briefing, 2015)
	Societal alcohol-related economic and health harms	The societal harms associated with alcohol, including the associated healthcare costs, productivity losses and population-level health harms.	To: national support for NTE regulation (+)<br><br>From: alcohol consumption (+)	"Alcohol misuse also costs the United Kingdom economy an estimated £7.3 billion a year in lost productivity and the National Health Service in England an estimated £3.5 billion a year. In England, over 15,000 people die from alcohol-related illnesses each year." (Next steps on delivering the Government's Alcohol Strategy, 2013)
	Alcohol industry lobbying / discourse-shaping activity	Activities the alcohol industry engages in to lobby for and frame debates in terms favourable to their corporate interests.	To: industry profitability (+) ; national support for NTE regulation (-)<br><br>From: support for PPP schemes (+)	"We believe that the Council should maintain its current voluntary best practice approach which is delivering real results and crucially is focused on eliminating the source of the problems rather than simply paying for any clean up.[…]<br><br>This is in line with the National Alcohol Strategy which states that targeted action taken voluntarily by pubs and clubs themselves is most effective in curbing irresponsible drinking and associated drunken violence. The Home Secretary, Theresa May, in publishing the strategy suggested that a legislative approach, either national or local, was a 'sledgehammer' which all too often misses its target and that a partnership approach was more effective." (Consultation response, trade organisation representing on-licence premises).

For peer review only

For peer review only - http://bmjopen.bmj.com/site/about/guidelines.xhtml

System	Variable	Description	**Connected to/from** (positive or inverse relationship)	Example(s) (source)
**National**	Industry profitability	The financial gains realised by the alcohol industry.	To: business rates/licence fees (+)<br><br>From: alcohol-industry lobbying / discourse-shaping activity (+); customer numbers (+)	"[The LNL] will impose a significant further cost burden on the hospitality industry in the [local] area when the overall costs the industry must pay whether food, drink, labour and taxes contribute to rise and customers' real incomes shrink impacting on profitability". (Consultation response, operator of managed pubs).
**Local**	Alcohol outlet density	Concentration of premises selling alcohol for consumption on or off the premises.	To: alcohol consumption (+)<br><br>From: LA powers to regulate the NTE and alcohol licensing (-); mixed land use (+)	"The number of licenced premises continues to grow rapidly so that [LA] has one of the highest densities of pubs, bars, clubs and off licences in the country and second highest in London after the City of Westminster." (Licensing Policy 2013-2017)<br><br>"I: So from your perspective, what would you say are the major kind of alcohol related challenges in [LA]?<br>R: *There are too many licensed premises."* (Interview, Police licensing officer)
	Alcohol consumption	The amount of alcohol an individual consumes. Can be measured in the context of a single drinking event, or to describe patterns of consumption.	To: crime / ASB / disturbance (+); alcohol-related health harms (+)<br><br>From: alcohol outlet density (+) ; alcohol availability (+)	"Drinking above recommended maximum limits has become much more common over the past two decades, particularly among younger and middle aged men and women of all social groups. This trend is mirrored in [LA]. […] The effects of the increase in alcohol consumption seen over the past two decades are now being seen in the significant increases in alcohol-related attendances and admissions in the NHS." (Licensing Policy 2011-2014)<br><br>"Alcohol consumption is increasingly identified as a major factor behind violent crime and disorder in the borough with serious consequences to victims, businesses and local communities." (Licensing Policy 2013-2017)
	Alcohol-related health harms	Individual health harms experienced as a result of alcohol consumption.	To: LA support for the levy (+); emergency service usage (+)<br><br>From: alcohol consumption (+); crime / ASB / disturbance (+); police and community safety presence (-)	"In reviewing our Licensing Policy we have been mindful that [LA's] residents suffer from high levels of alcohol-related ill health and early deaths." (Licensing Policy 2013-2017)<br><br>"Studies assessing the effectiveness of limiting the density of alcohol outlets showed greater alcohol outlet density to be associated with increased alcohol consumption and harms including injury, violence, crime and medical harm. Small numbers of concentrated problematic nightlife venues often cause a large proportion of alcohol-related harm, violence and injuries in city centres." (Public Health Report 2012)

For peer review only - http://bmjopen.bmj.com/site/about/guidelines.xhtml

System	Variable	Description	Connected to/from (positive or inverse relationship)	Example(s) (source)
Local	Mixed land use	The degree to which areas within the LA are mixed residential and commercial.	To: residential reputation (-); alcohol outlet density (+)<br><br>From: population change (+)	"[LA name] is a densely occupied area, with no clear delineation between residential and commercial premises and the Licensing Authority will need to carefully balance the conflicting needs of residents, patrons and businesses in relation to the introduction of flexible opening hours for the sale and supply of alcohol and late night refreshments." (Licensing Policy 2011-2014)
	Population change	The demographic characteristics of the local population and its rate of change.	To: mixed land use (+)<br><br>From: residential reputation (+)	"[LA], however, is undergoing a process of rapid change and this is likely to continue. The number of people living here has grown from 178,000 in 2001 to an estimated 199,130 in 2010. One of the reasons for this is the increase in the number of young adults who are moving into inner London, and starting families. […] This has turned many parts of the borough, which were previously exclusively commercial, into mixed-use hubs incorporating commercial and residential premises in very close proximity." (Licensing Policy 2011-2014)
	Residential reputation	The extent to which the local area is viewed as a desirable place to live.	To: population change (+); council tax (+)<br><br>From: perceptions of safety (+); mixed land use (-); nightlife reputation (+/-); strength of the local economy (+)	"I spoke to the public health lead for alcohol in [neighbouring LA] last week and he was saying this, exactly. […] Having all those bars, having all those people drinking, that's what they call regeneration, whereas in [this LA], my impression what they call regeneration is a good place to live, not a good place to party." (Interview, Public Health specialist)<br><br>"[LA name's] nightlife is marvellous and one of the reasons I love being a resident here." (Consultation response, resident)
	Nightlife reputation	The extent to which a local area is viewed as a desirable place to go out.	To: residential reputation (+/-); customer numbers (+)<br><br>From: perceptions of safety (+); diversity of NTE offer(+)	[Participant] almost always goes out in [LA] when he goes out. He likes the variety of places and that you can get fairly cheap drinks and still be in Zone 1. (Excerpt from fieldnotes)<br><br>"[LA] has a reputation for its vibrant nightlife and the council, along with its partners the Metropolitan Police and London Fire Brigade recognise that many people are working hard to ensure that [LA]'s clubs, pubs and bars provide a safe and attractive venue for customers." (Licensing Policy 2011-2014)
	Cost of policing and managing the NTE	The resources required to manage and regulate the NTE; includes police, community safety, street cleaning, licensing and trading standards.	To: LA support for the levy (+)<br><br>From: emergency service usage (+); crime / ASB / disturbance (+)	"[LA] has a well established night-time economy that has continued to grow since the introduction of the Licensing Act in 2005. The number of late-night and 24 hours premises is high and they are spread across the borough. The costs of policing the late night economy are substantial." (Consultation response, Executive Member for Community Safety).

For peer review only

System	Variable	Description	Connected to/from (positive or inverse relationship)	Example(s) (source)
Local	LA support for the levy	The degree to which key stakeholders responsible for local area, support the need to introduce the levy; includes elected members and responsible authorities.	To: Late Night Levy<br><br>From: LA powers to regulate NTE and alcohol licensing (+); cost of policing and managing the NTE (+); alcohol-related health harms (+); strength of the local economy (-); litter / vomit / urine / graffiti (+); support for PPP schemes (-)	"The supporting statement for the consultation at Appendix A sets out why the Council, supported by the police, believes the levy is necessary and this is still felt to be strong and compelling." (Consultation response, Executive Member for Community Safety).
	Perceptions of safety	The extent to which individuals feel safe in their local environment; includes both residents and visitors perceptions.	To: residential reputation (+); nightlife reputation (+)<br><br>From: police and community safety presence (+)	"[LA] is a safe place to socialise, this is my perception and common among my going out friends" (Consultation response)<br><br>"I've lived in a lot of places – [LA] and, in particular, [popular NTE area] are hardly a war zone!" (Consultation response)
	Emergency service use	The use of emergency services, including ambulances, A&E and police.	To: cost of policing and managing the NTE (+)<br><br>From: police and community safety presence (-); alcohol-related health harms (+)	"Generally, as density of licensed premises in LA increases so does the number of ambulance callouts (shown on map) and also levels of alcohol-related crime." (Public Health Report, 2012)
	Crime / ASB/ Disturbance	The prevalence of crime, anti-social behaviour, disturbance and nuisance that is fuelled by alcohol consumption.	To: alcohol-related health harms (+); cost of policing and managing the NTE (+)<br><br>From: alcohol consumption (+); police and community safety presence (-); numbers of intoxicated individuals on the street (+)	"A comparison of alcohol related violence prior to deregulating licensing hours in 2004 with 2011 shows that in 2004 alcohol related crime peaked between the hours of 11pm and midnight. By 2011 the peak hours for alcohol related crime had expanded and shifted to midnight to 5am with a corresponding 600% increase in alcohol related crime." (Licensing Policy 2013-2017)<br><br>"I'm very pleased to see [name] council taking this initiative. I live in [area] which has more than enough pubs and clubs and where noisy customers in the street are a problem in the small hours." (Consultation response, resident)

For peer review only - http://bmjopen.bmj.com/site/about/guidelines.xhtml

System	Variable	Description	Connected to/from (positive or inverse relationship)	Example(s) (source)
Local	Police and community safety presence	The visible presence of police and community safety officers on the streets and in and around licensed premises.	To: emergency service usage (-); alcohol-related health harms (-); crime / ASB / disturbance (-); perceptions of safety (+); litter / vomit / urine / graffiti (-)<br><br>From: resources for managing / policing the NTE (+)	"From October 2012, a new late night levy will empower local areas to make those businesses that sell alcohol late into the night contribute towards the cost of policing and wider local authority action. This will help enable visible and proactive policing at targeted locations where there are local needs." (The Government's Alcohol Strategy, 2012)<br><br>"The proposals we have for spending the levy are to: · have a uniformed presence patrolling at weekends to deal with enforcement issues, drunkenness, street urination, rowdy and nuisance behaviour and assistant to vulnerable individuals identified. · Provide additional policing to support operations targeting crime and disorder. […]" (LNL Consultation, 2013)
	Resources for managing / policing the NTE	The funds, staff and equipment needed to police and manage the NTE.	To: police and community safety presence (+) ; street cleaning services (+)<br><br>From: Late Night Levy; business rates / licence fees (+); council tax (+); failure of PPP schemes (-)	"The fees set for licensed premises have not increased since their introduction in 2005. During the intervening eight years, the demands for services have increased along with the number of premises opening late and it is now necessary to look to alternative ways of financing the services needed to manage the impacts." (LNL Consultation, 2013)<br><br>"[The Late Night Levy] could potentially curb some of the more dangerous behaviour and sales of alcohol, as well as provide the Council and Police with additional resources in recognition of the extra costs involved in policing, monitoring and cleaning the areas around premises with late alcohol licences." (Consultation response, political party)
	Business rates / license fees	The fees that premises pay to the LA; the business rate is based on the rateable value of the premise and the license fee is applicable for all premises with a license to sell alcohol for on- or off-premise consumption.	To: resources for managing / policing the NTE (+)<br><br>From: premise closures (-); industry profitability (+)	"Like I said, I don't want boarded up buildings. I can't have a whole area of [LA]. Because I live here, it's not good for the economy, it's not good for crime and disorder, you know. It's, it's not good, you know, it's not good for the Council, you know, we don't get business rates if premises are empty." (Interview, Licensing officer)<br><br>"The council and police should already be budgeting for such a problem. Bars and pubs already pay a licence fee to the council – what's that currently paying for?" (Consultation response, licence holder)
	Council tax	Tax paid by households to LA based on the value of the property to fund LA services.	To: resources for managing / policing the NTE (+)<br><br>From: residential reputation (+)	"Business rates/council tax is already astronomically high so I don't see why this shouldn't already be covered?" (Consultation response)

For peer review only - http://bmjopen.bmj.com/site/about/guidelines.xhtml

System	Variable	Description	Connected to/from (positive or inverse relationship)	Example(s) (source)
Local	Support for PPP schemes	The degree to which alcohol retailers and the alcohol industry support voluntary partnerships with the police and local authority, as well as other businesses, including the BIDs, PubWatch, Best Bar None, etc.	To: failure of PPP schemes (+); alcohol-industry lobbying / discourse-shaping activity (+); LA support for the levy (-)<br><br>From: Late Night Levy	“It penalises the hard work that many operators have undertaken in partnerships with the local authority and the police.” (Consultation response, licence holder)<br><br>“[LA] has engaged in active partnership working with its licensed premises so as to ensure high standards of management that will prevent nuisance and crime. It has participated in Central Government initiatives, held training events and developed a number of best practice schemes, including the Community Alcohol Partnership programme […] There are six Pubwatches operating throughout the borough though this still sees only a small percentage of businesses actively engaging with the Council and police. Despite this engagement and the standards achieved within premises, [LA] continues to have a high level of alcohol related crime.” (Consultation response, Executive Member for Community Safety).
	Failure of PPP schemes	The number of PPP schemes that licensees choose to no longer support.	To: street cleaning services (-); resources for managing / policing the NTE (-); customer numbers (-)<br><br>From: support for PPP schemes (-)	“It is also unrealistic in the current economic climate to expect operators to have funds available to support involvement in voluntary initiatives AND pay the late night levy. Where no discount is available to encourage participation in such schemes and to provide some financial relief for doing so, such schemes will wither on the vine as membership falls away.” (Consultation response, Pub company)
	Opening hours	The hours until which a premise is licensed to sell alcohol.	To: alcohol availability (+); clustering of closing times (-); Late Night Levy<br><br>From: Late Night Levy	“[…] we understand that a large number of our members’ business will choose to voluntarily restrict their hours. Generally, it is only dedicated late night businesses which will generate sufficient revenues after midnight to justify retaining licensing hours within the levy period. Many traditional pubs or restaurants will have later closing times but will not regularly use them and, as the levy is based on permission not use, will therefore voluntary relinquish them rather than face an additional cost.” (Consultation response, trade association representing on-licence trade)
	Clustering of closing times	The degree to which premises close at the same time.	To: numbers of intoxicated individual on streets (+); diversity of NTE offer (-)<br><br>From: opening hours (-)	“One consequence of the levy is likely to be that a significant number of premises will reduce their hours to 12 midnight resulting in a return to a non staggered closing time culture, contrary to government policy.” (Consultation response, supermarket chain).

For peer review only - http://bmjopen.bmj.com/site/about/guidelines.xhtml

System	Variable	Description	Connected to/from (positive or inverse relationship)	Example(s) (source)
Local	Number of intoxicated individuals on the street	The number of people out on the street who have been drinking.	To: crime / ASB / disturbance (+); litter / vomit / urine / graffiti (+)<br><br>From: clustering of closing times (+)	"The Council should also consider the risk of a defacto uniform terminal hour for premises arising as operators cut back their premises licences to cease at whenever the levy starts to bite. We have highlighted the actual experience of [LA with a levy] above. This will mean more customers on the street at the same time with resultant pressure on resources such as taxis, fast food outlets and policing." (Consultation response, Pub company)
	Alcohol availability	The ease and convenience of purchasing availability; availability is comprised of the density of licensed premises (physical availability), the hours in which alcohol is sold (temporal availability), and the cost of alcoholic beverages (economic availability).	To: diversity of NTE offer (+); customer numbers (+); alcohol consumption (+)<br><br>From: opening hours (+); premise closures (-)	"Availability of and access to alcohol has an important influence on levels of alcohol consumption. Generally speaking, changes in the availability of alcohol tend to be reflected sooner or later in changes in levels of alcohol consumption and alcohol-related harm." (Public Health Report, 2012)
	Premise closures	The number of alcohol-retailing premises who cease operating.	To: alcohol availability (-); business rates / licence fees (-); Late Night Levy<br><br>From: Late Night Levy	"There is a very real risk that [LA] Council will be responsible for premises closures, loss of jobs and income from rates (with higher outgoings in dealing with those people put out of work) from over-taxing those premises already struggling to cope with the burden they currently have." (Consultation response, Pub and bar operator)
	Street cleaning services	The services to keep streets clean, including clearing rubbish and recycling, washing away vomit and urine, and removing graffiti.	To: litter / vomit / urine / graffiti (-)<br><br>From: resources for managing / policing the NTE (+)	"The proposals we have for spending the levy are to: […] provide additional cleaning and service such as litter removal, graffiti removal and cleaning." (LNL Consultation, 2013)
	Litter/vomit/urine/graffiti	Quality of the physical environment; physical manifestations of incivility.	To: LA support for the levy (+)<br><br>From: street cleaning services (-); police and community safety presence (-); number of intoxicated individuals on the street (+)	"the Licensing Authority has seen an increase in concerns raised by local residents, Councillors and local businesses about the impact that the night time economy is having on the local environment in this area. Typical issues of concern include: • Public urination • Litter • Noise nuisance from patrons of licensed premises • Drug dealing • Thefts • Damage to property and vehicles • Obstruction of the public highway" (Licensing Policy, 2011-2014)<br><br>"For instance, a significant majority [of] litter on the streets at night comes from premises not licensed to tell alcohol at that time, such as takeaways and shops with late opening hours. It is illogical and unfair to make payment for cleaning up the sole responsibility of premises selling alcohol." (Consultation response, Pub company and brewer).

For peer review only - http://bmjopen.bmj.com/site/about/guidelines.xhtml

System	Variable	Description	Connected to/from (positive or inverse relationship)	Example(s) (source)
Local	Customer numbers	Number of patrons frequenting licensed premises.	To: footfall (+); industry profitability (+)<br><br>From: alcohol availability (+), nightlife reputation (+); failure of PPP schemes (-)	"Any reduction to its licensed hours made by venue operators will unquestionably have a detrimental impact on other local businesses in the area, including [LA's] many eateries, as there are likely to be fewer customers in the area for a shorter period of time, thus further affecting the financially lucrative late night economy." (Consultation response, licensee).
	Footfall	The number of individuals in the LA on a given night.	To: attractive area for businesses (+)<br><br>From: customer numbers (+)	"We are bound to ask whether the Council has considered the consequences of closures not just to the licence holders in [LA] but the migration of customers into the neighbouring boroughs which will not only affect the earlier evening trade but will also have the effect of damaging other businesses through lower footfall in that earlier evening." (Consultation response, Hospitality company)
	Attractive area for business	The degree to which an area is perceived as attractive for a business, includes number of residents and visitors, other types of establishments and 'friendliness' of Council to businesses.	To: local jobs (+); diversity of NTE offer (+)<br><br>From: footfall (+)	"The Council should consider it decision very carefully before [LA] becomes a place that becomes unattractive as a place to trade. [Pub company] will decide its future expansion and growth plans within London on the basis of how business friendly the incumbent Council is in any particular area." (Consultation response, pub company)
	Local jobs	The number of jobs in a local area, some of which are in the alcohol-retailing and hospitality sector.	To: strength of the local economy (+)<br><br>From: attractive area for businesses (+)	"This is an unfair tax on vulnerable businesses who provide wealth and jobs for the borough." (Consultation response, licence holder)<br><br>"But we're a poorer borough, we need people to have jobs, we don't want boarded up or empty premises". (Interview, Licensing officer)
	Strength of the local economy	The degree to which there are wealth generating activities in the LA. The NTE is one contributor to the local economy.	To: LA support for the levy (-); residential reputation (+)<br><br>From: local jobs (+)	"The council recognises that the entertainment and alcohol industry contributes to the borough by providing a variety of opportunities for entertainment as well as employment and career opportunities." (Licensing Policy 2011-2014)<br><br>"This is a significant additional cost for businesses to bear and it will affect profitability and viability for many. Some will choose to close earlier to avoid paying this, with knock on effects on turnover, GVA to the local economy and employment patterns." (Consultation response, trade association representing on-licence trade)

For peer review only

System	Variable	Description	Connected to/from (positive or inverse relationship)	Example(s) (source)
**Local**	Diversity of NTE offer	Different types of premises operating in the NTE, including: pubs, bars, clubs, restaurants, live music venues, off-licences, supermarkets, clubs, hotels and the characteristics of those premises (e.g. catering to different types of clientele).	To: nightlife reputation (+)<br><br>From: alcohol availability (+); clustering of closing times (-); attractive area for businesses (+)	"The council is keen to preserve a diverse mix of premises through the borough and wants to work with businesses, residents and partners through its Licensing Policy to resist the saturation of licensable premises of similar types within identified local areas." (Licensing Policy 2011-2014)<br><br>"We feel that there has not been enough research carried out into the benefits of a mixed and diverse night time economy in [LA} which could stagnate on the introduction of a levy. Premises where alcohol does not contribute significantly to profit but is offered after midnight may well close early. This will harm the diversity of offer, leaving only larger pubs and nightclubs willing to pay the levy." (Consultation response, pub company and brewer)

Abbreviations: A&E: Accident and Emergency; BID: Business Improvement District; CIP: Cumulative Impact Policy; EMRO: Early Morning Restriction Order; LA: Local Authority; LNL: Late Night Levy; NTE: night time economy; PPP: public-private partnership

For peer review only

For peer review only - http://bmjopen.bmj.com/site/about/guidelines.xhtml

In what ways could the intervention lead to changes within the system?

The following section explores ways in which stakeholders viewed the levy as having the ability to disrupt the local system through four theories of change.

ToC 1: Increased resources

The primary theory of change, as articulated by those designing, implementing and delivering the intervention, was that the LNL would increase the resources available to police and manage the NTE, which would be used for street-based community safety and policing and additional street cleaning services. These services, would, in turn, lead to a number of positive impacts for residents, visitors and commercial actors (Figure 2):

> *This will produce additional funding for the council and police to use to address the impacts and strains on local services that occur between midnight and 6am in [LA]. […] we believe that the LNL can be used to reduce the instance of crime, disorder and anti-social behaviour during the levy hours as well as improve the local environment.* (LNL Consultation, 2013)

Figure 2 about here

Some residents and visitors further described the mechanism by which such change would occur, placing an emphasis on the additional police and community safety presence; for example, one woman we interviewed in a pub believed more police on the street "means I can walk home safely at 2 am." Others described police as a deterrent for anti-social behaviour and noise:

> *I live on a side street of a late licensed premise and am woken up between 2 am and 7 am regularly every Saturday and Sunday morning. I don't think they realise the noise they're making so if there was a police presence I don't think they'd be as boisterous.* (Consultation response, resident)

It was theorised that a safer NTE would enhance the overall reputation of the area, driving up visitor numbers and encouraging individuals to spend more money in local establishments. Implicit in this argument was that the levy fee would be easily offset by increased customer numbers, thereby making the levy beneficial for both commercial actors and the LA:

> *the money's going to pay for more policing, and [licensees] get more policing and that is beneficial for them because the safer an area is, the more people that come to the area and the more money that gets spent and the more money they make.* (Interview, Community safety officer)

For peer review only - http://bmjopen.bmj.com/site/about/guidelines.xhtml

ToC 2: Reduced support for PPP schemes

A second theory of change, articulated by businesses, was that licensees would be disinclined to continue to support PPP schemes because the resources do so would be re-directed to paying the levy:

> *If operators do choose to pay the levy then it will impact on funding they can provide for partnership initiatives such as BIDs, Pubwatch and Best Bar None which the Council should look to support and promote in preference to a levy.* (Consultation response, Pub company)

Many licensees in particular expressed concern about the LNL's impact on the BID, which funds dedicated police officers and cleaning services. In consultation responses, licensees agued they would vote against the BID when it came up for renewal, which in turn, would cause the BID to fail: (Figure 3): "As a BID payer if the levy were to come into force I would be voting no the next time the bid comes up for tender*"* (Consultation response, licensee).

Figure 3 about here

If the BID were to fail as a result of the LNL, it was theorised that there would be a range of unintended consequences in the local system. These included reducing the overall resources available to manage and police the local area and damaging economic impacts because the BID is intended to work to create an environment that encourages residents and visitors to the area:

> *It is a possibility that nearly 40 licensed premises in the [local area] BID area will not vote for the BID again if this means that they pay two levies instead of only one. A BID needs a majority by numbers and also rateable value to succeed. A failure to achieve either one of these would therefore, jeopardise the provision the BID makes for policing and cleaning as well as what we do to ensure a good shopping environment for local people, Christmas lights, hanging baskets, support for community events and much more.* (Consultation response, Pub manager)

Any negative impact of the levy on the BID was theorised to also have impacts beyond the boundary of the local system. The BID Board argued that this should be considered within the Mayor of London's goal to increase the number of BIDs throughout London:

> *BIDs are burgeoning in London and the Mayor has set a target for a number of additional BIDs by 2015. It would be a loss, not just to [LA] but to London as a whole should [BID name] not get re-elected and become the first BID in London to fail.* (Consultation response, BID Board)

For peer review only - http://bmjopen.bmj.com/site/about/guidelines.xhtml

ToC 3 and 4: Premises will (3) vary hours or (4) close due to unwillingness or inability to pay the Levy

In response to the Council's consultation, 42% of businesses reported they would voluntarily change their permitted licensing hours in response to the introduction of the levy. A smaller number argued that the levy would force some businesses to close as they became economically unviable. These possible responses were theorised to lead to a range of unintended consequences, including undermining the levy, re-introducing a 'terminal hour,' reducing the diversity of late-night provision and ultimately generating negative economic consequences to the local area in the form of reduced employment and local investment.

Only two consultation responses, submitted by Public Health and the Clinical Commissioning Group, considered the LNL in terms of its ability to reduce alcohol consumption and associated harms by restricting the availability of alcohol. In contrast, all other system actors discussed the levy in terms of addressing the harms associated with acute intoxication, focusing primarily on disturbance, anti-social behaviour, crime, and to a far lesser degree on health-related indicators such as ambulance call-outs or hospital admissions. In this sense, discourses around reducing or preventing alcohol consumption (primary prevention) were largely absent, with a focus instead on making the NTE a safer space for consumption and the possible economic and cultural impacts of the levy.

Figure 4 about here

As businesses shut early, or closed entirely in response to the levy, some actors theorised that the LA would fail to generate sufficient revenue to provide the new proposed services: "We remain to be convinced that the LNL will raise the amounts of money anticipated, as a significant number of permissions within [LA] are likely to be withdrawn, by way of the free minor variation procedure." (Consultation letter, Pub company and brewer). This represented an example of a negative feedback loop; as fewer businesses remained to contribute to the levy through late-night provision, the ability for the levy to continue as an intervention would be jeopardised.

If businesses varied their operating hours to avoid the levy, some in the licensed trade argued that this could effectively re-introduce a 'terminal hour' whereby many premises close at the same time, which would lead to an increase in crime and anti-social behaviour:

> *If a number of premises reduce their hours as a result of the levy, this could potentially create anti-social behaviour issues with a large number of premises closing at the same time and a return to the spike of crime, disorder and nuisance and midnight observed across the country prior to the introduction of the Licensing Act 2003.* (Consultation response, Trade organisation representing on-licence premises)

For peer review only - http://bmjopen.bmj.com/site/about/guidelines.xhtml

Some actors expressed concern that smaller, independent businesses, as well as those which are not alcohol-led, would be most affected by the levy, leaving a less diverse NTE dominated by pub chains and clubs. A reduction in diversity was theorised to make the LA less attractive, which, in turn, could have negative economic impacts as customers choose to go elsewhere, moving beyond the boundaries of the local authority:

> *Many operators will have to curtail their hours irrespective of the economic consequences, thereby reducing the number of post-midnight premises in the borough. […] visitors to the Borough's late night economy [would be] choosing other areas of London where no such restrictions apply with obvious economic consequences for [LA]'s late night economy and the businesses that rely on it.* (Consultation response, Operator of managed pubs)

In underscoring how elements of the system are interconnected, a number of businesses suggested that the LNL would have negative economic impacts that affect more than just late-night alcohol retailers, making the LA a less appealing area to operate a business:

> *I am currently looking at sites in the borough; I run a high end food and drink offer, if this levy is introduced I would have to look if the operation could still be viable. My venues do not run beyond midnight but I understand that early evening venues are intrinsically linked to the later venues and if these were to close or relocate it would reduce footfall in the areas affected*. (Consultation response, prospective licensee)

Ultimately many businesses argued that the LNL, through changes to opening hours, lower profit margins, premise closures and lack of investment in the local area, would result in negative economic consequences and job losses for the LA as a whole:

> *The council will further kill off the high street if they implement this levy. Pubs and bars will re-locate to other nearby locations where the levy is not in place and lose a number of job opportunities for local people. I thought the council's major objective as to increase employment opportunities for local people, not decrease it.* (consultation response)

Phase 2: early implementation and mechanisms of system change

The levy began on November 1, 2014 and in the first year, fees were collected from 338 licence holders.

ToC 1: Increased resources

The key theory of change as described by those who designed and implemented the levy, was that it would bring in additional resources to manage and police the NTE (Figure 2). In the first year the levy raised £397,278 and in the second year £377,122 (Council LNL Year 1 and 2 Reports). While these figures were lower than the Council's projected £450,000, the Council described these as sufficient to plug an "an identified gap" in managing and policing the NTE (Council, LNL Year 1 and 2 Reports).

For peer review only - http://bmjopen.bmj.com/site/about/guidelines.xhtml

The additional resources were utilised to fund an NTE-specific police team and a four-person community safety patrol, delivered by a police-accredited, private company, that worked Thursday – Sunday nights from approximately 8 pm – 8 am.. The new community safety service is the primary focus of Phase 2 of this process evaluation; an overview of the structure of the new service is provided in Table 6, along with output data from the service provider's annual reports.

Table 6: Community safety service

Patrol description		
**Patrol description:** The patrol met at 8 pm and conducted a 'scan' of the borough, driving down main roads and stopping to address any issues they identified, such as visible pre-loading. At 10 pm the officers attended a briefing at the police station which included: 1) a police briefing for all officers on duty and 2) a NTE briefing for the NTE police patrol and the community safety officers. Following the briefing, the community safety officers patrolled the borough throughout the night, conducting a number of 'taskings' (which came from the Police, the Licensing Team or were self-generated), responding to calls from venues, identifying and responding to individuals and groups and patrolling areas where there were hyper-local 'kick-out times'. The patrol concluded around 8 am.		
**Strands of the service**	**Year 1**	**Year 2**
Welfare	316 checks	724 checks
Medical	161 individuals	97 individuals
Addressing anti-social behaviour, aggression, urination, pre-loading	365 incidents of violent or aggressive behaviour, 451 dispersals, 738 warnings about conduct	784 incidents of violent or aggressive behaviour, 675 dispersals, 1,235 warnings about conduct
Support to the licensed trade	2,295 liaisons with licensed trade; 226 responses to calls	2,482 liaisons with licensed trade; 125 responses to calls
Intelligence gathering	620,459 words	620,292 words

Sources: Fieldnotes; Community Safety Company, LNL Year 1 and Year 2 Reports

A key component of the new patrol service, which significantly increased from Year 1 to Year 2, was engagement with users of the NTE to ensure their welfare and to intervene early in anti-social behaviour, disturbance and nuisance to prevent its escalation:

> *So not only are they there to deal with the response side of it, but it's to try and prevent that happening in the first place, so to deal with those people who potentially would go on and commit further offences because they've started shouting and swearing and causing problems with someone up this end of the street. By the time you get down the other end, they've stopped in five other pubs on the other way, not been challenged, not been highlighted to anybody on the way down, although their behaviour's getting more and more rowdy. Then they go in, have a fight or cause a disturbance and need for police action further down the road.* (Interview, Police officer)

The welfare aspect of the service, which included community-safety officers helping members of the public, was also considered a critical component of the service and as shown in Table 6, increased significantly from Year 1 to 2. The officers also provided medical care and the medical service represented an evolution of the service. While it was always within their remit to have a first-aid trained officer, they expanded this provision shortly after starting the service and purchased additional medical equipment. In addition to supporting members of the public, the medical side of

For peer review only - http://bmjopen.bmj.com/site/about/guidelines.xhtml

the service was seen as a low-cost mechanism to reduce the burden on the London Ambulance Service and NHS. In the first two years the service reported preventing or cancelling the dispatch of 54 and 57 ambulances, respectively, which they calculated as savings of £16,200 and £14,478.

The Council reported a 17% reduction in alcohol-related crime between midnight and 8 am and a 14.4% reduction in alcohol-associated violence compared to the previous 12 months, although they assumed that this was not all attributable to the levy. They also reported a large increase (29-30%) in calls to the police and anti-social behaviour line about alcohol-related incidents which they further argued justified the need for the levy funding (Council, LNL Year 1 Report). In Year 2 the Council reported a 21% reduction in alcohol-related crimes compared to the previous 12 months, and a 24% decrease in anti-social behaviour calls (Council, LNL Year 2 Report).

Engagement with the licensed trade

Whilst ToC 1 emphasised the resources to police and manage NTE users in the area, the new patrol service also sought to develop relationships with local actors. Notably, they tried to develop relationships directly with the licensed trade – to monitor and support licensed operators to encourage safer business practices aimed at minimising anti-social behaviour within and outside the premises. In the first year of the levy funding, the patrol provided an introductory visit to 251 of the venues on the levy, which they argued was as an important mechanism to overcome hostility towards the levy and it's funded patrols.

Outside of the initial visits, the patrol worked to develop relationships and trust with venue staff through the repeated interactions; a key element of this, which they contrasted with the police, was the deployment of the same officers every night, particularly in the first year of the service:

> *One of the things you absolutely have when you're any form of policing, really, you've got to have that consistency. You've got to have the relationships. That comes from, you know, repetition. It's from meeting the DPSs [designated premise supervisors] on a regular basis, building up a trust and an understanding of what you're there to do […]. Well if you're on rotation you can't possibly know. You wouldn't even know who that person is and you certainly wouldn't be able to kind of build a balanced intelligence picture.* (Interview, Community safety officer)

When probed, community safety and police officers described an evolution of the relationships such that many licensees began to engage with the service, overcoming their initial resistance:

> *We came up initially against a lot of unhappiness because it's another tax effectively, a levy on these premises. They don't want to pay it. They're already paying ridiculously high rates and other business taxes and stuff. So, but, you know, I get that. But we're seeing a change now, you know. A year, 18 months down the road, they can see a*

For peer review only - http://bmjopen.bmj.com/site/about/guidelines.xhtml

> *benefit to it, so […], if they need help they'll get help. You know, they'll prevent stuff happening and hopefully make their business more attractive.* (Interview, Police officer)

Others licensees, however, remained what the officers referred to as 'hostile venues,' continuing to oppose the levy and its associated services. Officers put this down to a misunderstanding of the service's remit: "they [the licensees] see it as an enforcement role instead of a support role." (Excerpt from fieldnotes)

The community safety service was tasked with collecting intelligence to help the police or inform licensing decisions. Key to this intelligence-gather strategy was developing cooperative, rather than adversarial relationships with venue managers and staff, as described above. Information gathering and sharing amongst police, patrol and licensed venue operators was reciprocal, or in system terms, represented a positive feedback loop. Closer relationships amongst these three groups of actors appeared to emerge as a consequence, along with an 'othering' of certain venues who remained outside of this information sharing sub-system. Furthermore, the information gained was used to inform licensing decisions that affected alcohol availability (although as stated earlier, participants tended not discuss the LNL in terms of its impacts on alcohol availability). This intelligence was considered key to ensuring that managing and supporting the NTE was achieved across agencies and showed the ways in which new system actors developed and extended relationships with others to manage the local NTE:

> *And [the community safety officers] assist us as well. Not just us as licensing officers, but the police on the whole, because within our briefings we can say to them, just little things that have happened, that you wouldn't normally get a chance to deal with, can you go and check on this and this, this, this, and just have a look and even in terms of where new applications are coming in and people are asking to do various different things in their licence, and we're thinking, not sure you could do that, but we need to check the place out. […]. And they report back to us, and then that assists us in saying whether someone can or can't have a licence. It's invaluable, really."* (Interview, Police licensing officer)

Through the mechanisms described above, the Council, the police and the community safety officers reported that more venues were operating in a 'responsible manner' following the implementation of the levy. Hence, while the initial theory of change around extra resourcing focused on policing and management of NTE-users, by the second year of LNL's implementation a new mechanism for impact had emerged through information sharing and relationship building between NTE operators and the agents that patrolled and policed the NTE:

> Interviewer: do you think it (LNL and other licensing policies) has changed kind of how people consume alcohol in the borough?
> Respondent (Police licensing officer): *I don't think it's changed how people consume their alcohol in the borough. I think it's changed how operators operate.*

For peer review only - http://bmjopen.bmj.com/site/about/guidelines.xhtml

Taken holistically, the new service was perceived to have changed how actors within the system behaved and interacted with one another, disrupting previous patterns of behaviour as system elements responded and adapted to the new services. The service could also be conceptualised as having a non-linear effect on the broader system: the four-person patrol represented a fairly small system input which had the ability to affect, for example, perceptions of safety, emergency service usage, anti-social behaviour and alcohol retailing practices.

ToC 2: Reduced support for PPP schemes

The second key theory of change was that if businesses were liable to pay the levy, they would no longer support PPP schemes, particularly the local BID (Figure 3). This initial ToC did not accurately theorise how the system would adapt in the first two years of the intervention. Instead, in October 2016 members of the BID "again voted resoundingly for us to continue" (BID website) and the BID expanded to cover a larger geographical area. Following the introduction of the levy, the BID reported a key priority for safety in their area was: "achieving 24-hour security at [BID area] through co-ordinated working with street patrol [LNL-funded service]" (BID Annual Report, 2015/2016) and a licensee described a reliance on both BID- and LNL-funded patrols:

> *The night time economy is a major contributor to the wealth of the [BID area]. Making sure the environment is fun yet safe is a huge undertaking, not only for us licensees but also for the police and [LA] Council. [BID name] makes sure we are all working together. Not only do we have the [BID-funded] Police Team at our disposal but can also rely on [LNL-funded service]. (BID Annual Report, 2016/17, Bar Owner)*

Prior to the levy's implementation, members of the licensed trade argued that the BID-funded services addressed their policing and safety needs. However, as the LNL-funded community safety patrol became embedded in the local system, some members of the BID came to see the community safety patrols as a complement to their own funded services and promoted collaboration between the two services, leading to greater resources for managing the local environment.

ToC 3 and 4: Premises will (3) vary hours or (4) close due to unwillingness or inability to pay the Levy

The final theories of change were that a large number of premises would vary their hours in response to the introduction of the LNL, or in some cases close completely, which would lead to unanticipated consequences (Figure 4). The data reported by the Council showed that approximately one quarter of all premises who were initially liable to pay the levy either varied their licence hours

For peer review only - http://bmjopen.bmj.com/site/about/guidelines.xhtml

or closed prior to the implementation date, which was lower than the 42% who indicated they would during the consultation period. The majority of these businesses varied their hours, rather than permanently closing their doors.

The majority of premises that were identified as being liable to pay the levy continued to operate after midnight and the LA did not see a re-introduction of a 'defacto terminal hour'. However, there remained clusters of bars and pubs that closed at similar time, which the community safety officers would refer to informally as 'kick-out times'.

Members of the licensed trade and some residents and visitors theorised the levy would create an NTE that lacked diversity, which in turn would drive down visitor numbers. During the course of fieldwork, we observed a busy NTE with bustling streets and busy venues. All the users of the NTE we spoke with during the course of fieldwork in the second year of the levy described numerous and diverse places to go out in the LA:

> *[Name] was talking about how there used to be only one place really to go (The Name – which she says is a great pub), but now there are so many options. The places to go out don't just include alcohol: "It used to be that there were just three places to eat ... [she lists their names] and now there are so many to choose from.* (Excerpt from fieldnotes)

Cumulatively, these data show that some premises did vary their hours in response to the introduction of the levy, but the levy remained viable and that an insufficient number of premises closed at midnight to re-introduce a 'terminal hour'. The LA maintained a reputation for providing a diverse and busy NTE following the implementation of the intervention.

DISCUSSION AND CONCLUSION

This two-phased process evaluation sought to describe the local system into which the LNL is introduced and explore how the intervention may lead to changes as the system, its actors and the intervention adapt and co-evolve over time. We identified four main theories of change that describe the multiple processes by which the intervention might generate system change, including those that were unanticipated at the intervention design stage.

The first theory of change articulated how the intervention was designed to increase the resources available to police and manage the NTE. The evaluation shed light on the mechanisms by which those resources might lead to change; specifically, by introducing new actors into the local system, who through consistent, visible and prolonged relationship building with the licensed trade and the public, sought to disrupt local system rules and develop new practices. Findings from the first two years of the levy suggest that these efforts led to an evolution in the way that many, although not

For peer review only - http://bmjopen.bmj.com/site/about/guidelines.xhtml

For peer review only

all, licensees viewed the levy and a change in how some venues are managed. Information sharing practices provided emergent opportunities for mutually-supportive relationships between those who operate licensed premises and those who manage or police the NTE – although some premises were 'othered': i.e. positioned outside of these relationships.

Contrary to expectations, the introduction of the LNL did not undermine PPP schemes during the study period, particularly the BID, as expressed in the second theory of change. The reason that the LNL's implementation co-occurred with an increase in voluntary industry initiatives and partnerships is unclear. Commercial actors had originally showed a clear preference for voluntary and partnership initiatives (such as the BID) over regulation (such as LNL), and there may have been a desire amongst commercial interests to maintain support and claim success for local PPP schemes during a period when the LA was adopting discretionary regulatory powers – in order to avoid regulation themselves. Whatever their reasons, some commercial actors appeared to believe that both regulatory and voluntary activities had adapted to complement each other and confer their own benefits on the NTE. The mechanisms by which increased regulation of the sale of harmful commodities might lead to increased voluntary and partnership activity warrants further investigation across different types of interventions and harmful commodities.

With regards to the third and fourth theories of change, there was some evidence that premises varied their hours in response to the levy, but these changes did not ultimately undermine the viability of the levy, lead to the re-introduction of a terminal hour, or obviously damage the NTE's reputation as being diverse and vibrant. Taken together, those in charge of developing and implementing the levy at the local level, viewed these early indications of system change as successful. This suggests a reinforcing feedback loop, whereby the perceived success of the levy ensured its continuation.

Almost all actors in the system deprioritised discourses of reduced alcohol harm through reduced availability – even though we know that some degree of temporal (earlier 'kick out' times) and physical (premise closures) reductions in availability did occur. The discourse around LNL's impacts on harm focused almost exclusively on secondary prevention of harms through improved community safety services and policing of anti-social behaviour linked to intoxicated NTE users. Discourses around primary prevention (reducing or preventing alcohol consumption) were deprioritised in favour of economic and cultural impacts. Although these findings focus on the local system, they occur within a wider system in which industry actors frame discourse and lobby policymakers to encourage voluntary partnership approaches to regulation (42). The focus on secondary, rather than primary, prevention suits commercial interests in that action to prevent harm

For peer review only - http://bmjopen.bmj.com/site/about/guidelines.xhtml

is taken after the point of sale. The nature of the framing of harm prevention, and when preventive action should be taken, is a point that could be explored through further research.

This evaluation represents the first application of our complex systems framework for process evaluations (32). While many in public health have argued that complex system approaches can produce better evidence for decision making that account for real-world complexities (13), there have been relatively few prominent examples of this perspective applied to public health process evaluation to date (43). In addition, many process evaluations in this field have focused largely on describing static systems with limited attention paid to dynamic processes of system change (32). This work attempts to address some of these limitations. The use of the framework and explicit application of systems and complexity concepts was utilised to make sense of the broader system into which the levy is introduced, the many processes through which the levy may lead to impacts, many of which might be unanticipated, and the dynamic responses to the intervention that lead to an evolution of the system's actors, their relationships with each other, the intervention, and the system as a whole.

This process evaluation is also the first known evaluation of the LNL (44,45). An Institute of Alcohol Studies (IAS) report reviewed the impact of the Licensing Act (2003) ten-years post-implementation and reported that the LNL had the potential to reduce alcohol availability by encouraging premises to shorten their opening hours, could help foster a cleaner environment through the provision of additional street cleaning resources and could be used to promote diversity in the NTE. The report also highlighted other possible impacts of the levy, including that the levy might prevent or damage partnership working between LAs and the alcohol industry, impact the industry's profitability, and be too inflexible a tool to be well suited to many LA's NTEs (44). The findings from our process evaluation shed light on the mechanism by which these impacts may or may not occur within a local system. Despite acknowledging that there has been no evaluation of the LNL's impact on crime and disorder, a subsequent joint IAS and Foundation for Alcohol Research and Education report argues: "Attempts have been made to limit closing times in areas with acute problems, through the late night levy and early morning restriction order, although these policies have also proven largely ineffective" (45 p.10). Few LAs have tried to implement the LNL, so in that sense the impacts of this discretionary intervention have been highly localised. However, in light of the findings from this process evaluation that suggests some of the mechanisms by which the LNL may reduce crime and disorder, as well as other problems in the NTE, we would recommend further evaluation of the LNL, and of alcohol and health impacts within local systems.

For peer review only - http://bmjopen.bmj.com/site/about/guidelines.xhtml

Strengths and limitations

As evaluators, we made two crucial boundary decisions in this process evaluation: to focus on the local level and to include and exclude certain local system variables from our analysis. Together, these represent an emphasis on horizontal complexity. The first decision was made a-priori and was influenced by the nature of the intervention (i.e. a locally-delivered intervention) and our interest in the delivery processes within one LA system. However, there are also vertical complexities that affect, influence and interact with the local system; the local system is embedded within broader regional, national and international systems and the boundaries between them are open (15). We included some consideration of the national system in order to make sense of the context in which the LNL was introduced as a discretionary power available to LAs, but other stakeholders within the national and local systems, or other evaluation teams might have chosen to broaden their boundaries. Given limited evaluation resources, there may be a trade-off of breadth versus depth. Some evaluators have suggested conducting Qualitative Comparison Analyses (QCAs) to address this challenge (46,47). In addition, as evaluators, we made decisions about the variables of interest within the local system (46). This was informed by the data generated through the evaluation and our aim was to focus on the variables we found to be most relevant to the LNL. Examples of this exist within the systems literature, for example, with researchers utilising data generated through documentary review and interviews to develop causal loop diagrams (48). However, this raises important considerations around power dynamics and who ultimately decides where boundaries are drawn (49). This work could fruitfully be extended by engaging in processes that invite system stakeholders to participate in the boundary decisions and critique (20,50).

A limitation of this evaluation is that we did not collect primary data from residents or those working within the healthcare system. In addition, no data were generated or analysed about the broader economic impacts on the local economy. Conducting systems research often involves collecting data from a wide range of different actors across a given system (16), which is resource-intensive and challenging when conducting smaller, local evaluations. Conducting a documentary analysis is one possible way to include data from a wider range of participants than might be possible through interviews and observations alone. In this evaluation, for example, we collected limited primary data from members of the licensed trade and relied on their extensive consultation responses which provided insight into the ways in which they theorised the levy might lead to a range of unanticipated impacts across the local system.

We collected data for Phases 1 and 2 concurrently in the post-implementation period (although many documents included in our documentary review were produced prior to the levy's implementation). As a result, Phase 1 informed the Phase 2 analysis, but not the Phase 2 data

For peer review only - http://bmjopen.bmj.com/site/about/guidelines.xhtml

collection. While this approach underscores the flexibility of the process evaluation from a complex systems perspective framework, it also limited our ability to follow all emergent findings. For example, the consultation responses underscored how the levy might affect employment patterns in the local area, with premises having to vary their employees' shift patterns or make some employees redundant. We did not collect data from premises or from the LA that could then speak to these possible impacts.

Future direction and conclusions

The process evaluation from a complex systems perspective is intended to be adaptive, drawing on early findings to inform subsequent data collection and analysis. A logical next step for this evaluative process is to measure alcohol-related outcomes and to understand the processes beyond the immediate local system of interest, to consider the vertical dimensions of complexity. The evaluation shed light on the possible spillover effects to neighbouring local authorities, and these processes and outcomes could be explored. In addition, in the Modern Crime Prevention Strategy (2016) the Government proposed changing the structure of the levy to apply to specific types of premises or specific hotspots within the NTE, rather than entire LAs. At the end of the same year, the Home Office concluded that the levy had been implemented in fewer LAs that anticipated (n=7) because of criticisms "that LAs consider the levy to be inflexible and the licensed trade has highlighted issues of unfairness in terms of which businesses pay the levy" (51 p.1). While the changes were to come into effect in 2020, by which 11 LAs had a LNL (52,53), at the time of writing, they have yet to do so. Finally, the Covid-19 pandemic represents a large system shock that has had significant financial impacts on LAs and the licensed trade (54). This process evaluation could usefully be extended to explore these interacting local, regional, national and international processes and systems.

Figure 1 legend:
Orange bubbles = national variables; Yellow bubbles = local variables; Green bubbles = immediate theorised impacts stemming from levy introduction
Solid line: positive relationship between variables; Dashed line: inverse relationship between variables; Dotted green line: theorised impacts stemming from the levy introduction

Figure 2 legend:
Orange bubble = national variable; Yellow bubbles = local variables; Green bubble = immediate theorised impact stemming from levy introduction
Solid line: positive relationship between variables; Dashed line: inverse relationship between variables; Dotted green line: theorised impact stemming from the levy introduction

Figure 3 legend:
Orange bubble = national variable; Yellow bubbles = local variables; Green bubbles = immediate theorised impacts stemming from levy introduction

For peer review only - http://bmjopen.bmj.com/site/about/guidelines.xhtml

For peer review only

Solid line: positive relationship between variables; Dashed line: inverse relationship between variables; Dotted green lines: theorised impacts stemming from the levy introduction

Figure 4 legend:
Yellow bubbles = local variables; Green bubbles = immediate theorised impacts stemming from levy introduction
Solid line: positive relationship between variables; Dashed line: inverse relationship between variables; Dotted green lines: theorised impacts stemming from the levy introduction

Funding statement: This study was funded by the National Institute for Health Research (NIHR) School for Public Health Research (SPHR), Grant Reference Number PD-SPH-2015. The views expressed are those of the author(s), and not necessarily those of the NIHR or the Department of Health and Social Care.

Competing interests statement: DM is a joint Public Health Strategist/Associate Professor covering the intervention area, but was not involved in the design or delivery of the intervention. The authors declare no other competing interests.

Author contributions: EM designed the methodology, conducted data collection, led on the analysis and drafted this manuscript. ME contributed to the analysis. EM, DM, MP and ME provided input into data interpretation, critically revised the manuscript and approved the final version.

Acknowledgments: We gratefully acknowledge discussions with Dr David Humphreys and Professor Niamh Fitzgerald that helped us during data interpretation.

Data availability statement: No additional data are available. The ethical approval granted for this study stipulated that interview transcripts would not be shared beyond the research team.

For peer review only

REFERENCES

1. Burton R, Henn C, Lavoie D, O'Connor R, Perkins C, Sweeney K, et al. A rapid evidence review of the effectiveness and cost-effectiveness of alcohol control policies: an English perspective. Lancet. 2017; 389(10078):1558-80. https://doi.org/10.1016/S0140-6736(16)32420-5.

2. Griswold MG, Fullman N, Hawley C, Arian N, Zimsen SRM, Tymeson HD, et al. Alcohol use and burden for 195 countries and territories, 1990–2016: a systematic analysis for the Global Burden of Disease Study 2016. Lancet. 2018; 392(10152):1015-35. https://doi.org/10.1016/S0140-6736(18)31310-2.

3. Rehm J, Baliunas D, Borges GL, Graham K, Irving H, Kehoe T, et al. The relation between different dimensions of alcohol consumption and burden of disease: an overview. Addiction. 2010; 105(5):817-43. https://doi.org/10.1111/j.1360-0443.2010.02899.x.

4. Philpot R, Liebst LS, Møller KK, Lindegaard MR, Levine M. Capturing violence in the night-time economy: a review of established and emerging methodologies. Aggress Violent Behav. 2019; 46:56-65. https://doi.org/10.1016/j.avb.2019.02.004.

5. Hadfield P, Lister S, Traynor P. 'This town'sa different town today': policing and regulating the night-time economy. Criminology and Criminal Justice. 2009; 9(4):465-85. https://doi.org/10.1177/1748895809343409.

6. Roberts M. From 'creative city'to 'no-go areas' - the expansion of the night-time economy in British town and city centres. Cities. 2006; 23(5):331-8. https://doi.org/10.1016/j.cities.2006.05.001.

7. Hayward K, Hobbs D. Beyond the binge in 'booze Britain': market-led liminalization and the spectacle of binge drinking. Br J Sociol. 2007; 58(3):437-56. https://doi.org/10.1111/j.1468-4446.2007.00159.x.

8. Nicholls J. Time for reform? Alcohol policy and cultural change in England since 2000. British Politics. 2012; 7(3):250-71. https://doi.org/10.1057/bp.2012.7.

9. Nicholls J. Public health and alcohol licensing in the UK: challenges, opportunities, and implications for policy and practice. Contemp Drug Probl. 2015; 42(2):87-105. https://doi.org/10.1177/0091450915579875.

10. Martineau F, Graff H, Mitchell C, Lock K. Responsibility without legal authority? Tackling alcohol-related health harms through licensing and planning policy in local government. J Public Health. 2014; 36(3):435-42. https://doi.org/10.1093/pubmed/fdt079.

11. Carey G, Malbon E, Carey N, Joyce A, Crammond B, Carey A. Systems science and systems thinking for public health: a systematic review of the field. BMJ Open. 2015; 5(12):e009002. http://dx.doi.org/10.1136/bmjopen-2015-009002.

12. Bagnall A-M, Radley D, Jones R, Gately P, Nobles J, Van Dijk M, et al. Whole systems approaches to obesity and other complex public health challenges: a systematic review. BMC Public Health. 2019; 19(1):8. https://doi.org/10.1186/s12889-018-6274-z.

13. Rutter H, Savona N, Glonti K, Bibby J, Cummins S, Finegood DT, et al. The need for a complex systems model of evidence for public health. Lancet. 2017; 390(10112):2602-4. https://doi.org/10.1016/S0140-6736(17)31267-9.

For peer review only - http://bmjopen.bmj.com/site/about/guidelines.xhtml

14. Meadows DH, Wright D. Thinking in Systems: A Primer. White River Junction, Vermont: Chelsea Green Publishing; 2008.

15. Cilliers P. Boundaries, hierarchies and networks in complex systems. International Journal of Innovation Management. 2001; 5(02):135-47. https://doi.org/10.1142/S1363919601000312.

16. Finegood DT, Johnston LM, Steinberg M, Matteson CL, Deck P. Complexity, systems thinking and health behavior change. In: Kahan S, Green L, Gielen A, Fagen P, editors. Health behavior change in populations. Baltimore, MD: Johns Hopkins University Press; 2014. pp. 435-58.

17. Boulton J, Allen P, Bowman C. Embracing Complexity: Strategic Perspectives for an Age of Turbulence. Oxford: Oxford University Press; 2015.

18. Walton M. Setting the context for using complexity theory in evaluation: boundaries, governance and utilisation. Evid Policy. 2016; 12(1):73-89. https://doi.org/10.1332/174426415X14298726247211.

19. Hawe P, Bond L, Butler H. Knowledge theories can inform evaluation practice: what can a complexity lens add? New Dir Eval. 2009; 2009(124):89-100. https://doi.org/10.1002/ev.316.

20. Walton M. Applying complexity theory: a review to inform evaluation design. Eval Program Plann. 2014; 45:119-26. https://doi.org/10.1016/j.evalprogplan.2014.04.002.

21. Rickles D, Hawe P, Shiell A. A simple guide to chaos and complexity. J Epidemiol Community Health. 2007; 61(11):933-7. http://dx.doi.org/10.1136/jech.2006.054254.

22. Buijs J-M, Eshuis J, Byrne D. Approaches to researching complexity in public management. In: Teisman G, van Buuren A, Gerrits L, editors. Managing complex governance systems: dynamics, self-organization and coevolution in public investments. London: Routledge; 2009. pp. 37-55.

23. Eppel E, Matheson A, Walton M. Applying complexity theory to New Zealand public policy: principles for practice. Policy Quarterly. 2011; 7(1):48-55. https://doi.org/10.26686/pq.v7i1.4369.

24. Kania A, Patel AB, Roy A, Yelland GS, Verhoef MJ. Capturing the complexity of evaluations of health promotion interventions: a scoping review. Can J Program Eval. 2012; 27(1):65-91.

25. Byrne D, Callaghan G. Complexity Theory and the Social Sciences: The State of the Art. Abingdon: Routledge; 2014.

26. Gates EF. Making sense of the emerging conversation in evaluation about systems thinking and complexity science. Eval Program Plann. 2016; 59:62-73. https://doi.org/10.1016/j.evalprogplan.2016.08.004.

27. Walton M. Expert views on applying complexity theory in evaluation: opportunities and barriers. Evaluation. 2016; 22(4):410-23. https://doi.org/10.1177/1356389016667890.

28. Gatrell AC. Complexity theory and geographies of health: a critical assessment. Soc Sci Med. 2005; 60(12):2661-71. https://doi.org/10.1016/j.socscimed.2004.11.002.

29. Bambra C, Smith KE, Pearce J. Scaling up: the politics of health and place. Soc Sci Med. 2019; 232:36-42. https://doi.org/10.1016/j.socscimed.2019.04.036.

For peer review only - http://bmjopen.bmj.com/site/about/guidelines.xhtml

30. McGill E, Petticrew M, Marks D, McGrath M, Rinaldi C, Egan M. Applying a complex systems perspective to alcohol consumption and the prevention of alcohol-related harms in the 21st century: a scoping review. Addiction. 2020; https://doi.org/10.1111/add.15341.

31. Egan M, McGill E, Penney T, Anderson de Cuevas R, Er V, Orton L, et al. NIHR SPHR Guidance on systems approaches to local public health evaluation. Part 1: Introducing systems thinking. London: National Institute for Health Research School for Public Health Research. 2019 [Accessed 2021 Feb 16]. Available from: https://sphr.nihr.ac.uk/wp-content/uploads/2018/08/NIHR-SPHR-SYSTEM-GUIDANCE-PART-1-FINAL_SBnavy.pdf.

32. McGill E, Marks D, Er V, Penney T, Petticrew M, Egan M. Qualitative process evaluation from a complex systems perspective: a systematic review and framework for public health evaluators. PLoS Med. 2020; 17(11):e1003368. https://doi.org/10.1371/journal.pmed.1003368.

33. Makleff S, Billowitz M, Garduño J, Cruz M, Silva Márquez VI, Marston C. Applying a complex adaptive systems approach to the evaluation of a school-based intervention for intimate partner violence prevention in Mexico. Health Policy Plan. 2020; 35(8):993-1002. https://doi.org/10.1093/heapol/czaa067.

34. Mintrop R, Pryor L, Ordenes M. A complex adaptive system approach to evaluation: application to a pay-for-performance program in the USA. Educ Assess Eval Account. 2018; 30(3):285-312. https://doi.org/10.1007/s11092-018-9276-6.

35. Hawe P, Shiell A, Riley T. Theorising interventions as events in systems. Am J Comm Psych. 2009; 43(3-4):267-76. https://doi.org/10.1007/s10464-009-9229-9.

36. Moore GF, Audrey S, Barker M, Bond L, Bonell C, Hardeman W, et al. Process evaluation of complex interventions: Medical Research Council guidance. BMJ. 2015; 350:h1258. https://doi.org/10.1136/bmj.h1258.

37. Home Office. Police Reform and Social Responsibility Act 2011: The Late Night Levy. London: Home Office. 2011 [Accessed 2021 Feb 16]. Available from: https://assets.publishing.service.gov.uk/government/uploads/system/uploads/attachment_data/file/98126/late-night-levy.pdf.

38. Home Office. Impact assessment for the alcohol measures in the Police Reform and Social Responsbility Act. London: Home Office. 2011 [Accessed 2021 Feb 16]. Available from: https://www.legislation.gov.uk/ukia/2011/549/pdfs/ukia_20110549_en.pdf.

39. Home Office. Amended guidance on the Late Night Levy. London: Home Office. 2015 [Accessed 16 Feb 2021]. Available from: https://assets.publishing.service.gov.uk/government/uploads/system/uploads/attachment_data/file/416092/Late_Night_Levy_-_new_guidance_as_at_24_March_2015__final_.pdf.

40. Visual Understanding Environment (VUE). Tufts University. Medford, MA. Available at: https://vue.tufts.edu/.

41. NVivo Qualitative Data Analysis Software, Version 12. QSR International Pty Ltd.

For peer review only

42. Hawkins B, Holden C. Framing the alcohol policy debate: industry actors and the regulation of the UK beverage alcohol market. Crit Policy Stud. 2013; 7(1):53-71. https://doi.org/10.1080/19460171.2013.766023.

43. McGill E, Er V, Penney T, Egan M, White M, Meier P, et al. Evaluation of public health interventions from a complex systems perspective: a research methods review. Soc Sci Med. 2021; 272:113697. https://doi.org/10.1016/j.socscimed.2021.113697.

44. Foster J, Charalambides L. The Licensing Act (2003): its uses and abuses 10 years on. London: Institute of Alcohol Studies. 2016 [Accessed 2021 Feb 16]. Available from: http://www.ias.org.uk/uploads/pdf/IAS%20reports/rp22032016.pdf.

45. Foster J, Harrison A, Brown K, Manton E, Wilkinson C, Ferguson A. Anytime, anyplace, anywhere? Addressing physical availability of alcohol in Australia and the UK. London and Canberra: Institute for Alcohol Studies and the Foundation for Alcohol Research and Education. 2017 [Accessed 2021 Feb 16]. Available from: https://fare.org.au/wp-content/uploads/AVAILABILITY-REPORT-30-May-2017-FINAL.pdf.

46. Verweij S, Gerrits LM. Understanding and researching complexity with qualitative comparative analysis: evaluating transportation infrastructure projects. Evaluation. 2013; 19(1):40-55. https://doi.org/10.1177/1356389012470682.

47. Hudson J, Kühner S. Qualitative comparative analysis and applied public policy analysis: new applications of innovative methods. Policy and Society. 2013; 32(4):279-87. https://doi.org/10.1016/j.polsoc.2013.10.001.

48. Baugh Littlejohns L, Baum F, Lawless A, Freeman T. The value of a causal loop diagram in exploring the complex interplay of factors that influence health promotion in a multisectoral health system in Australia. Health Res Policy Syst. 2018; 16(1):126. https://doi.org/10.1186/s12961-018-0394-x.

49. Mowles C. Complex, but not quite complex enough: the turn to the complexity sciences in evaluation scholarship. Evaluation. 2014; 20(2):160-75. https://doi.org/10.1177/1356389014527885.

50. Ulrich W, Reynolds M. Critical systems heuristics: the idea and practice of boundary critique. In: Reynolds M, Holwell S, editors. Systems approaches to making change: a practical guide. London: Springer; 2020. pp. 255-306.

51. Home Office. Policing and Crime Bill: changes to the Late Night Levy impact assessment. London: Home Office. 2016 [Accessed 2021 Feb 16]. Available from: https://assets.publishing.service.gov.uk/government/uploads/system/uploads/attachment_data/file/557110/16_signed_IA_late_night_levy.pdf.

52. Woodhouse J. The late night levy. London: House of Commons Library. 2019 [Accessed 2021 Feb 16]. Available from: https://commonslibrary.parliament.uk/research-briefings/sn07100/.

53. Southwark Council. Late Night Levy London: Southwark Council 2019. [Accessed 2021 Feb 16]. Available from: https://www.southwark.gov.uk/business/licences/business-premises-licensing/alcohol-late-night-refreshment-and-entertainment-licences/late-night-levy.

For peer review only - http://bmjopen.bmj.com/site/about/guidelines.xhtml

54. Kolvin P, Eames C. Late night levy in lockdown. Morning Advertiser. 2020 Apr 29. [Accessed 2021 Feb 16]. Available from: https://www.morningadvertiser.co.uk/Article/2020/04/29/Why-do-I-have-to-pay-the-late-night-levy-in-lockdown.

For peer review only

For peer review only - http://bmjopen.bmj.com/site/about/guidelines.xhtml

Figure 1: System map

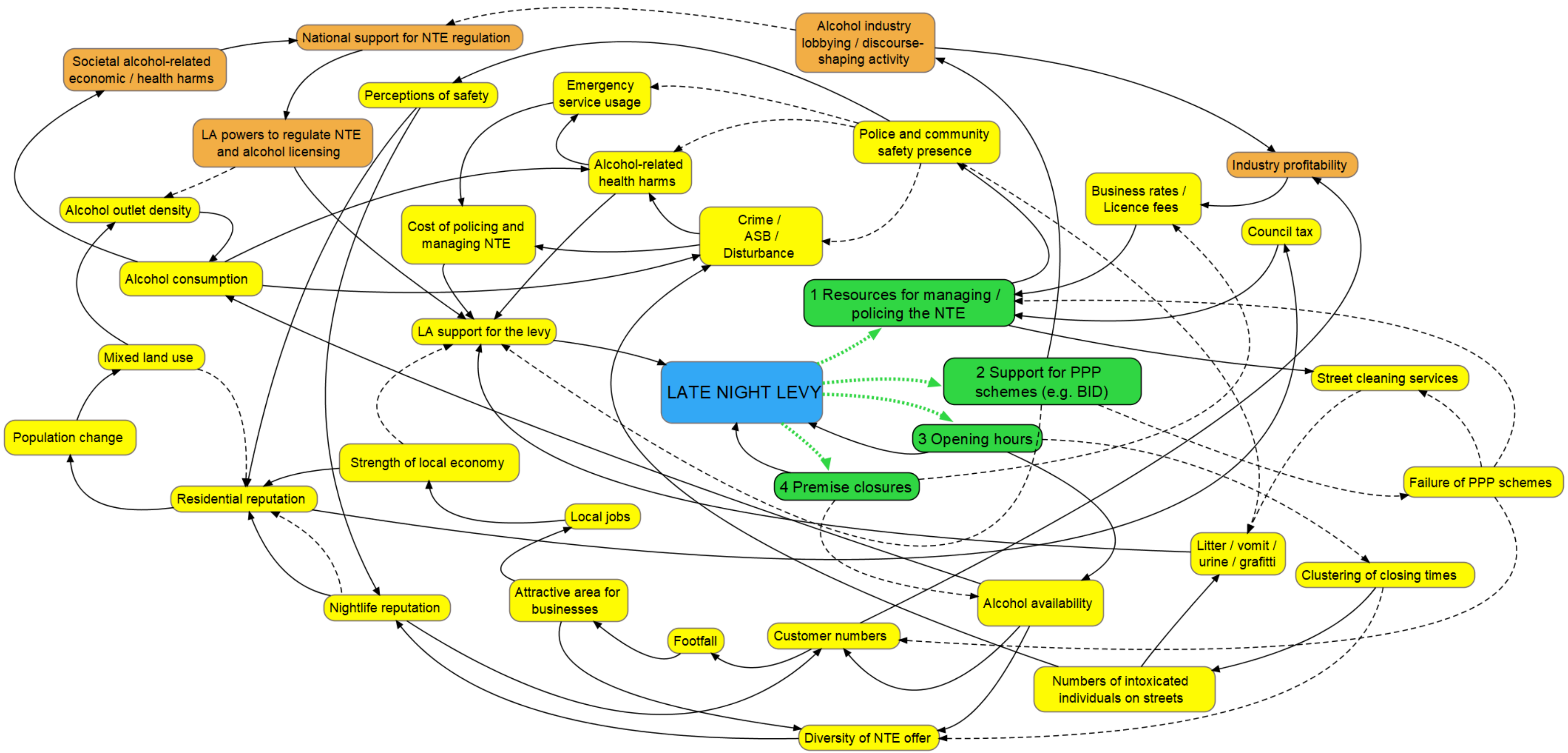

Figure 2: Theory of change 1

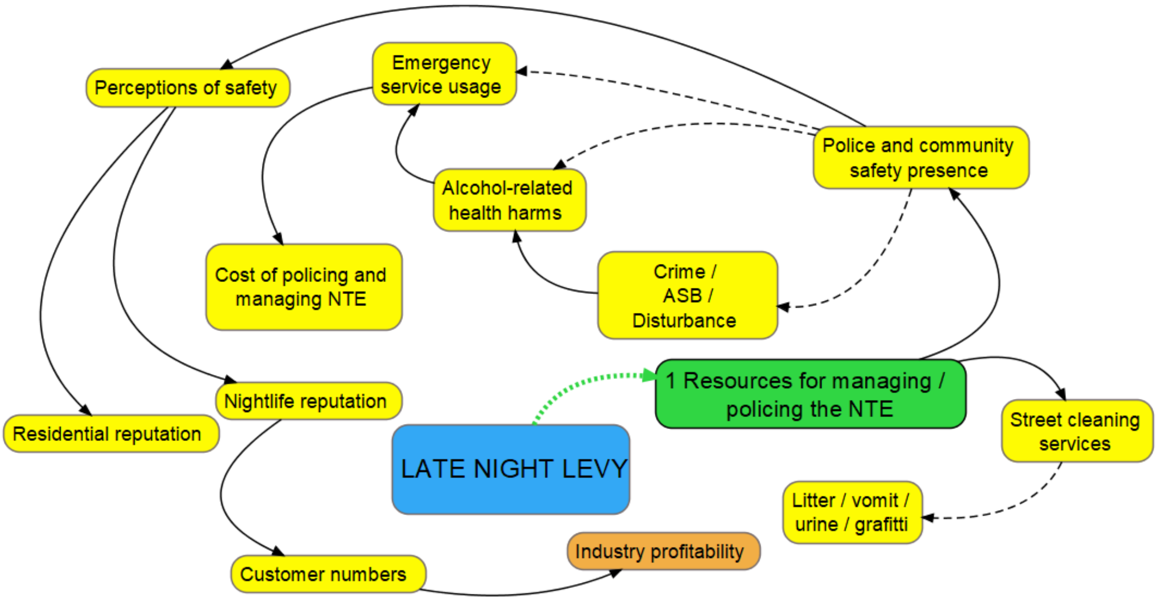

Figure 3: Theory of change 2

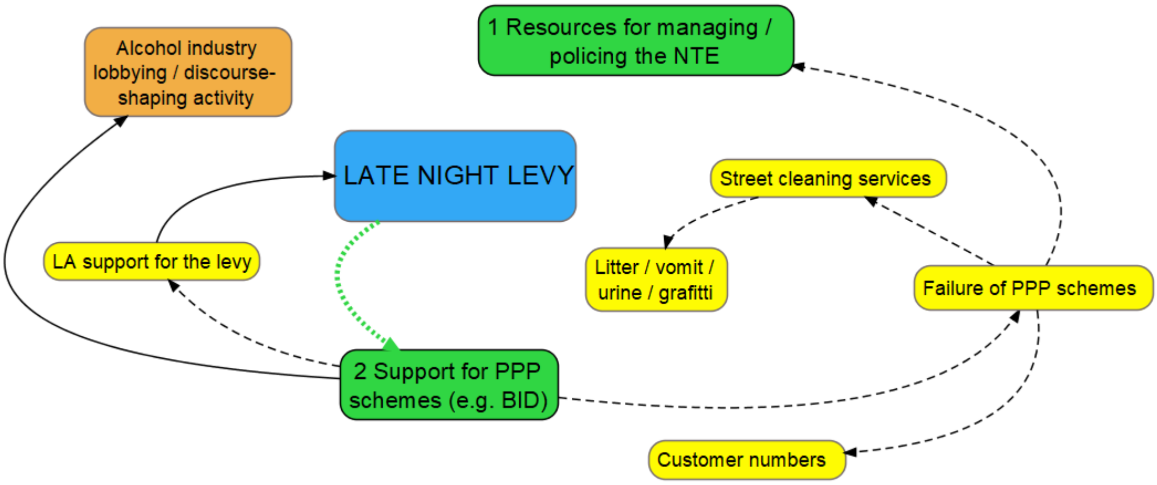

For peer review only

Figure 4: Theories of change 3 and 4

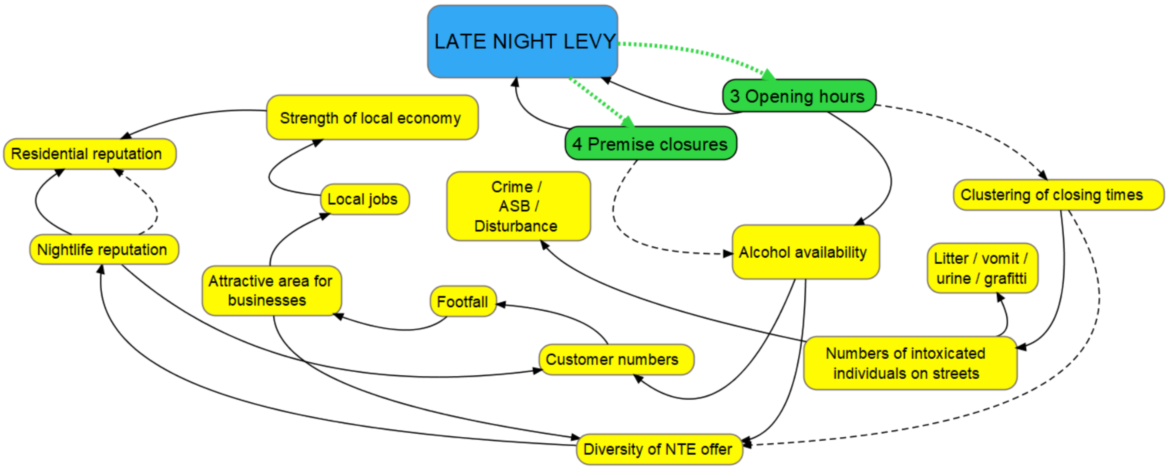

For peer review only

Standards for Reporting Qualitative Research (SRQR)*

http://www.equator-network.org/reporting-guidelines/srqr/

Title and abstract

	Page no(s).
**Title** - Concise description of the nature and topic of the study Identifying the study as qualitative or indicating the approach (e.g., ethnography, grounded theory) or data collection methods (e.g., interview, focus group) is recommended	P.1
**Abstract** - Summary of key elements of the study using the abstract format of the intended publication; typically includes background, purpose, methods, results, and conclusions	P.2

Introduction

**Problem formulation** - Description and significance of the problem/phenomenon studied; review of relevant theory and empirical work; problem statement	P.4-6
**Purpose or research question** - Purpose of the study and specific objectives or questions	P.6

Methods

**Qualitative approach and research paradigm** - Qualitative approach (e.g., ethnography, grounded theory, case study, phenomenology, narrative research) and guiding theory if appropriate; identifying the research paradigm (e.g., postpositivist, constructivist/ interpretivist) is also recommended; rationale**	P.6
**Researcher characteristics and reflexivity** - Researchers' characteristics that may influence the research, including personal attributes, qualifications/experience, relationship with participants, assumptions, and/or presuppositions; potential or actual interaction between researchers' characteristics and the research questions, approach, methods, results, and/or transferability	P.8
**Context** - Setting/site and salient contextual factors; rationale**	P.6-7
**Sampling strategy** - How and why research participants, documents, or events were selected; criteria for deciding when no further sampling was necessary (e.g., sampling saturation); rationale**	P.8-10
**Ethical issues pertaining to human subjects** - Documentation of approval by an appropriate ethics review board and participant consent, or explanation for lack thereof; other confidentiality and data security issues	P.10
**Data collection methods** - Types of data collected; details of data collection procedures including (as appropriate) start and stop dates of data collection and analysis, iterative process, triangulation of sources/methods, and modification of procedures in response to evolving study findings; rationale**	P.8-12

For peer review only - http://bmjopen.bmj.com/site/about/guidelines.xhtml

For peer review only

**Data collection instruments and technologies** - Description of instruments (e.g., interview guides, questionnaires) and devices (e.g., audio recorders) used for data collection; if/how the instrument(s) changed over the course of the study	P.9
**Units of study** - Number and relevant characteristics of participants, documents, or events included in the study; level of participation (could be reported in results)	P.8-9
**Data processing** - Methods for processing data prior to and during analysis, including transcription, data entry, data management and security, verification of data integrity, data coding, and anonymization/de-identification of excerpts	P.10
**Data analysis** - Process by which inferences, themes, etc., were identified and developed, including the researchers involved in data analysis; usually references a specific paradigm or approach; rationale**	P.11-12
**Techniques to enhance trustworthiness** - Techniques to enhance trustworthiness and credibility of data analysis (e.g., member checking, audit trail, triangulation); rationale**	P.11

Results/findings

**Synthesis and interpretation** - Main findings (e.g., interpretations, inferences, and themes); might include development of a theory or model, or integration with prior research or theory	P. 12-30
**Links to empirical data** - Evidence (e.g., quotes, field notes, text excerpts, photographs) to substantiate analytic findings	P.13-30

Discussion

**Integration with prior work, implications, transferability, and contribution(s) to the field** - Short summary of main findings; explanation of how findings and conclusions connect to, support, elaborate on, or challenge conclusions of earlier scholarship; discussion of scope of application/generalizability; identification of unique contribution(s) to scholarship in a discipline or field	P.30-32
**Limitations** - Trustworthiness and limitations of findings	P.32-33

Other

**Conflicts of interest** - Potential sources of influence or perceived influence on study conduct and conclusions; how these were managed	P.34
**Funding** - Sources of funding and other support; role of funders in data collection, interpretation, and reporting	P.34

*The authors created the SRQR by searching the literature to identify guidelines, reporting standards, and critical appraisal criteria for qualitative research; reviewing the reference lists of retrieved sources; and contacting experts to gain feedback. The SRQR aims to improve the transparency of all aspects of qualitative research by providing clear standards for reporting qualitative research.

For peer review only

For peer review only - http://bmjopen.bmj.com/site/about/guidelines.xhtml

**The rationale should briefly discuss the justification for choosing that theory, approach, method, or technique rather than other options available, the assumptions and limitations implicit in those choices, and how those choices influence study conclusions and transferability. As appropriate, the rationale for several items might be discussed together.

Reference:
O'Brien BC, Harris IB, Beckman TJ, Reed DA, Cook DA. **Standards for reporting qualitative research: a synthesis of recommendations.** *Academic Medicine*, Vol. 89, No. 9 / Sept 2014 DOI: 10.1097/ACM.0000000000000388

For peer review only

For peer review only - http://bmjopen.bmj.com/site/about/guidelines.xhtml

BMJ Open

Addressing alcohol-related harms in the local night-time economy: a qualitative process evaluation from a complex systems perspective

Journal:	*BMJ Open*
Manuscript ID	bmjopen-2021-050913.R1
Article Type:	Original research
Date Submitted by the Author:	09-Feb-2022
Complete List of Authors:	McGill, Elizabeth; London School of Hygiene & Tropical Medicine, Department of Health Services Research and Policy<br>Marks, Dalya; London School of Hygiene & Tropical Medicine, Department of Public Health, Environments and Society<br>Petticrew, Mark; London School of Hygiene & Tropical Medicine, Department of Public Health, Environments and Society<br>Egan, Matt; London School of Hygiene & Tropical Medicine, Department of Public Health, Environments and Society
**Primary Subject Heading**:	Public health
Secondary Subject Heading:	Qualitative research
Keywords:	PUBLIC HEALTH, QUALITATIVE RESEARCH, STATISTICS & RESEARCH METHODS

SCHOLARONE™
Manuscripts

I, the Submitting Author has the right to grant and does grant on behalf of all authors of the Work (as defined in the below author licence), an exclusive licence and/or a non-exclusive licence for contributions from authors who are: i) UK Crown employees; ii) where BMJ has agreed a CC-BY licence shall apply, and/or iii) in accordance with the terms applicable for US Federal Government officers or employees acting as part of their official duties; on a worldwide, perpetual, irrevocable, royalty-free basis to BMJ Publishing Group Ltd ("BMJ") its licensees and where the relevant Journal is co-owned by BMJ to the co-owners of the Journal, to publish the Work in this journal and any other BMJ products and to exploit all rights, as set out in our licence.

The Submitting Author accepts and understands that any supply made under these terms is made by BMJ to the Submitting Author unless you are acting as an employee on behalf of your employer or a postgraduate student of an affiliated institution which is paying any applicable article publishing charge ("APC") for Open Access articles. Where the Submitting Author wishes to make the Work available on an Open Access basis (and intends to pay the relevant APC), the terms of reuse of such Open Access shall be governed by a Creative Commons licence – details of these licences and which Creative Commons licence will apply to this Work are set out in our licence referred to above.

Other than as permitted in any relevant BMJ Author's Self Archiving Policies, I confirm this Work has not been accepted for publication elsewhere, is not being considered for publication elsewhere and does not duplicate material already published. I confirm all authors consent to publication of this Work and authorise the granting of this licence.

For peer review only

Addressing alcohol-related harms in the local night-time economy: a qualitative process evaluation from a complex systems perspective

Elizabeth McGill[1*], Dalya Marks[2], Mark Petticrew[2], Matt Egan[2]

1. Department of Health Services, Research and Policy; London School of Hygiene & Tropical Medicine; London, United Kingdom
2. Department of Public Health, Environments and Society; London School of Hygiene & Tropical Medicine; London, United Kingdom

*corresponding author
London School of Hygiene & Tropical Medicine
15-17 Tavistock Place
London WC1H 9SH
elizabeth.mcgill@lshtm.ac.uk

Word count: 9,416 (excluding abstract, tables, figures and references)

CONFIDENTIAL
For peer review only

Abstract

Objectives: English local authorities (LAs) are interested in reducing alcohol-related harms and may use discretionary powers such as the Late Night Levy (LNL) to do so. This study aims to describe how system stakeholders hypothesise the levy may generate changes and to explore how the system, its actors and the intervention adapt and co-evolve over time.

Design: A process evaluation from a complex systems perspective, using qualitative methods.

Setting: A London LA with high densities of residential and commercial properties, which implemented the LNL in 2014.

Participants: Data were generated through interviews with LNL implementers and alcohol consumers, observations in bars and during LNL patrols, and documentary review.

Intervention: The LNL allows LAs to charge late-night alcohol retailers an annual fee (£299 - £4,440) to manage and police the night-time economy (NTE).

Results: When the LNL was being considered, stakeholders from different interest groups advanced diverse opinions about its likely impacts while rarely referencing supporting research evidence. Proponents of the levy argued it could reduce crime and anti-social behaviour by providing additional funds to police and manage the NTE. Critics of the levy hypothesised adverse consequences linked to claims that the intervention would force venues to vary their hours or close, cluster closing times, reduce NTE diversity, and undermine public-private partnerships. In the first two years, levy-funded patrols developed relationships with the licensed trade and the public. The LNL did not undermine public-private partnerships and while some premises varied their hours, these changes did not undermine the intervention's viability, nor significantly cluster venue closing times, nor obviously damage the area's reputation for having a diverse NTE.

Conclusions: This study applies a framework for process evaluation from a complex systems perspective. The evaluation could be extended to measure alcohol-related outcomes and to consider the interplay between the national and local systems.

Keywords: alcohol; complex systems; qualitative research; process evaluation

Strengths and limitations of this study

- This study used a novel design, drawing on a complex systems perspective, to understand the mechanisms by which the intervention may generate system-wide changes.

For peer review only - http://bmjopen.bmj.com/site/about/guidelines.xhtml

- We generated data through a range of qualitative methods, including interviews, observations and documentary review which allowed us to collect data from a wide range of sources.
- We include data from implementers, NTE users, business owners and staff but not health service workers.
- The evaluation occurred after the intervention started, although many of the documents reviewed were produced prior to implementation.

CONFIDENTIAL

For peer review only

INTRODUCTION

In England, alcohol misuse is the largest risk factor for poor health and early mortality for adolescents and adults aged 15-49 years [1], a pattern that is mirrored globally [2]. In addition to the health harms associated with alcohol consumption, alcohol contributes to broader societal harms including crime, violence, anti-social behaviour and disorder [3], many of which occur within the context of the night time economy (NTE) [4]. Different approaches to reducing alcohol harms have been proposed and put into practice. For example, modifying alcohol availability (physical, temporal and economic)[5 6], policing and community safety interventions[7 8], attempting to promote corporate responsibility[9], encouraging certain types of alcohol outlets while discouraging others[10], and encouraging individuals to change consumption behaviours[11]. An evidence synthesis by Martineau et al found that evidence tended to support state enforced regulations restricting the availability of alcohol for sale over non-regulatory approaches[12]. A recent critical review[6] of research on availability broadly reinforced this finding but noted limitations to the evidence base. For example, much of the evidence came from a narrow range of countries (particularly Australia and USA) raising concerns about wider generalisablity and few studies examined effects of changing temporal alcohol availability (hours of sale, including opening and closing hours). A number of studies from the UK and elsewhere have evaluated local-level regulation of alcohol availability and NTEs[13-25]. In England, changes to alcohol licensing systems that included new discretionary powers for local authorities have been the focus of recent and ongoing research[13-16 26].

At the turn of the 21st century in England, there was growing public discourse and concern about the rise of the "alcohol-fuelled, consumption-driven, night-time high street" [27 p.466] which was characterised by clusters of late-night establishments and a tension between those enjoying nights out, those employed within or profiting from the NTE and those impacted by violence, anti-social behaviour and nuisance [28 29]. A series of sweeping legislative and regulatory changes were made, with claims made that this would create a safer NTE, while generating economic benefits to businesses, the people they employed and to governments [28 30]. These changes included the Licensing Act 2003 (enacted in 2005) which transferred responsibility for alcohol licensing from magistrates to local authorities (LAs) and removed fixed closing times for alcohol-retailing venues [30]. The sale of alcohol in England is therefore overseen by LAs, also referred to as Councils, through licensing, trading standards and planning bodies [31]. In this context, LAs have access to a range of discretionary powers to tackle alcohol-related harms, including Cumulative Impact Policies and Early Morning Restriction Orders, both of which were introduced in the Licensing Act 2003.[32] Another discretionary power, which will be the focus of this evaluation, is the Late Night Levy (LNL) which was introduced in the 2011 Police Reform and Social Responsibility Act.

For peer review only - http://bmjopen.bmj.com/site/about/guidelines.xhtml

Late Night Levy (LNL)

The LNL was designed to "empower local areas to charge businesses that supply alcohol late into the night for the extra enforcement costs that the night-time economy generates for police and licensing authorities" [33 p.1]. The intervention aims to prevent and address disturbance and crime associated with late night drinking. The power is discretionary and LAs can choose, following a period of local consultation, to implement a levy on all establishments in the on- and off-trade that have a licence to sell alcohol between midnight and 6 am. The amount each premise pays is set out in a nationally-determined fee schedule based on the rateable value of the premise and the degree to which the premise was primarily alcohol-led (Table 1). Individual LAs may exempt certain types of premises, such as those operating within a Business Improvement District (BID), and/or offer reductions for premises engaging in schemes such as Best Bar None or PubWatch. BIDs, Best Bar None and PubWatch are business- and alcohol industry-led schemes and businesses voluntarily participate in them. These initiatives are supported by public bodies, including LAs or the Home Office. As shorthand, these schemes will be referred to as public-private partnership (PPP) schemes. The revenue from the levy, following the deduction of administrative costs, must be split with a minimum of 70% going to the police and the remainder to the LA. In 2011, The Home Office estimated that the levy would likely be viable in 94 of the 378 LAs across England and Wales and generate a total net revenue of £12.1m per year [34]. The legislation was enacted in 2011 and Newcastle City Council was the first to adopt the levy in November 2013.

Table 1: Late Night Levy charges

**Rateable value**	A: No rateable value - £4,300	B: £4,301 - £33,000	C: £33,001 - £87,000	D: £87,001 - £125,000	E: £125,001 + above	D x 2 multiplier applied to premises in category D that are primarily / exclusively alcohol-led	E x 3 multiplier applied to premises in category E that are primarily / exclusively alcohol-led
**Annual levy charge**	£299	£768	£1,259	£1,365	£1,493	£2,730	£4,440

Source: Home Office 2015 [35]

The LNL as an event in a complex system

Public health researchers have become increasingly interested in applying a complex systems perspective to analysing the multiple interactions that lead to patterns of health behaviour, outcomes and inequalities across communities [36-38]. Where LA's choose to implement the LNL, it is introduced locally into a complex system that interacts with regional, national and international

For peer review only - http://bmjopen.bmj.com/site/about/guidelines.xhtml

systems. A system is a group of elements, bounded in some way, that interact with each other [39 40]. A complex system is one that is characterised by unpredictability and change over time [41 42]. Complex systems exhibit emergent properties that cannot be reduced to the behaviour of the individual system elements [43]. Elements within a system respond to internal and external system inputs; these responses may feedback on the inputs themselves, either amplifying or dampening their impacts, which may, in turn, create unanticipated or unintended effects [44 45]. Analysing a complex system encompasses making sense of the system's trajectory, considering how it is influenced by its previous history and the interactions between its elements [42 46]. Key concepts from a complex systems perspective, which we consider in this paper, are defined in Table 2.

Table 2: Complex systems concepts

Concept	Definition
**Elements**	Components within a system ('agents', institutions, resources, etc.) [42]
**Boundaries**	The 'limits' or 'bounds' of a given system; boundary judgements may be made by system actors (first-order) or researchers (second-order) [40 47]
**Levels**	The structure of the system; levels may operate horizontally and/or vertically depending on boundary decisions [45 48]
**Relationships and interactions**	Connections between different system elements, within and across system levels, and between elements and the broader context [49]
**Local rules**	The norms and principles that guide interactions between system elements and drive system behaviour [50]
**Perspectives**	The different ways actors within the system may view the system, their goals and actions and boundary decisions [51]
**Non-linearity**	Inputs into a system may lead to a non-correspondingly-sized impact [47]
**Feedback**	Responses that either amplify or dampen the impacts stemming from an intervention and may alter the intervention itself [44]
**Adaptation**	The ways in which system elements and the system as a whole respond in response to internal and external inputs [41]
**Emergent properties**	The emergent, collective behaviour of a system that cannot be reduced to its individual parts [52]
**Co-evolution**	The changes to a system and the broader systems in which it is located, over time [42]
**Unintended consequences**	Processes and impacts that were unanticipated at the design stage of an intervention [45]
**System trajectories**	The evolution of a system over time, which is path dependent or constrained in some ways due to its history [42 46]

Complex systems are characterised by their open boundaries and as a result, they interact with, influence, and are influenced by, other systems [53]. From a geographical perspective, they can be characterised by both horizontal and vertical complexity. Horizontal complexity refers to the relationships between system elements, and between systems within the same geographical scale. Vertical complexity refers to the relationships and interactions across geographical scales, with, for example, an emphasis on how international and national systems may influence, constrain and

For peer review only - http://bmjopen.bmj.com/site/about/guidelines.xhtml

CONFIDENTIAL For peer review only

shape local systems [54 55]. A recent scoping review of complex systems perspectives applied to alcohol consumption and prevention found that much of the research in this field is conducted in sub-local (e.g. individual, families, social networks) or local (e.g. neighbourhood, town, cities) systems. Far less consideration is given to the ways that the local systems interact with the national or international systems [55].

A complex systems perspective applied to public health evaluation involves analysing the multiple ways in which a complex system and an intervention interact and influence each other to generate health impacts and health inequalities [56-59]. Evaluators might consider interventions as 'events' within systems that have the ability to disrupt system behaviour, generating evolving and adaptive patterns of behaviour and emergent outcomes [46 60].

In public health, process evaluations have traditionally been used to understand the mechanisms by which interventions leads to impact, the influence of the broader context on observed variations in impact, as well as to assess intervention fidelity and the quality of implementation [61]. Applying a complex systems perspective to a process evaluation can be used to first describe the system, understand its elements, boundaries and the 'rules' or norms that govern the behaviour of its elements and the ways in which they interact each other. Following the introduction of an intervention such as the LNL, a process evaluation with a complex systems perspective then aims to understand the mechanisms by which the elements within the system, and the system as a whole adapt and co-evolve in response.

This process evaluation was conducted in one London LA with the dual aims of 1) describing the system into which the LNL is introduced and synthesising stakeholder hypotheses about the ways in which the levy may generate change within the system; and 2) exploring how the intervention acts as an event within the system, with an emphasis on understanding how the system, its actors and the intervention adapt and co-evolve over time.

METHODS

Study design and data generation

We applied a framework for process evaluation using a complex systems perspective to data we collected on the LNL in one LA [57]. This evaluation framework consists of two phases: Phase 1 involves producing a static system description and developing hypotheses of how the system may change in response to the intervention; Phase 2 analyses the system as it undergoes change following implementation. The evaluation approach is adaptive and hypotheses generated in Phase 1 are intended to inform the evaluative focus of Phase 2. In Phase 2, evaluators should be open to

For peer review only - http://bmjopen.bmj.com/site/about/guidelines.xhtml

exploring unintended processes that stem from the intervention, that may not have been considered at the design stage or in Phase 1 of the evaluation.

The Standards for Reporting Qualitative Research (SPQR) checklist is provided in Supplementary Material 1.

Intervention and setting

The process evaluation was conducted in an English LA located in a metropolitan area with a large NTE. The LA held a consultation on the levy at the end of 2013 and implemented the LNL in late 2014. The levy hours are set at 00:01 to 06:00 and businesses that demonstrate commitment to best practice, as defined by the LA, are eligible for a 30% reduction of the levy fee. Businesses that are a member of the local BID, which requires members to pay a levy separate to the LNL, are neither exempt from the levy nor granted an automatic reduction in the fee. The Metropolitan Police and the LA chose to pool the net amount of levy payments to deliver one broad programme consisting of two different strands: i) additional dedicated police resource to coordinate NTE policing and conduct support and enforcement activity; and ii) a visible street-based patrol service delivered by a police-accredited community safety company four nights per week to give assistance to the licensed trade and members of the public. A Late Night Levy Board with representation from licensees oversees the use of funds raised through the levy.

Sampling and data generation

A complex systems perspective encourages evaluators to consider the intervention as a multi-stage process that, in the instance of the LNL, began with changes in national policy, then a local consultation, and finally local implementation. Local delivery processes could continue to interact with national (or other 'non-local') developments. However, this evaluation focuses primarily on the local system: a focus on horizontal complexity. This local focus represents a 'secondary boundary judgement' [47]; that is one that is made by evaluators (compared to a 'first order boundary judgement' which is made by actors operating within the system).

The sampling strategy aimed to capture a range of different actors and perspectives within the national and local systems in order to contrast how different actors perceive, respond and adapt to the introduction of the intervention. Given the evaluative focus on the LNL in one LA, the sampling strategy was designed to primarily collect data from local actors through interviews, observations and a documentary analysis. However, recognising that complex systems are open systems, the

For peer review only - http://bmjopen.bmj.com/site/about/guidelines.xhtml

sampling strategy was intentionally wider than the local system and the documentary analysis also included national data in order to analyse vertical systemic relationships.

In this process evaluation, Phase 1 focuses on the period prior to local implementation, which included the national policy change and the local consultation. Phase 2 focuses on the local implementation stage and is the stage at which we became involved in evaluation. Data collection for Phase 1 was largely retrospective, but based on primary documentary sources generated during the earlier time period. Phase 2 was based on interviews, observations and document analysis collected during the first two years of the levy's implementation.

A range of data collection methods were utilised, including: a review of national and local documents, interviews with those implementing and delivering the LNL locally (n=12), interviews with users of the NTE (n=9), observations of community safety patrols (28.5 hours) which included informal conversations with patrol officers (n=10) and observations in pubs and bars (6 hours). Table 3 shows the documents analysed and their publication dates; Table 4 provides details of the primary data collection. To preserve participant anonymity, generic job roles are presented to remove identifying information. Data collection and fieldwork was conducted by EM, a research fellow with experience of a range of qualitative methods and analysis.

Table 3: Documents in documentary review

	**Title**	**Organisation (Date)**
**National documents**	Police Reform and Social Responsibility Bill Research Paper 10/81	House of Commons Library (2010)
	Impact Assessment for the alcohol measures in the Police Reform and Social Responsibility Bill	Home Office (2011)
	Police Reform and Social Responsibility Act (2011)	Act of Parliament (2011)
	The Government's Alcohol Strategy	Home Office (2012)
	Next steps following the consultation on delivering the Government's Alcohol Strategy	Home Office (2013)
	Amended guidance on the late night levy	Home Office (2015)
	The late night levy	House of Commons Library (2015)
	Modern Crime Prevention Strategy	Home Office (2016)
	Policing and Crime Bill: Changes to the Late Night Levy – Impact Assessment	Home Office (2016)
**Local documents**	Annual Public Health Reports (n=5)	Council (2011 – 2016/17)
	Licensing Policies (n=2)	Council (2011 – 2017)
	LNL Consultation	Council (2013)
	LNL Consultation Responses (n=338)	Council (2014)
	LNL Written Consultation Responses (n=31)	Council (2014)
	LNL Council Meeting Minutes	Licensing Committee (2014)
	LNL Year 1 and Year 2 Reports	Council (2016; 2017)
	LNL Year 1 and Year 2 Reports	Community Safety Company
	BID Annual Reports	BID Board (2015/2016; 2016/2017)

For peer review only - http://bmjopen.bmj.com/site/about/guidelines.xhtml

Table 4: Primary data collection

	Participants	Number (details)	Year
**Interviews (n=21) (10.4 hours)**	Local authority managers and officers relevant to licensing and public health	4 (one individual interview; three interviewed as a group)	2014
	Police officers	3 (individual interviews)	2016
	Community safety officers	5 (two individual interviews; two interviewed as a pair)	2014, 2016
	Users of the NTE	9 (interviewed in pairs or one group of three; Fridays between 20:00 – 21:30)	2016
**Observations and informal conversations (35.5 hours)**	LNL-funded, community safety patrols; 5 different officers	2 (18.5 hours; 5 officers; Friday 21:00 – 7:00 and Saturday 21:30 – 8:00)	2016
	Non-levy, community safety patrols; 5 different officers	3 (10 hours; 5 officers; Tuesday 6:00-9:00; Wednesday 13:00-20:00)	2016
	Quarterly review meeting (Local authority managers; community safety company managers)	1 (1 hour; 4 participants)	2016
	Pubs and bars (observation only)	4 (6 hours; Fridays between 19:30 – 22:00)	2016

Documents were identified through online searches which included searches of national and local government websites for documents about the LNL, alcohol and health and crime and safety. In addition, Google searches were undertaken using the term 'late night levy'. Documents were included if they shed light on the rationale and process for developing and implementing the levy, or reported on the levy following implementation. All documents are located in the public domain. Some of the documents included what might be considered 'outputs' in a process evaluation and short-term social and health impacts following intervention implementation. The analysis of these data focused on how they were presented, for what purposes, by which actors and how they suggested early indictors of change stemming from the intervention. We report some of the data from these documents in our Results section.

For peer review only - http://bmjopen.bmj.com/site/about/guidelines.xhtml

Interviews with professionals implementing and delivering the LA's (Table 4) LNL followed a topic guide (Supplementary Material 2) and asked participants about alcohol-related challenges, their experience of the LNL and the system in which the intervention is located. Topic guides were semi-structured to allow the participant scope to guide the conversation based on their experiences and understanding of the local system and the intervention. The interviews were audio-recorded and transcribed.

Observations were conducted during five community safety patrols partly funded by the LNL in which addressing alcohol consumption and associated harms was either a primary or secondary focus of the patrol. The observations were semi-structured; an observation template (Supplementary Material 2) was utilised to systematically capture elements of the patrols, as well as be open to capturing observations not envisaged at the research design stage. During the patrols, the fieldworker engaged in informal conversations with patrol staff and observed their actions and engagement with individuals or groups, including staff from licensed premises, police officers, users of the NTE, street drinkers and rough sleepers. In total, ten officers conducted the patrols, two of whom were also formally interviewed prior to the patrols. Throughout each patrol, the fieldworker wrote notes when appropriate and, where possible, captured direct quotations from patrol officers. An additional observation was conducted during a LNL review meeting between managers from the LA and community safety company.

In order to better understand how users of the NTE experience the local alcohol system and the LNL, interviews were conducted in pubs and bars. Nine participants were recruited from alcohol-retailing venues; the fieldworker approached groups of 2-3 drinkers for semi-structured interviews following a topic guide (Supplementary Material 2) that asked about the local area, particularly its NTE and their views on the LNL. Due to the setting, the fieldworker did not take notes during the interview or record the discussion. Notes, including any direct quotations, were written immediately following each interview.

Ethical approval for this study was obtained from the London School of Hygiene & Tropical Medicine Ethics Committee (ref: 10129) with particular attention paid to consent and safety issues around collecting data in situations where alcohol is consumed.

Patient and Public Involvement

No patients or public involved.

For peer review only - http://bmjopen.bmj.com/site/about/guidelines.xhtml

CONFIDENTIAL
For peer review only

Analysis

Phase 1

The framework for process evaluation from a complex systems perspective using qualitative methods suggests several questions to guide Phase 1 of the evaluation: 1) What is the system of interest and what are its boundaries? 2) What are the characteristics of the system and how does it behave at the initial timepoint? 3) What are system stakeholders' perceptions about the ways in which the intervention could lead to changes within the system, including changes that may be unanticipated by intervention designers [57]? The 'Intervention and Setting' section above sets out the local system of interest and its boundaries, which for this evaluation, are the geographical boundaries of the LA. The third question is the focus of this phase of the evaluation.

The analysis began with an in-depth reading of all transcripts, fieldnotes and documents and a deductive approach to coding the data was undertaken, guided by a number of concepts from systems thinking which included: elements, boundaries, levels, relationships and interactions, perspectives and history (see Table 2). The coding process was used to make sense of the national and local histories that created the conditions for the development and implementation of the LNL, the goals of different actors and how their perspectives influenced their views towards the levy.

The emphasis of the analysis in Phase 1 was to utilise the data to synthesise and articulate stakeholder hypotheses about the ways in which they believed the intervention could lead to changes within, and beyond, the system into which it is introduced. In order to do this, a coding framework in the form of a map was developed utilising Visual Understanding Environment (VUE) software [62] (Supplementary Material 3). In order to develop the framework, a list of variables relevant to the LNL, nationally and locally, was independently generated by two researchers (EM and ME) from the coded data. The variables and the relationships between them were then represented visually on a map in order to depict the ways in which stakeholders hypothesised the levy could generate change within the local system. The analysis of Phase 1 was completed before the Phase 2 analysis so that it could inform the analytical focus for Phase 2.

Phase 2

In Phase 2 of the process evaluation framework, the evaluator seeks to understand how the system and the intervention itself change following implementation, exploring the mechanisms by which change occurs [57]. The hypotheses put forth by system stakeholders articulated in Phase 1 were used to guide the analysis. In Phase 2, the focus of the evaluation was on the new actors that were introduced into the system with levy funding. Guided by the four main hypotheses identified in

For peer review only - http://bmjopen.bmj.com/site/about/guidelines.xhtml

Phase 1, there was a simultaneous focus on the system elements and the system as a whole, considering how they adapt and co-evolve over time, disrupting the local system rules and patterns of behaviour. The coding and analysis were led by EM, with analytical discussions taking place across the research team. NVivo 12 was used to aid the data analysis [63].

RESULTS

Phase 1: system description and system stakeholders' hypotheses

System stakeholders articulated four hypotheses about the ways in which the levy could generate change within the local system. In the following section, each of these hypotheses will be described and visually represented.

Hypothesis 1: Increased resources

The first hypothesis, as articulated by those designing, implementing and delivering the intervention, was that the LNL would increase the resources available to police and manage the NTE, which would be used for street-based community safety and policing and additional street cleaning services. These services, would, in turn, lead to a number of positive impacts for residents, visitors and commercial actors (Figure 1):

> *This will produce additional funding for the council and police to use to address the impacts and strains on local services that occur between midnight and 6am in [LA]. […] we believe that the LNL can be used to reduce the instance of crime, disorder and anti-social behaviour during the levy hours as well as improve the local environment.* (LNL Consultation, 2013)

Figure 1 about here

Some residents and visitors further described the mechanism by which such change would occur, placing an emphasis on the additional police and community safety presence; for example, one woman we interviewed in a pub believed more police on the street "means I can walk home safely at 2 am." Others described police as a deterrent for anti-social behaviour and noise:

> *I live on a side street of a late licensed premise and am woken up between 2 am and 7 am regularly every Saturday and Sunday morning. I don't think they realise the noise they're making so if there was a police presence I don't think they'd be as boisterous.* (Consultation response, resident)

For peer review only - http://bmjopen.bmj.com/site/about/guidelines.xhtml

It was theorised that a safer NTE would enhance the overall reputation of the area, driving up visitor numbers and encouraging individuals to spend more money in local establishments. Implicit in this argument was that the levy fee would be easily offset by increased customer numbers, thereby making the levy beneficial for both commercial actors and the LA:

> *the money's going to pay for more policing, and [licensees] get more policing and that is beneficial for them because the safer an area is, the more people that come to the area and the more money that gets spent and the more money they make.* (Interview, Community safety officer)

Hypothesis 2: Reduced support for PPP schemes

A second hypothesis, articulated by businesses, was that licensees would be disinclined to continue to support PPP schemes because the resources do so would be re-directed to paying the levy:

> *If operators do choose to pay the levy then it will impact on funding they can provide for partnership initiatives such as BIDs, Pubwatch and Best Bar None which the Council should look to support and promote in preference to a levy.* (Consultation response, Pub company)

Many licensees in particular expressed concern about the LNL's impact on the BID, which funds dedicated police officers and cleaning services. In consultation responses, licensees agued they would vote against the BID when it came up for renewal, which in turn, would cause the BID to fail: (Figure 2): "As a BID payer if the levy were to come into force I would be voting no the next time the bid comes up for tender" (Consultation response, licensee).

Figure 2 about here

If the BID were to fail as a result of the LNL, licensees theorised that there would be a range of unintended consequences in the local system. These included reducing the overall resources available to manage and police the local area and damaging economic impacts because the BID is intended to work to create an environment that encourages residents and visitors to the area:

> *It is a possibility that nearly 40 licensed premises in the [local area] BID area will not vote for the BID again if this means that they pay two levies instead of only one. A BID needs a majority by numbers and also rateable value to succeed. A failure to achieve either one of these would therefore, jeopardise the provision the BID makes for policing and cleaning as well as what we do to ensure a good shopping environment for local people, Christmas lights, hanging baskets, support for community events and much more.* (Consultation response, Pub manager)

The local BID Board also perceived that any negative impact of the levy on the BID could generate impacts beyond the boundary of the local system. These system stakeholders argued that this hypothetical impact should be considered within the Mayor of London's goal to

For peer review only - http://bmjopen.bmj.com/site/about/guidelines.xhtml

increase the number of BIDs throughout London:

> *BIDs are burgeoning in London and the Mayor has set a target for a number of additional BIDs by 2015. It would be a loss, not just to [LA] but to London as a whole should [BID name] not get re-elected and become the first BID in London to fail.* (Consultation response, BID Board)

Hypotheses3 and 4: Premises will (3) vary hours or (4) close due to unwillingness or inability to pay the Levy

In response to the Council's consultation, 42% of businesses reported they would voluntarily change their permitted licensing hours in response to the introduction of the levy. A smaller number argued that the levy would force some businesses to close as they became economically unviable. Consultation submissions from business hypothesised that these possible responses could lead to a range of unintended consequences, including undermining the levy, re-introducing a 'terminal hour,' reducing the diversity of late-night provision and ultimately generating negative economic consequences to the local area in the form of reduced employment and local investment (Figure 3). Nearly all of these claims about potential impacts were made without reference to research evidence (and in fact, as will be raised in the Discussion, there is evidence that challenges these impact claims). However, one pub company's consultation submission did cite a study before going on to develop unsupported claims about the lack of ned for, and impact of, the levy. The study referenced [64 65] had found changes from fixed to staggered closing times were not associated with changes in overall violence but "may have contributed to additional problems by spreading violence into the early hours of the morning" [65 p.8]. The pub company's submission referred to these study findings and then claimed they should be interpreted as meaning that more policing was not needed for the NTE and that the levy would not lead to extra policing:

> *Overall the net effect should be that same level of policing is required as previously. Cynics may suggest the Late Night Levy is a tax-raising measure to pay for the same level of policing that has always been provided.* (Consultation response, Pub company)

Only two consultation responses, submitted by Public Health and the Clinical Commissioning Group, considered the LNL in terms of its ability to reduce alcohol consumption and associated harms by restricting the availability of alcohol. In contrast, all other system actors discussed the levy in terms of addressing the harms associated with acute intoxication, focusing primarily on disturbance, anti-social behaviour, crime, and to a far lesser degree on health-related indicators such as ambulance call-outs or hospital admissions. In this sense, discourses around reducing or preventing alcohol consumption (primary prevention) were largely absent, with a focus instead on making the NTE a safer space for consumption and the possible economic and cultural impacts of the levy.

For peer review only - http://bmjopen.bmj.com/site/about/guidelines.xhtml

Figure 3 about here

As businesses shut early, or closed entirely in response to the levy, business actors hypothesised that the LA would fail to generate sufficient revenue to provide the new proposed services: "We remain to be convinced that the LNL will raise the amounts of money anticipated, as a significant number of permissions within [LA] are likely to be withdrawn, by way of the free minor variation procedure." (Consultation letter, Pub company and brewer). This represented an example of a perceived negative feedback loop; it was hypothesised that as fewer businesses remained to contribute to the levy through late-night provision, the ability for the levy to continue as an intervention would be jeopardised.

If businesses varied their operating hours to avoid the levy, some in the licensed trade argued, without reference to research evidence, that this could effectively re-introduce a 'terminal hour' whereby many premises close at the same time, which would lead to an increase in crime and anti-social behaviour:

> *If a number of premises reduce their hours as a result of the levy, this could potentially create anti-social behaviour issues with a large number of premises closing at the same time and a return to the spike of crime, disorder and nuisance and midnight observed across the country prior to the introduction of the Licensing Act 2003.* (Consultation response, Trade organisation representing on-licence premises)

Some system actors expressed concern that smaller, independent businesses, as well as those which are not alcohol-led, would be most affected by the levy, leaving a less diverse NTE dominated by pub chains and clubs. A reduction in diversity was theorised to make the LA less attractive, which, in turn, was hypothesised to have the potential of leading to negative economic impacts as customers choose to go elsewhere, moving beyond the boundaries of the local authority:

> *Many operators will have to curtail their hours irrespective of the economic consequences, thereby reducing the number of post-midnight premises in the borough. […] visitors to the Borough's late night economy [would be] choosing other areas of London where no such restrictions apply with obvious economic consequences for [LA]'s late night economy and the businesses that rely on it.* (Consultation response, Operator of managed pubs)

In underscoring how elements of the system are interconnected, a number of businesses suggested that the LNL would have negative economic impacts that affect more than just late-night alcohol retailers, making the LA a less appealing area to operate a business:

> *I am currently looking at sites in the borough; I run a high end food and drink offer, if this levy is introduced I would have to look if the operation could still be viable. My venues do not run beyond midnight but I understand that early evening venues are*

For peer review only - http://bmjopen.bmj.com/site/about/guidelines.xhtml

intrinsically linked to the later venues and if these were to close or relocate it would reduce footfall in the areas affected. (Consultation response, prospective licensee)

Ultimately many businesses argued that the LNL, through changes to opening hours, lower profit margins, premise closures and lack of investment in the local area, would result in negative economic consequences and job losses for the LA as a whole:

The council will further kill off the high street if they implement this levy. Pubs and bars will re-locate to other nearby locations where the levy is not in place and lose a number of job opportunities for local people. I thought the council's major objective as to increase employment opportunities for local people, not decrease it. (consultation response)

Phase 2: early implementation and mechanisms of system change

The levy began on November 1, 2014 and in the first year, fees were collected from 338 licence holders.

Hypothesis1: Increased resources

The key hypothesis as described by those who designed and implemented the levy, was that it would bring in additional resources to manage and police the NTE (Figure 1). In the first year the levy raised £397,278 and in the second year £377,122 (Council LNL Year 1 and 2 Reports). While these figures were lower than the Council's projected £450,000, the Council described these as sufficient to plug an "an identified gap" in managing and policing the NTE (Council, LNL Year 1 and 2 Reports). The additional resources were utilised to fund an NTE-specific police team and a four-person community safety patrol, delivered by a police-accredited, private company, that worked Thursday – Sunday nights from approximately 8 pm – 8 am.. The new community safety service is the primary focus of Phase 2 of this process evaluation; an overview of the structure of the new service is provided in Table 5, along with output data from the service provider's annual reports.

Table 5: Community safety service

**Patrol description:** The patrol met at 8 pm and conducted a 'scan' of the borough, driving down main roads and stopping to address any issues they identified, such as visible pre-loading. At 10 pm the officers attended a briefing at the police station which included: 1) a police briefing for all officers on duty and 2) a NTE briefing for the NTE police patrol and the community safety officers. Following the briefing, the community safety officers patrolled the borough throughout the night, conducting a number of 'taskings' (which came from the Police, the Licensing Team or were self-generated), responding to calls from venues, identifying and responding to individuals and groups and patrolling areas where there were hyper-local 'kick-out times'. The patrol concluded around 8 am.		
**Strands of the service**	**Year 1**	**Year 2**
Welfare	316 checks	724 checks
Medical	161 individuals	97 individuals
Addressing anti-social behaviour, aggression, urination, pre-loading	365 incidents of violent or aggressive behaviour,	784 incidents of violent or aggressive behaviour, 675

For peer review only - http://bmjopen.bmj.com/site/about/guidelines.xhtml

	451 dispersals, 738 warnings about conduct	dispersals, 1,235 warnings about conduct
Support to the licensed trade	2,295 liaisons with licensed trade; 226 responses to calls	2,482 liaisons with licensed trade; 125 responses to calls
Intelligence gathering	620,459 words	620,292 words

Source: Community Safety Company, LNL Year 1 and Year 2 Reports

A key component of the new patrol service, which significantly increased from Year 1 to Year 2, was engagement with users of the NTE to ensure their welfare and to intervene early in anti-social behaviour, disturbance and nuisance to prevent its escalation:

> *So not only are they there to deal with the response side of it, but it's to try and prevent that happening in the first place, so to deal with those people who potentially would go on and commit further offences because they've started shouting and swearing and causing problems with someone up this end of the street. By the time you get down the other end, they've stopped in five other pubs on the other way, not been challenged, not been highlighted to anybody on the way down, although their behaviour's getting more and more rowdy. Then they go in, have a fight or cause a disturbance and need for police action further down the road.* (Interview, Police officer)

The welfare aspect of the service, which included community-safety officers helping members of the public, was also considered a critical component of the service and as shown in Table 5, increased significantly from Year 1 to 2. The officers also provided medical care and the medical service represented an evolution of the service. While it was always within their remit to have a first-aid trained officer, they expanded this provision shortly after starting the service and purchased additional medical equipment. In addition to supporting members of the public, the medical side of the service was seen as a low-cost mechanism to reduce the burden on the London Ambulance Service and NHS. In the first two years the service reported preventing or cancelling the dispatch of 54 and 57 ambulances, respectively, which they calculated as savings of £16,200 and £14,478.

The Council reported a 17% reduction in alcohol-related crime between midnight and 8 am and a 14.4% reduction in alcohol-associated violence compared to the previous 12 months, although they assumed that this was not all attributable to the levy. They also reported a large increase (29-30%) in calls to the police and anti-social behaviour line about alcohol-related incidents which they further argued justified the need for the levy funding (Council, LNL Year 1 Report). In Year 2 the Council reported a 21% reduction in alcohol-related crimes compared to the previous 12 months, and a 24% decrease in anti-social behaviour calls (Council, LNL Year 2 Report).

For peer review only - http://bmjopen.bmj.com/site/about/guidelines.xhtml

Engagement with the licensed trade

Whilst Hypothesis 1 emphasised the resources to police and manage NTE users in the area, the new patrol service also sought to develop relationships with local actors. Notably, they tried to develop relationships directly with the licensed trade – to monitor and support licensed operators to encourage safer business practices aimed at minimising anti-social behaviour within and outside the premises. In the first year of the levy funding, the patrol provided an introductory visit to 251 of the venues on the levy, which they argued was as an important mechanism to overcome hostility towards the levy and it's funded patrols.

Outside of the initial visits, the patrol worked to develop relationships and trust with venue staff through the repeated interactions; a key element of this, which they contrasted with the police, was the deployment of the same officers every night, particularly in the first year of the service:

> *One of the things you absolutely have when you're any form of policing, really, you've got to have that consistency. You've got to have the relationships. That comes from, you know, repetition. It's from meeting the DPSs [designated premise supervisors] on a regular basis, building up a trust and an understanding of what you're there to do [...]. Well if you're on rotation you can't possibly know. You wouldn't even know who that person is and you certainly wouldn't be able to kind of build a balanced intelligence picture.* (Interview, Community safety officer)

The Council in its report on the levy following the first year of implementation similarly underscored that the patrol was "resourced by regular officers" and highlighted the relationships they developed with businesses:

> *Not only have the Nightsafe Patrol Officers have developed a good working relationship with licence holders and their door staff the team have acquired excellent working knowledge or the night-time economy in Islington and made a significant contribution to information gathered by the police and Local Authority.* (Council, LNL Year 1 Report)

When probed, community safety and police officers described an evolution of the relationships such that many licensees began to engage with the service, overcoming their initial resistance:

> *We came up initially against a lot of unhappiness because it's another tax effectively, a levy on these premises. They don't want to pay it. They're already paying ridiculously high rates and other business taxes and stuff. So, but, you know, I get that. But we're seeing a change now, you know. A year, 18 months down the road, they can see a benefit to it, so [...], if they need help they'll get help. You know, they'll prevent stuff happening and hopefully make their business more attractive.* (Interview, Police officer)

Others licensees, however, remained what the officers referred to as 'hostile venues,' continuing to oppose the levy and its associated services. Officers put this down to a misunderstanding of the service's remit: "they [the licensees] see it as an enforcement role instead of a support role." (Excerpt from fieldnotes)

For peer review only - http://bmjopen.bmj.com/site/about/guidelines.xhtml

The community safety service was tasked with collecting intelligence to help the police or inform licensing decisions. Key to this intelligence-gather strategy was developing cooperative, rather than adversarial relationships with venue managers and staff, as described above. Information gathering and sharing amongst police, patrol and licensed venue operators was reciprocal, or in system terms, represented a positive feedback loop. Closer relationships amongst these three groups of actors appeared to emerge as a consequence, along with an 'othering' of certain venues who remained outside of this information sharing sub-system. Furthermore, the information gained was used to inform licensing decisions::

> *And [the community safety officers] assist us as well. Not just us as licensing officers, but the police on the whole, because within our briefings we can say to them, just little things that have happened, that you wouldn't normally get a chance to deal with, can you go and check on this and this, this, this, and just have a look and even in terms of where new applications are coming in and people are asking to do various different things in their licence, and we're thinking, not sure you could do that, but we need to check the place out. […]. And they report back to us, and then that assists us in saying whether someone can or can't have a licence. It's invaluable, really."* (Interview, Police licensing officer)

Through the mechanisms described above, the Council, the police and the community safety officers reported that more venues were operating in a 'responsible manner' following the implementation of the levy. Hence, while the initial hypothesis around extra resourcing focused on policing and management of NTE-users, by the second year of LNL's implementation a new mechanism for impact had emerged through information sharing and relationship building between NTE operators and the agents that patrolled and policed the NTE:

> Interviewer: do you think it (LNL and other licensing policies) has changed kind of how people consume alcohol in the borough?
> Respondent (Police licensing officer): *I don't think it's changed how people consume their alcohol in the borough. I think it's changed how operators operate.*

Taken holistically, the new service was perceived to have changed how actors within the system behaved and interacted with one another, disrupting previous patterns of behaviour as system elements responded and adapted to the new services.

Hypothesis 2: Reduced support for PPP schemes

The second key hypothesis, articulated by businesses, was that if businesses were liable to pay the levy, they would no longer support PPP schemes, particularly the local BID (Figure 2). This initial hypothesis did not accurately theorise how the system would adapt in the first two years of the intervention. Instead, in October 2016 members of the BID "again voted resoundingly for us to

For peer review only - http://bmjopen.bmj.com/site/about/guidelines.xhtml

CONFIDENTIAL
For peer review only

continue" (BID website) and the BID expanded to cover a larger geographical area. Following the introduction of the levy, the BID reported a key priority for safety in their area was: "achieving 24-hour security at [BID area] through co-ordinated working with street patrol [LNL-funded service]" (BID Annual Report, 2015/2016) and a licensee described a reliance on both BID- and LNL-funded patrols:

> *The night time economy is a major contributor to the wealth of the [BID area]. Making sure the environment is fun yet safe is a huge undertaking, not only for us licensees but also for the police and [LA] Council. [BID name] makes sure we are all working together. Not only do we have the [BID-funded] Police Team at our disposal but can also rely on [LNL-funded service]. (BID Annual Report, 2016/17, Bar Owner)*

Prior to the levy's implementation, members of the licensed trade argued that the BID-funded services addressed their policing and safety needs. However, as the LNL-funded community safety patrol became embedded in the local system, some members of the BID came to see the community safety patrols as a complement to their own funded services and promoted collaboration between the two services, leading to greater resources for managing the local environment.

Hypotheses 3 and 4: Premises will (3) vary hours or (4) close due to unwillingness or inability to pay the Levy

The final hypotheses were that a large number of premises would vary their hours in response to the introduction of the LNL, or in some cases close completely, which would lead to unanticipated consequences (Figure 3). The data reported by the Council showed that approximately one quarter of all premises who were initially liable to pay the levy either varied their licence hours or closed prior to the implementation date, which was lower than the 42% who indicated they would during the consultation period. The majority of these businesses varied their hours, rather than permanently closing their doors.

The majority of premises that were identified as being liable to pay the levy continued to operate after midnight and the LA did not see a re-introduction of a 'defacto terminal hour'. However, there remained clusters of bars and pubs that closed at similar time, which the community safety officers would refer to informally as 'kick-out times'.

Members of the licensed trade and some residents and visitors theorised the levy would create an NTE that lacked diversity, which in turn would drive down visitor numbers. During the course of fieldwork, we observed a busy NTE with bustling streets and busy venues. All the users of the NTE we spoke with during the course of fieldwork in the second year of the levy described numerous and diverse places to go out in the LA:

For peer review only - http://bmjopen.bmj.com/site/about/guidelines.xhtml

CONFIDENTIAL
For peer review only

[Name] was talking about how there used to be only one place really to go (The Name – which she says is a great pub), but now there are so many options. The places to go out don't just include alcohol: "It used to be that there were just three places to eat ... [she lists their names] and now there are so many to choose from. (Excerpt from fieldnotes)

Cumulatively, these data show that some premises did vary their hours in response to the introduction of the levy, but the levy remained viable and that an insufficient number of premises closed at midnight to re-introduce a 'terminal hour'. The LA maintained a reputation for providing a diverse and busy NTE following the implementation of the intervention.

DISCUSSION AND CONCLUSION

This two-phased process evaluation sought to describe the local system into which the LNL is introduced and explore how the intervention may lead to changes as the system, its actors and the intervention adapt and co-evolve over time. We identified four main hypotheses put forward by system actors articulating the ways in which they envisaged the intervention would lead to system change, including those that were unanticipated at the intervention design stage.

In Phase 1 of the evaluation, we analysed stakeholder opinions to develop the system map from which we would select hypotheses for analysis. During Phase 1, we observed that stakeholders who were supportive of the LNL (and some stakeholders who were more critical) were preoccupied with 'secondary prevention' which we define as policing and other services that aim to prevent intoxicated NTE customers engaging in violent, anti-social or risky behaviour. We contrast this with primary prevention which aims to deter alcohol consumption in order to prevent intoxication that leads to social problems and to prevent harms to health caused by consumption.[66] A discourse that prioritises secondary prevention arguably aligns with commercial interests in that action to prevent harm is taken after the point of sale.

A second observation we make about our Phase 1 analysis was that we found limited reference to research evidence in stakeholder consultation submissions. Submissions that cited research evidence directly were clear outliers. For those who promote evidence-informed decision-making, this is concerning. We do not assume that stakeholders were simply unwilling to explicitly cite their sources. Previous research on cultures of evidence amongst local practitioners has found a number of barriers to evidence use relevant to this study[67]. These include absence of evidence or inconclusive evidence, difficulty accessing evidence, prioritising local (often experiential) knowledge over published research of different interventions implemented in other contexts and a lack of interest in research that contradicts stakeholder opinions. A study of stakeholder submissions to an alcohol-related World Health Organisation consultation highlighted how evidence can be selected,

For peer review only - http://bmjopen.bmj.com/site/about/guidelines.xhtml

misrepresented or ignored in ways that aligned with stakeholder interests[68]. In our study, the stakeholders who contributed to the LNL consultation could not be informed by a LNL evaluation, as none existed.

Nonetheless, there was still some research evidence available that could have informed stakeholder hypotheses about changes stemming from the levy. For example, some stakeholders claimed that the LNL would increase harms by clustering closing times of alcohol venues. However, this claim is weakened by evidence from an earlier Manchester (UK) study that found changes from fixed to staggered closing times were unrelated to changes in violence[64 65]. One stakeholder submission referred to this study but used its findings to make unsupported claims about the lack of need for more NTE policing and the lack of impact of the levy on policing. Since the Manchester study, international evidence that NTE regulation can reduce harm has increased, most recently with an Australian study[20-22]. That study found that NTE regulation (that includes reduced trading hours) reduced alcohol harms without reducing the number of patrons, scale or diversity of NTEs and occurred while the number of alcohol licensed venues increase[17 18 69].

This disconnect between stakeholder claims and research evidence raises an important point about the value of examining hypotheses derived from stakeholder opinions, when those opinions may lack supporting evidence or even be contradicted by evidence. We suggest a number of reasons why stakeholder-derived hypotheses should be examined. Firstly, stakeholders either believe their claims or at least believe the claims serve some purpose (e.g. self-interest), suggesting a continued need for scrutiny and, where appropriate, refutation. Secondly, it is possible that local stakeholders understand something about a particular context or intervention that is different from the contexts of previous research. Thirdly, stakeholder opinions (even those contradicted by existing evidence) may have a powerful impact on (i) decisions to implement interventions, (ii) willingness to comply with interventions, and (iii) lobbying efforts to discontinue interventions – all of which can be hypothesised to affect intervention impacts (e.g. by preventing an intervention, making an intervention harder to implement or shortening an intervention's lifespan). We note that across England, only 10 LAs currently have a LNL in operation. In other areas, a LNL has been considered by an LA but abandoned at consultation stage or discontinued after implementation [70 71]. Our own findings also challenge stakeholder claims that the LNL would lead to unwanted consequences through clustering of closing times and reductions to NTE diversity and footfall. These findings contribute to a growing evidence base that, we hope, will encourage decision makers to consider such claims with scepticism.

For peer review only - http://bmjopen.bmj.com/site/about/guidelines.xhtml

We turn now to each of the four hypotheses we examined in Phase 2 of our study. The first was that the intervention would increase resources to police and manage the NTE. The intervention did help finance the introduction of new actors into the local system, who through consistent, visible and prolonged relationship building with the licensed trade and the public, sought to disrupt local system rules and develop new practices. Findings from the first two years of the levy suggest that these efforts led to an evolution in the way that many, although not all, licensees viewed the levy and a change in how some venues were managed.

Contrary to the expressed views of some stakeholders, the introduction of the LNL did not undermine PPP schemes during the study period, particularly the BID, as expressed in the second hypothesis. The LNL's implementation co-occurred with an increase in voluntary industry initiatives and partnerships. Previous research on PPPs and industry-led so-called 'social responsibility' activities have highlighted conflicts of interests and called into question their effectiveness in reducing or preventing harm[25 72-79]. The mechanisms by which increased regulation of the sale of harmful commodities might lead to increased voluntary and partnership activity warrants further investigation across different types of interventions and harmful commodities.

With regards to the third and fourth hypotheses, there was some evidence that premises varied their hours in response to the levy, but these changes did not ultimately undermine the viability of the levy, lead to the re-introduction of a terminal hour, nor obviously damage the NTE's reputation as being diverse and vibrant. Taken together, those in charge of developing and implementing the levy at the local level, viewed these early indications of system change as successful. This suggests a reinforcing feedback loop, whereby the perceived success of the levy in this LA ensured its continuation.

This evaluation represents the first application of our complex systems framework for process evaluations [57]. While many in public health have argued that complex system approaches can produce better evidence for decision making that account for real-world complexities [38], there have been relatively few prominent examples of this perspective applied to public health process evaluation to date [80]. This work attempts to address some of these limitations. The use of the framework and explicit application of systems and complexity concepts was utilised to make sense of the broader system into which the levy is introduced, the many processes through which the levy may lead to impacts, many of which might be unanticipated, and the dynamic responses to the intervention that lead to an evolution of the system's actors, their relationships with each other, the intervention, and the system as a whole.

For peer review only - http://bmjopen.bmj.com/site/about/guidelines.xhtml

This process evaluation is also the first known evaluation of the LNL [81 82]. An Institute of Alcohol Studies (IAS) report reviewed the impact of the Licensing Act (2003) ten-years post-implementation and reported that the LNL had the potential to reduce alcohol availability by encouraging premises to shorten their opening hours, could help foster a cleaner environment through the provision of additional street cleaning resources and could be used to promote diversity in the NTE. The report also highlighted other possible impacts of the levy, including that the levy might prevent or damage partnership working between LAs and the alcohol industry, impact the industry's profitability, and be too inflexible a tool to be well suited to many LA's NTEs [81]. The findings from our process evaluation shed light on the mechanism by which these impacts may or may not occur within a local system. Despite acknowledging that there has been no evaluation of the LNL's impact on crime and disorder, a subsequent joint IAS and Foundation for Alcohol Research and Education report argues: "Attempts have been made to limit closing times in areas with acute problems, through the late night levy and early morning restriction order, although these policies have also proven largely ineffective" [82 p.10]. The IAS's judgement on the LNL is premature in the absence of an impact evaluation that examines a range of health, social and economic outcomes. The Queensland Alcohol-related violence and Night-Time Economy (QUANTEM) project provides an example of how such a study could be conducted, using multiple data sources to measure impacts on alcohol-related health and social harms and local economies[17-24]. Furthermore, the ExILEnS study and research stemming from the NIHR School for Public Health Research alcohol programme demonstrate how impacts of local alcohol policies have been evaluated in UK contexts[13 15 16 26]. A well-theorised, robust impact evaluation of the LNL is overdue.

Strengths and limitations

As evaluators, we made two crucial boundary decisions in this process evaluation: to focus on the local level and to include and exclude certain local system variables from our analysis. Together, these represent an emphasis on horizontal complexity. The first decision was made a-priori and was influenced by the nature of the intervention (i.e. a locally-delivered intervention) and our interest in the delivery processes within one LA system. However, there are also vertical complexities that affect, influence and interact with the local system; the local system is embedded within broader regional, national and international systems and the boundaries between them are open [40]. We included some consideration of the national system in order to make sense of the context in which the LNL was introduced as a discretionary power available to LAs, but other stakeholders within the national and local systems, or other evaluation teams might have chosen to broaden their boundaries. Further, the focus on the local excludes learning and evidence from international literature. Given limited evaluation resources and the adoption of a complex systems perspective

For peer review only - http://bmjopen.bmj.com/site/about/guidelines.xhtml

there was a trade-off of breadth versus depth. We chose to prioritise collecting data from a range of system stakeholders over a larger sample of any single stakeholder group. This choice was motivated by the aim of describing and analysing multiple perspectives and views held within the local system. A better resourced study could have included more depth as well as breadth.

We also made decisions about the variables of interest within the local system [83]. This was informed by the data generated through the evaluation and our aim was to focus on the variables we found to be most relevant to the LNL. Examples of this exist within the systems literature, for example, with researchers utilising data generated through documentary review and interviews to develop causal loop diagrams [84]. However, this raises important considerations around power dynamics and who ultimately decides where boundaries are drawn [85]. This work could fruitfully be extended by engaging in processes that invite system stakeholders to participate in the boundary decisions and critique [45 86] and to provide feedback on our synthesis of their perspectives. Specifically, our coding framework, which was depicted on a system map, would have benefitted from refinement by system stakeholders.

A limitation of this evaluation is that we did not collect primary data from residents or those working within the healthcare system. In addition, no data were generated or analysed about the broader economic impacts on the local economy. Conducting systems research often involves collecting data from a wide range of different actors across a given system [41], which is resource-intensive and challenging when conducting smaller, local evaluations. Conducting a documentary analysis is one possible way to include data from a wider range of participants than might be possible through interviews and observations alone. In this evaluation, for example, we collected limited primary data from members of the licensed trade and relied on their extensive consultation responses which provided insight into the ways in which they theorised the levy might lead to a range of unanticipated impacts across the local system.

We collected data for Phases 1 and 2 concurrently in the post-implementation period (although many documents included in our documentary review were produced prior to the levy's implementation). As a result, Phase 1 informed the Phase 2 analysis, but not the Phase 2 data collection. While this approach underscores the flexibility of the process evaluation from a complex systems perspective framework, it also limited our ability to follow all emergent findings. For example, the consultation responses underscored how the levy might affect employment patterns in the local area, with premises having to vary their employees' shift patterns or make some employees redundant. We did not collect data from premises or from the LA that could then speak to these possible impacts.

For peer review only - http://bmjopen.bmj.com/site/about/guidelines.xhtml

Future direction and conclusions

The process evaluation from a complex systems perspective is intended to be adaptive, drawing on early findings to inform subsequent data collection and analysis. A logical next step for this evaluative process is to measure alcohol-related outcomes and to understand the processes beyond the immediate local system of interest, to consider the vertical dimensions of complexity. The evaluation shed light on the possible spillover effects to neighbouring local authorities, and these processes and outcomes could be explored. In addition, in the Modern Crime Prevention Strategy (2016) the Government proposed changing the structure of the levy to apply to specific types of premises or specific hotspots within the NTE, rather than entire LAs. At the end of the same year, the Home Office concluded that the levy had been implemented in fewer LAs that anticipated (n=7) because of criticisms "that LAs consider the levy to be inflexible and the licensed trade has highlighted issues of unfairness in terms of which businesses pay the levy" [87 p.1]. While the changes were to come into effect in 2020, at the time of writing, they have yet to do so. Finally, the Covid-19 pandemic represents a large system shock that has had significant financial impacts on LAs and the licensed trade [88]. An impact evaluation of the LNL is needed, accompanied by a further process evaluation extended to explore these interacting local, regional, national and international processes and systems.

Figure 1 legend:
Orange bubble = national variable; Yellow bubbles = local variables; Green bubbles = immediate theorised impacts stemming from levy introduction Solid line: positive relationship between variables; Dashed line: inverse relationship between variables; Dotted green lines: theorised impacts stemming from the levy introduction

Figure 2 legend:
Orange bubble = national variable; Yellow bubbles = local variables; Green bubbles = immediate theorised impacts stemming from levy introduction Solid line: positive relationship between variables; Dashed line: inverse relationship between variables; Dotted green lines: theorised impacts stemming from the levy introduction

Figure 3 legend:
Orange bubble = national variable; Yellow bubbles = local variables; Green bubbles = immediate theorised impacts stemming from levy introduction Solid line: positive relationship between variables; Dashed line: inverse relationship between variables; Dotted green lines: theorised impacts stemming from the levy introduction

Supplementary material 1: Standards for reporting qualitative research (SRQR) checklist

Supplementary material 2: Data collection instruments

Supplementary material 3: Coding framework

For peer review only - http://bmjopen.bmj.com/site/about/guidelines.xhtml

Contributorship statement: EM designed the methodology, conducted data collection, led on the analysis and drafted this manuscript. ME contributed to the analysis. EM, DM, MP and ME provided input into data interpretation, critically revised the manuscript and approved the final version.

Competing interests: DM is a joint Public Health Strategist/Associate Professor covering the intervention area, but was not involved in the design or delivery of the intervention. The authors declare no other competing interests.

Funding: This study was funded by the National Institute for Health Research (NIHR) School for Public Health Research (SPHR), Grant Reference Number PD-SPH-2015. The views expressed are those of the author(s), and not necessarily those of the NIHR or the Department of Health and Social Care.

Data sharing statement: No additional data are available. The ethical approval granted for this study stipulated that interview transcripts would not be shared beyond the research team.

Ethics statement: Ethical approval for this study was obtained from the London School of Hygiene & Tropical Medicine Ethics Committee (ref: 10129).

Acknowledgments: We gratefully acknowledge discussions with Dr David Humphreys and Professor Niamh Fitzgerald that helped us during data interpretation.

CONFIDENTIAL
For peer review only

REFERENCES

1. Burton R, Henn C, Lavoie D, et al. A rapid evidence review of the effectiveness and cost-effectiveness of alcohol control policies: an English perspective. *Lancet* 2017;389(10078):1558-80. doi: https://doi.org/10.1016/S0140-6736(16)32420-5
2. Griswold MG, Fullman N, Hawley C, et al. Alcohol use and burden for 195 countries and territories, 1990–2016: a systematic analysis for the Global Burden of Disease Study 2016. *Lancet* 2018;392(10152):1015-35. doi: https://doi.org/10.1016/S0140-6736(18)31310-2
3. Rehm J, Baliunas D, Borges GL, et al. The relation between different dimensions of alcohol consumption and burden of disease: an overview. *Addiction* 2010;105(5):817-43.
4. Philpot R, Liebst LS, Møller KK, et al. Capturing violence in the night-time economy: a review of established and emerging methodologies. *Aggress Violent Behav* 2019;46:56-65. doi: https://doi.org/10.1016/j.avb.2019.02.004
5. Wagenaar AC, Salois MJ, Komro KA. Effects of beverage alcohol price and tax levels on drinking: a meta-analysis of 1003 estimates from 112 studies. *Addiction (Abingdon, England)* 2009;104(2):179-90. doi: 10.1111/j.1360-0443.2008.02438.x [published Online First: 2009/01/20]
6. Holmes J, Guo Y, Maheswaran R, et al. The impact of spatial and temporal availability of alcohol on its consumption and related harms: A critical review in the context of UK licensing policies. *Drug and Alcohol Review* 2014;33(5):515-25. doi: https://doi.org/10.1111/dar.12191
7. Siegfried N, Parry C. Do alcohol control policies work? An umbrella review and quality assessment of systematic reviews of alcohol control interventions (2006 - 2017). *PLoS One* 2019;14(4):e0214865-e65. doi: 10.1371/journal.pone.0214865
8. Florence C, Shepherd J, Brennan I, et al. Effectiveness of anonymised information sharing and use in health service, police, and local government partnership for preventing violence related injury: experimental study and time series analysis. *BMJ* 2011;342:d3313. doi: 10.1136/bmj.d3313
9. Babor TF, Robaina K. Public health, academic medicine, and the alcohol industry's corporate social responsibility activities. *American journal of public health* 2013;103(2):206-14. doi: 10.2105/ajph.2012.300847 [published Online First: 2012/12/15]
10. Egan M, Brennan A, Buykx P, et al. Local policies to tackle a national problem: Comparative qualitative case studies of an English local authority alcohol availability intervention. *Health & Place* 2016;41:11-18. doi: https://doi.org/10.1016/j.healthplace.2016.06.007
11. Michie S, Whittington C, Hamoudi Z, et al. Identification of behaviour change techniques to reduce excessive alcohol consumption. *Addiction (Abingdon, England)* 2012;107(8):1431-40. doi: 10.1111/j.1360-0443.2012.03845.x [published Online First: 2012/02/22]
12. Martineau F, Tyner E, Lorenc T, et al. Population-level interventions to reduce alcohol-related harm: an overview of systematic reviews. *Preventive medicine* 2013;57(4):278-96. doi: 10.1016/j.ypmed.2013.06.019 [published Online First: 2013/07/03]
13. de Vocht F, Tilling K, Pliakas T, et al. The intervention effect of local alcohol licensing policies on hospital admission and crime: a natural experiment using a novel Bayesian synthetictime-series method. *Journal of epidemiology and community health* 2017;71(9) doi: 10.1136/jech-2017-208931
14. Gavens L, Holmes J, Buykx P, et al. Processes of local alcohol policy-making in England: Does the theory of policy transfer provide useful insights into public health decision-making? *Health & place* 2017;57 doi: 10.1016/j.healthplace.2017.05.016
15. de Vocht F, Heron J, Campbell R, et al. Testing the impact of local alcohol licencing policies on reported crime rates in England. *Journal of epidemiology and community health* 2016;71(2) doi: 10.1136/jech-2016-207753
16. Fitzgerald N, Egan M, de Vocht F, et al. Exploring the impact of public health teams on alcohol premises licensing in England and Scotland (ExILEnS): procotol for a mixed methods natural

For peer review only - http://bmjopen.bmj.com/site/about/guidelines.xhtml

experiment evaluation. *BMC Medical Research Methodology* 2018;18(1):123. doi: 10.1186/s12874-018-0573-z
17. Miller PG, Coomber K, de Andrade D, et al. Summarising the impacts of the Queensland Alcohol-related violence and Night-Time Economy (QUANTEM) project. *Drug and Alcohol Review* 2021;40(5):755-60. doi: https://doi.org/10.1111/dar.13272
18. Ferris J, PuljeviĆ C, Taylor N, et al. The impact of Queensland's Tackling Alcohol-Fuelled Violence Policy on nightlife and business trade. *Drug and Alcohol Review* 2021;40(5):746-54. doi: https://doi.org/10.1111/dar.13271
19. Puljević C, Carah N, Miller PG, et al. The impact of the Queensland Government's Tackling Alcohol-Fuelled Violence Policy on live music performances in Fortitude Valley, Brisbane, Australia. *Drug and Alcohol Review* 2021;40(5):738-45. doi: https://doi.org/10.1111/dar.13274
20. de Andrade D, Coomber K, Ferris J, et al. The impact of changes to alcohol policy on patron drinking behaviours in Queensland. *Drug and Alcohol Review* 2021;40(5):728-37. doi: https://doi.org/10.1111/dar.13299
21. Coomber K, de Andrade D, Puljević C, et al. The impact of liquor legislation changes on police-recorded serious assault in Queensland, Australia. *Drug and Alcohol Review* 2021;40(5):717-27. doi: https://doi.org/10.1111/dar.13181
22. de Andrade D, Coomber K, Livingston M, et al. The impact of late-night alcohol restrictions on ambulance call-outs in entertainment precincts. *Drug and Alcohol Review* 2021;40(5):708-16. doi: https://doi.org/10.1111/dar.13308
23. Livingston M, Coomber K, de Andrade D, et al. Assessing the impact of Queensland's late-night alcohol restrictions using health system data. *Drug and Alcohol Review* 2021;40(5):698-707. doi: https://doi.org/10.1111/dar.13182
24. Miller PG, Coomber K, de Andrade D, et al. Queensland Alcohol-related violence and Night-time Economy Monitoring (QUANTEM): Rationale and overview. *Drug and Alcohol Review* 2021;40(5):693-97. doi: https://doi.org/10.1111/dar.13270
25. Burton R, Henn C, Lavoie D, et al. A rapid evidence review of the effectiveness and cost-effectiveness of alcohol control policies: an English perspective. *The Lancet* 2017;389(10078):1558-80. doi: 10.1016/S0140-6736(16)32420-5
26. Pliakas T, Egan M, Gibbons J, et al. Increasing powers to reject licences to sell alcohol: Impacts on availability, sales and behavioural outcomes from a novel natural experiment evaluation. *Preventive medicine* 2018;116 doi: 10.1016/j.ypmed.2018.09.010
27. Hadfield P, Lister S, Traynor P. 'This town'sa different town today': policing and regulating the night-time economy. *Criminology and Criminal Justice* 2009;9(4):465-85. doi: https://doi.org/10.1177/1748895809343409
28. Roberts M. From 'creative city'to 'no-go areas' - the expansion of the night-time economy in British town and city centres. *Cities* 2006;23(5):331-38. doi: https://doi.org/10.1016/j.cities.2006.05.001
29. Hayward K, Hobbs D. Beyond the binge in 'booze Britain': market-led liminalization and the spectacle of binge drinking. *Br J Sociol* 2007;58(3):437-56. doi: https://doi.org/10.1111/j.1468-4446.2007.00159.x
30. Nicholls J. Time for reform? Alcohol policy and cultural change in England since 2000. *British Politics* 2012;7(3):250-71.
31. Nicholls J. Public health and alcohol licensing in the UK: challenges, opportunities, and implications for policy and practice. *Contemp Drug Probl* 2015;42(2):87-105. doi: https://doi.org/10.1177/0091450915579875
32. Martineau F, Graff H, Mitchell C, et al. Responsibility without legal authority? Tackling alcohol-related health harms through licensing and planning policy in local government. *J Public Health* 2014;36(3):435-42. doi: https://doi.org/10.1093/pubmed/fdt079

For peer review only - http://bmjopen.bmj.com/site/about/guidelines.xhtml

33. Home Office. Police Reform and Social Responsibility Act 2011: The Late Night Levy. London: Home Office, 2011.
34. Home Office. Impact assessment for the alcohol neasures in the Police Reform and Social Responsbility Act. London: Home Office, 2011.
35. Home Office. Amended guidance on the late night levy. London: Home Office, 2015.
36. Carey G, Malbon E, Carey N, et al. Systems science and systems thinking for public health: a systematic review of the field. *BMJ Open* 2015;5(12):e009002. doi: http://dx.doi.org/10.1136/bmjopen-2015-009002
37. Bagnall A-M, Radley D, Jones R, et al. Whole systems approaches to obesity and other complex public health challenges: a systematic review. *BMC Public Health* 2019;19(1):8.
38. Rutter H, Savona N, Glonti K, et al. The need for a complex systems model of evidence for public health. *Lancet* 2017;390(10112):2602-04. doi: https://doi.org/10.1016/S0140-6736(17)31267-9
39. Meadows DH, Wright D. Thinking in Systems: A Primer. White River Junction, Vermont: Chelsea Green Publishing 2008.
40. Cilliers P. Boundaries, hierarchies and networks in complex systems. *International Journal of Innovation Management* 2001;5(02):135-47.
41. Finegood DT, Johnston LM, Steinberg M, et al. Complexity, systems thinking and health behavior change. In: Kahan S, Green L, Gielen A, et al., eds. Health Behavior Change in Populations. Baltimore, MD: Johns Hopkins University Press 2014:435-58.
42. Boulton J, Allen P, Bowman C. Embracing Complexity: Strategic Perspectives for an Age of Turbulence. Oxford: Oxford University Press 2015.
43. Walton M. Setting the context for using complexity theory in evaluation: boundaries, governance and utilisation. *Evid Policy* 2016;12(1):73-89. doi: https://doi.org/10.1332/174426415X14298726247211
44. Hawe P, Bond L, Butler H. Knowledge theories can inform evaluation practice: what can a complexity lens add? *New Dir Eval* 2009;2009(124):89-100. doi: https://doi.org/10.1002/ev.316
45. Walton M. Applying complexity theory: a review to inform evaluation design. *Eval Program Plann* 2014;45:119-26. doi: https://doi.org/10.1016/j.evalprogplan.2014.04.002
46. Rickles D, Hawe P, Shiell A. A simple guide to chaos and complexity. *J Epidemiol Community Health* 2007;61(11):933-37. doi: http://dx.doi.org/10.1136/jech.2006.054254
47. Buijs J-M, Eshuis J, Byrne D. Approaches to researching complexity in public management. In: Teisman G, van Buuren A, Gerrits L, eds. Managing complex governance systems: dynamics, self-organization and coevolution in public investments. London: Routledge 2009:37-55.
48. Eppel E, Matheson A, Walton M. Applying complexity theory to New Zealand public policy: principles for practice. *Policy Quarterly* 2011;7(1):48-55. doi: https://doi.org/10.26686/pq.v7i1.4369
49. Kania A, Patel AB, Roy A, et al. Capturing the complexity of evaluations of health promotion interventions: a scoping review. *Can J Program Eval* 2012;27(1):65-91.
50. Byrne D, Callaghan G. Complexity Theory and the Social Sciences: The State of the Art. Abingdon: Routledge 2014.
51. Gates EF. Making sense of the emerging conversation in evaluation about systems thinking and complexity science. *Eval Program Plann* 2016;59:62-73. doi: https://doi.org/10.1016/j.evalprogplan.2016.08.004
52. Walton M. Expert views on applying complexity theory in evaluation: opportunities and barriers. *Evaluation* 2016;22(4):410-23. doi: https://doi.org/10.1177/1356389016667890
53. Gatrell AC. Complexity theory and geographies of health: a critical assessment. *Soc Sci Med* 2005;60(12):2661-71. doi: https://doi.org/10.1016/j.socscimed.2004.11.002
54. Bambra C, Smith KE, Pearce J. Scaling up: the politics of health and place. *Soc Sci Med* 2019;232:36-42. doi: https://doi.org/10.1016/j.socscimed.2019.04.036

CONFIDENTIAL
For peer review only

55. McGill E, Petticrew M, Marks D, et al. Applying a complex systems perspective to alcohol consumption and the prevention of alcohol-related harms in the 21st century: a scoping review. *Addiction* 2020 doi: https://doi.org/10.1111/add.15341
56. Egan M, McGill E, Penney T, et al. NIHR SPHR Guidance on systems approaches to local public health evaluation. Part 1: Introducing systems thinking. London: National Institute for Health Research School for Public Health Research, 2019.
57. McGill E, Marks D, Er V, et al. Qualitative process evaluation from a complex systems perspective: a systematic review and framework for public health evaluators. *PLoS Med* 2020;17(11):e1003368. doi: https://doi.org/10.1371/journal.pmed.1003368
58. Makleff S, Billowitz M, Garduño J, et al. Applying a complex adaptive systems approach to the evaluation of a school-based intervention for intimate partner violence prevention in Mexico. *Health Policy Plan* 2020;35(8):993-1002. doi: https://doi.org/10.1093/heapol/czaa067
59. Mintrop R, Pryor L, Ordenes M. A complex adaptive system approach to evaluation: application to a pay-for-performance program in the USA. *Educ Assess Eval Account* 2018;30(3):285-312. doi: https://doi.org/10.1007/s11092-018-9276-6
60. Hawe P, Shiell A, Riley T. Theorising interventions as events in systems. *Am J Comm Psych* 2009;43(3-4):267-76.
61. Moore GF, Audrey S, Barker M, et al. Process evaluation of complex interventions: Medical Research Council guidance. *BMJ* 2015;350:h1258. doi: https://doi.org/10.1136/bmj.h1258
62. Visual Understanding Environment (VUE) [program]. Medford, MA: Tufts University 2020.
63. NVivo Qualitative Data Analysis Software, Version 12 [program], 2012.
64. Humphreys DK, Eisner MP. Do flexible alcohol trading hours reduce violence? A theory-based natural experiment in alcohol policy. *Social Science & Medicine* 2014;102:1-9. doi: https://doi.org/10.1016/j.socscimed.2013.11.038
65. Humphreys DK, Eisner MP, Wiebe DJ. Evaluating the impact of flexible alcohol trading hours on violence: an interrupted time series analysis. *PLoS One* 2013;8(2):e55581. doi: 10.1371/journal.pone.0055581 [published Online First: 2013/03/05]
66. Rehm J, Gmel GE, Sr., Gmel G, et al. The relationship between different dimensions of alcohol use and the burden of disease-an update. *Addiction (Abingdon, England)* 2017;112(6):968-1001. doi: 10.1111/add.13757 [published Online First: 2017/02/22]
67. McGill E, Egan M, Petticrew M, et al. Trading quality for relevance: non-health decision-makers' use of evidence on the social determinants of health. *BMJ open* 2015;5(4):e007053. doi: 10.1136/bmjopen-2014-007053
68. Rinaldi C, van Schalkwyk MC, Egan M, et al. A Framing Analysis of Consultation Submissions on the WHO Global Strategy to Reduce the Harmful Use of Alcohol: Values and Interests. *International Journal of Health Policy and Management* 2021:-. doi: 10.34172/ijhpm.2021.68
69. License A, Edwards A, Bevan T. Measuring the Australian Night Time Economy 2016-17. Melbourne: Ingenium Research Pty Ltd., 2018.
70. Munro V. Redbridge delays introducing tax on night-time alcohol sales. *Ilford Recorder* 2020 Sept 10 2020. https://www.ilfordrecorder.co.uk/news/late-night-alcohol-levy-delayed-3268374 (accessed Feb 2022).
71. Southampton City Council. Southampton Late Night Levy will end on 31 March 2021 2020 [Available from: https://www.southampton.gov.uk/business-licensing/licensing/licensing-act-2003/late-night-levy accessed Feb 03 2022.
72. Smith SW, Atkin CK, Roznowski J. Are "Drink Responsibly" Alcohol Campaigns Strategically Ambiguous? *Health Communication* 2006;20(1):1-11. doi: 10.1207/s15327027hc2001_1
73. Pettigrew S, Biagioni N, Daube M, et al. Reverse engineering a ‘responsible drinking’ campaign to assess strategic intent. *Addiction (Abingdon, England)* 2016;111(6):1107-13. doi: https://doi.org/10.1111/add.13296

For peer review only - http://bmjopen.bmj.com/site/about/guidelines.xhtml

74. Petticrew M, Fitzgerald N, Durand MA, et al. Diageo's 'Stop Out of Control Drinking' Campaign in Ireland: An Analysis. *PLoS One* 2016;11(9):e0160379. doi: 10.1371/journal.pone.0160379
75. Mialon M, McCambridge J. Alcohol industry corporate social responsibility initiatives and harmful drinking: a systematic review. *European Journal of Public Health* 2018;28(4):664-73. doi: 10.1093/eurpub/cky065
76. McCambridge J, Mialon M, Hawkins B. Alcohol industry involvement in policymaking: a systematic review. *Addiction (Abingdon, England)* 2018;113(9):1571-84. doi: https://doi.org/10.1111/add.14216
77. Knai C, Petticrew M, Durand MA, et al. Are the Public Health Responsibility Deal alcohol pledges likely to improve public health? An evidence synthesis. *Addiction (Abingdon, England)* 2015;110(8):1232-46. doi: https://doi.org/10.1111/add.12855
78. Babor TF, Robaina K, Brown K, et al. Is the alcohol industry doing well by 'doing good'? Findings from a content analysis of the alcohol industry's actions to reduce harmful drinking. *BMJ Open* 2018;8(10):e024325. doi: 10.1136/bmjopen-2018-024325
79. Petticrew M, Fitzgerald N, Maani N, et al. Responsible Drinking, Conflicts of Interest, and the Elephant in the Room: A Commentary on A Scoping Review of "Responsible Drinking" Interventions by Gray, Williams & Shaffer (2020). *Health Communication* 2021;36(2):257-59. doi: 10.1080/10410236.2020.1827541
80. McGill E, Er V, Penney T, et al. Evaluation of public health interventions from a complex systems perspective: a research methods review. *Soc Sci Med* 2021;272:113697. doi: https://doi.org/10.1016/j.socscimed.2021.113697
81. Foster J, Charalambides L. The Licensing Act (2003): its uses and abuses 10 years on. London: Institute of Alcohol Studies, 2016.
82. Foster J, Harrison A, Brown K, et al. Anytime, anyplace, anywhere? Addressing physical availability of alcohol in Australia and the UK. London and Canberra: Institute for Alcohol Studies and the Foundation for Alcohol Research and Education, 2017.
83. Verweij S, Gerrits LM. Understanding and researching complexity with qualitative comparative analysis: Evaluating transportation infrastructure projects. *Evaluation* 2013;19(1):40-55.
84. Baugh Littlejohns L, Baum F, Lawless A, et al. The value of a causal loop diagram in exploring the complex interplay of factors that influence health promotion in a multisectoral health system in Australia. *Health Res Policy Syst* 2018;16(1):126. doi: https://doi.org/10.1186/s12961-018-0394-x
85. Mowles C. Complex, but not quite complex enough: The turn to the complexity sciences in evaluation scholarship. *Evaluation* 2014;20(2):160-75.
86. Ulrich W, Reynolds M. Critical systems heuristics: the idea and practice of boundary critique. In: Reynolds M, Holwell S, eds. Systems approaches to making change: a practical guide. London: Springer 2020:255-306.
87. Home Office. Policing and Crime Bill: Changes to the Late Night Levy Impact Assessment. London: Home Office, 2016.
88. Kolvin P, Eames C. Late night levy in lockdown. *Morning Advertiser* 2020 Apr 29.

For peer review only - http://bmjopen.bmj.com/site/about/guidelines.xhtml

Figure 1: Hypothesis 1

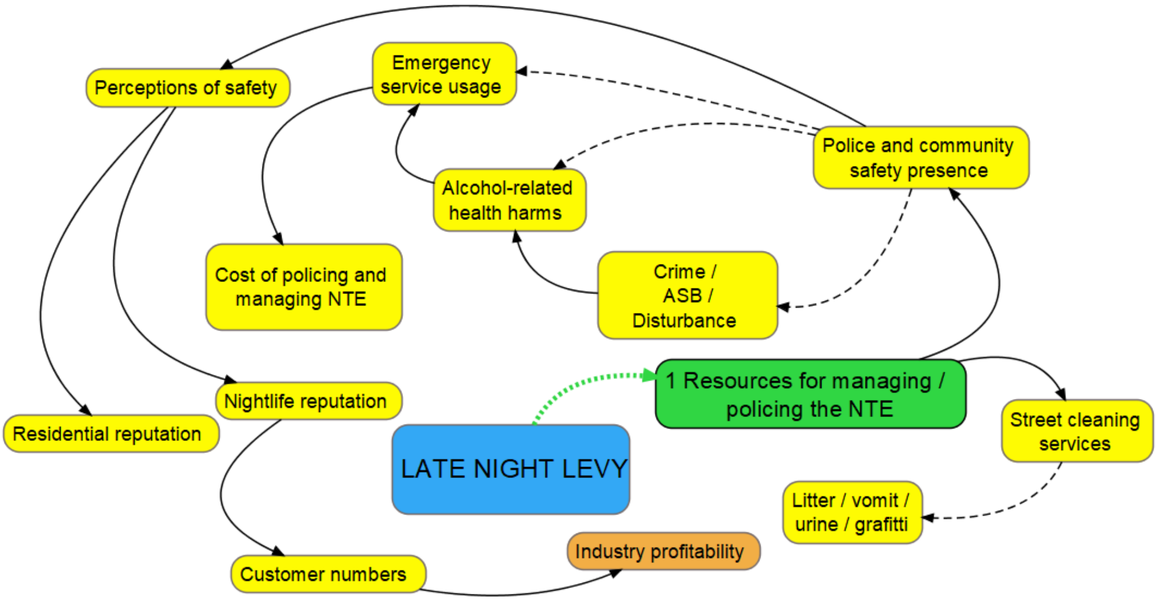

For peer review only

Figure 2: Hypothesis 2

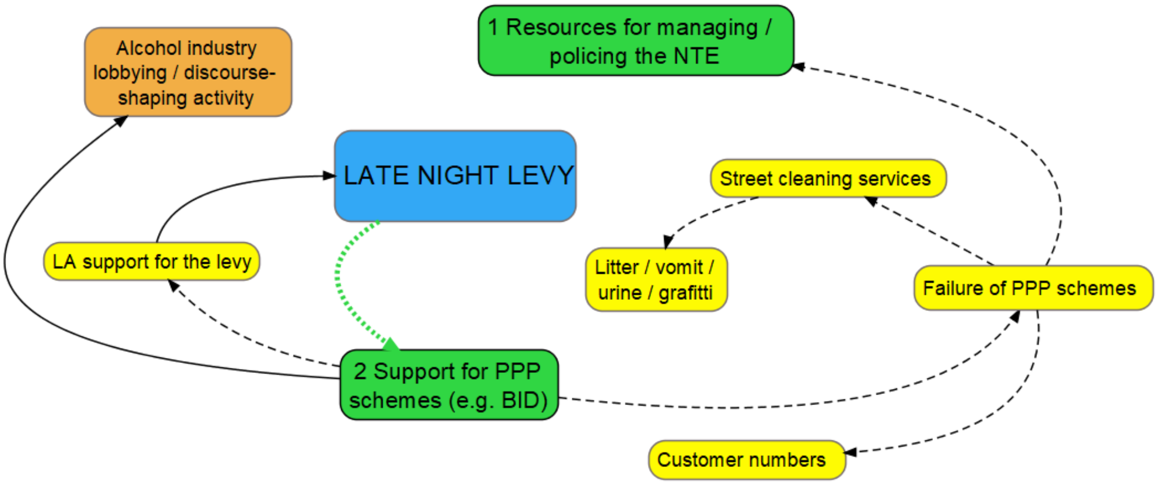

For peer review only

Figure 3: Hypotheses 3 and 4

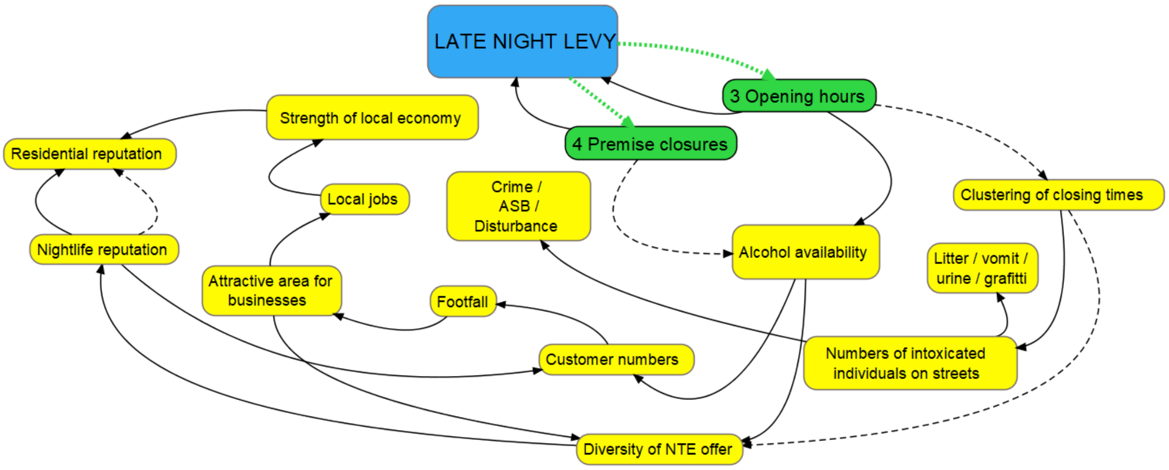

Supplementary Material 1: Reporting Checklist

Standards for Reporting Qualitative Research (SRQR)*

http://www.equator-network.org/reporting-guidelines/srqr/

Page no(s).

Title and abstract

**Title** - Concise description of the nature and topic of the study Identifying the study as qualitative or indicating the approach (e.g., ethnography, grounded theory) or data collection methods (e.g., interview, focus group) is recommended	First page
**Abstract** - Summary of key elements of the study using the abstract format of the intended publication; typically includes background, purpose, methods, results, and conclusions	First page

Introduction

**Problem formulation** - Description and significance of the problem/phenomenon studied; review of relevant theory and empirical work; problem statement	Introduction, paraphs 1-2
**Purpose or research question** - Purpose of the study and specific objectives or questions	Introduction, final paragraph

Methods

**Qualitative approach and research paradigm** - Qualitative approach (e.g., ethnography, grounded theory, case study, phenomenology, narrative research) and guiding theory if appropriate; identifying the research paradigm (e.g., postpositivist, constructivist/ interpretivist) is also recommended; rationale**	Methods, paragraph 1
**Researcher characteristics and reflexivity** - Researchers' characteristics that may influence the research, including personal attributes, qualifications/experience, relationship with participants, assumptions, and/or presuppositions; potential or actual interaction between researchers' characteristics and the research questions, approach, methods, results, and/or transferability	Methods, paragraph 6
**Context** - Setting/site and salient contextual factors; rationale**	Introduction, paragraph 1; Methods paragraph 2
**Sampling strategy** - How and why research participants, documents, or events were selected; criteria for deciding when no further sampling was necessary (e.g., sampling saturation); rationale**	Methods, paragraphs 3-10; Tables 3 and 4
**Ethical issues pertaining to human subjects** - Documentation of approval by an appropriate ethics review board and participant consent, or explanation for lack thereof; other confidentiality and data security issues	Methods, paragraph 11

For peer review only

**Data collection methods** - Types of data collected; details of data collection procedures including (as appropriate) start and stop dates of data collection and analysis, iterative process, triangulation of sources/methods, and modification of procedures in response to evolving study findings; rationale**	Methods, paragraphs 3-10
**Data collection instruments and technologies** - Description of instruments (e.g., interview guides, questionnaires) and devices (e.g., audio recorders) used for data collection; if/how the instrument(s) changed over the course of the study	Methods, paragraphs 6-10; Supplementary material 2
**Units of study** - Number and relevant characteristics of participants, documents, or events included in the study; level of participation (could be reported in results)	Tables 3 and 4
**Data processing** - Methods for processing data prior to and during analysis, including transcription, data entry, data management and security, verification of data integrity, data coding, and anonymization/de-identification of excerpts	Methods, paragraphs 13-15
**Data analysis** - Process by which inferences, themes, etc., were identified and developed, including the researchers involved in data analysis; usually references a specific paradigm or approach; rationale**	Methods, paragraphs 13-15; Supplementary Material 3
**Techniques to enhance trustworthiness** - Techniques to enhance trustworthiness and credibility of data analysis (e.g., member checking, audit trail, triangulation); rationale**	Methods, paragraphs 14-15; Supplementary Material 3

Results/findings

**Synthesis and interpretation** - Main findings (e.g., interpretations, inferences, and themes); might include development of a theory or model, or integration with prior research or theory	Results
**Links to empirical data** - Evidence (e.g., quotes, field notes, text excerpts, photographs) to substantiate analytic findings	Results

Discussion

**Integration with prior work, implications, transferability, and contribution(s) to the field -** Short summary of main findings; explanation of how findings and conclusions connect to, support, elaborate on, or challenge conclusions of earlier scholarship; discussion of scope of application/generalizability; identification of unique contribution(s) to scholarship in a discipline or field	Discussion, paragraphs 1-10
**Limitations** - Trustworthiness and limitations of findings	Discussion, paragraphs 11-14

Other

**Conflicts of interest** - Potential sources of influence or perceived influence on study conduct and conclusions; how these were managed	Conflict of interest statement

For peer review only

**Funding** - Sources of funding and other support; role of funders in data collection, interpretation, and reporting	Funding statement

*The authors created the SRQR by searching the literature to identify guidelines, reporting standards, and critical appraisal criteria for qualitative research; reviewing the reference lists of retrieved sources; and contacting experts to gain feedback. The SRQR aims to improve the transparency of all aspects of qualitative research by providing clear standards for reporting qualitative research.

**The rationale should briefly discuss the justification for choosing that theory, approach, method, or technique rather than other options available, the assumptions and limitations implicit in those choices, and how those choices influence study conclusions and transferability. As appropriate, the rationale for several items might be discussed together.

Reference:
O'Brien BC, Harris IB, Beckman TJ, Reed DA, Cook DA. **Standards for reporting qualitative research: a synthesis of recommendations.** *Academic Medicine*, Vol. 89, No. 9 / Sept 2014 DOI: 10.1097/ACM.0000000000000388

For peer review only

Supplementary Material 2: Data collection instruments

Interview topic guide: service providers (community safety and police)

1. Introduction:

- Study introduction, confidentiality, audio recording
- Any questions?

2 Can you tell me a bit about [*organisation/team*]? How do you operate?
Probe around: interaction with other parts of the organisation; interaction with other services; intelligence generation

3. As an [position], what does your role entail?
Probe around: typical shift; generating taskings; usual activities

4. Can you tell me a bit more about the people you tend to work with or encounter on patrols?
Probe around: drinkers in particular – drinking habits, purchasing habits, location of drinking, behaviours associated with drinking; co-occurrence of drinking and other behaviours

5. From your perspective, what are the major alcohol-related challenges in [LA]?
Probe around: specific population groups; different types of venues; specific venues; specific areas of LA; specific drinks

- **How do you think [LA] compares to other areas?**

5. Can you tell me about the Late Night Levy?

- **How did the levy come about in [LA]?**
  - *Probe around:* supporters/opponents, who drove the implementation, etc.
- **What is the aim of the levy?**
  - *Probe around:* targeting of intervention (drinkers, retailers)

6. How has the levy changed the services you provide?
Probe around: working with other services, deployment of resources; focus of shifts/taskings

7. Thinking about the people you encounter and the venues you engage with, do you think the initiative has had any impacts on them?
For drinkers, probe around: consumption and behaviour (intoxication to ASB/crime)
For retailers: opening hours, engagement with police/council/community safety; management practices

8. **Have you seen any other impacts of the initiative, either health or non-health related?**
Probe around: why these have occurred

10. Over the past few years, have you been aware of, or involved in, any other new alcohol initiatives or services?
Probe around: interaction with the levy, impacts on retailers and drinkers

11. Wrap up and end

For peer review only

Interview topic guide: drinkers

1. To start off, can you tell me a bit about yourself?
Probe around: age, work, resident/visitor

2. What do you like about going out/drinking in [LA]?
Probe around: usual places they go and why; what they drink; who they are usually with; drinking at home vs. out

3. Have you noticed any changes in [LA] and the places to go out here?
Probe around: types of places, types of people drinking in them, reasons for changes

- **Are there any changes you want to see here to make [LA] a better place to go out?**

4. Have changed where you like to go out or where you buy drinks over the past few years?
Probe around: changes to drinking patterns – locations, amount, types of drink, time they drink, etc.

5. Do you think there are any problems with alcohol in [LA]?
Probe around: specific types of people, parts of the LA, specific venues, etc.

- **Have you ever been moved on whilst drinking on the streets or come into contact with police? Ever been ejected from a premise?**

6. [LA] has a Late Night Levy in place – have you heard about it?

- If yes, **what do you think the levy is trying to do?**
    - *Probe around:* aims, reason to bring it in, if they have seen any changes
- If not, explain levy: **what do you think about an initiative like that?**
    - *Probe around:* community safety/police presence, venue opening times, types of venues in area, prices, what changes in might bring in, etc

7. Have you noticed any changes in LA's night time environment?
Probe around: times places are open; drink offers, how venues are managed – e.g. door staff, tolerance for rowdiness/fighting/etc, police on street, community safety on street, etc

8. Wrap-up and end

For peer review only

Observation template: patrols

Fieldworker:
Patrol Officers:
Date:
Patrol time and length:

Pre-patrol briefing (if applicable):

- Specific aims of the patrol
- Areas to be patrolled
- Specific venues/locations to visit
- Previous incidents requiring follow-up action

How the patrol operates:

- Area of patrol
- Means of patrol (on foot, in car, etc.)
- How officers determine how to focus their attention (walk around and spot incidents, respond to calls, etc.)

Incidents encountered: for each incident with drinking involved (or suspected):

- Location of incident
- Name of premise (if applicable)
- Individual or group encountered
- Characteristics of the individual/group (age, gender, dress, etc.)
- Behaviour of individual/group (drunk and disorderly, aggressive, subdued, unconscious, etc.)
- How patrol officers interact with individual/group encountered:
- Individual/group reaction to intervention
- Outcome of intervention
- Other services involved (police, ambulance premise staff, etc.)
- Patrol officers' interpretation of incident

Non-drinking incidents encountered:

- Types of incidents
- Types of individuals/groups involved

Patrol staff:

- Characteristics (age, gender, physical characteristics)
- Length of time working on this patrol
- Types of individuals/groups that attract their attention
- Types of individuals/groups that do not attract their attention
- Impressions of problem drinking (types of people, areas of LA, types of premises, specific venues, specific drinks) and evolution over time
- Perceptions of LNL (aim, achieving aim? how? changes in how the service has been delivered? Relationships with other services; public, venues)
- Knowledge and impressions of other alcohol interventions: Cumulative Impact Policies, Reducing the Strength – others; interaction with the levy

Researcher reflexivity

For peer review only

Supplementary Material 3: Coding framework

This supplementary material file presents our coding framework, which is described in tabular form and visually represented in the form of a map of the local system. The map is a synthesis of stakeholder perceptions and was used to generate hypotheses that the second phase of the evaluation examines in more detail. The map is comprised of a range of variables that emerged from the analysis of local and national data generated and collected in Phase 1 of the evaluation. To identify and define variables of interest, two researchers (EM and ME) independently generated and subsequently reconciled a list of variables that, from the perspective of system stakeholders, characterise the system of inquiry. A description of each variable is provided in the table, along with the other system variables that system stakeholders perceived each variable to be directly related to and/or from. The final column in the table provides an excerpt of data to illustrate each variable. The figure depicts these variables visually on a system map. Each variable is represented by a bubble; the lines represent relationships to other system variables and whether the two variables are positively (solid line) or inversely (dashed line) related to each other. The system represented in the diagram is bounded so that it contains the elements operating within the geographical area of the LA. However, the local system is a complex system and therefore these boundaries should be considered 'open'.

For peer review only

Table 1: System variables and relationships within the local system

System	Variable	Description	Connected to/from (positive or inverse relationship)	Example(s) (source)
National	National support for NTE regulation	The degree to which national policymakers support the introduction and extension of regulation in the NTE.	To: LA powers to regulate NTE and alcohol licensing (+); societal alcohol-related economic/health harms (+)<br><br>From: alcohol-industry lobbying / discourse-shaping activity (-)	"The Government intends to rebalance the licensing regime to enable local 'Licensing Authorities' (LAs) and the police to clamp down on alcohol-related crime and disorder, particularly late at night; to allow wider considerations and the views of local communities to be taken into account in licensing decisions; to protect children from the harm of alcohol; and introduce a late night levy to help pay for other costs caused by late-night drinking." (Impact assessment for Police Reform and Social Responsibility Bill 2010)
	LA powers to regulate NTE and alcohol licensing	The specific powers available to LAs to regulate the NTE and control the provision of alcohol through alcohol licensing powers, such as the LNLs, CIPs, and EMROs.	To: LA support for the levy (+); alcohol outlet density (-)<br><br>From: national support for NTE regulation (+)	"The late night levy is a discretionary power enabling licensing authorities in England and Wales to raise a contribution towards policing the late-night economy from holders of premises licences or club premises certificates." (House of Commons, Late Night Levy Briefing, 2015)
	Societal alcohol-related economic and health harms	The societal harms associated with alcohol, including the associated healthcare costs, productivity losses and population-level health harms.	To: national support for NTE regulation (+)<br><br>From: alcohol consumption (+)	"Alcohol misuse also costs the United Kingdom economy an estimated £7.3 billion a year in lost productivity and the National Health Service in England an estimated £3.5 billion a year. In England, over 15,000 people die from alcohol-related illnesses each year." (Next steps on delivering the Government's Alcohol Strategy, 2013)
	Alcohol industry lobbying / discourse-shaping activity	Activities the alcohol industry engages in to lobby for and frame debates in terms favourable to their corporate interests.	To: industry profitability (+) ; national support for NTE regulation (-)<br><br>From: support for PPP schemes (+)	"We believe that the Council should maintain its current voluntary best practice approach which is delivering real results and crucially is focused on eliminating the source of the problems rather than simply paying for any clean up.[…] This is in line with the National Alcohol Strategy which states that targeted action taken voluntarily by pubs and clubs themselves is most effective in curbing irresponsible drinking and associated drunken violence. The Home Secretary, Theresa May, in publishing the strategy suggested that a legislative approach, either national or local, was a 'sledgehammer' which all too often misses its target and that a partnership approach was more effective." (Consultation response, trade organisation representing on-licence premises).
National	Industry profitability	The financial gains realised by the alcohol industry.	To: business rates/licence fees (+)<br><br>From: alcohol-industry lobbying / discourse-	"[The LNL] will impose a significant further cost burden on the hospitality industry in the [local] area when the overall costs the industry must pay whether food, drink, labour and taxes contribute to rise and customers' real incomes shrink impacting on profitability". (Consultation response, operator of managed pubs).

For peer review only

System	Variable	Description	Connected to/from (positive or inverse relationship)	Example(s) (source)
			shaping activity (+); customer numbers (+)	
Local	Alcohol outlet density	Concentration of premises selling alcohol for consumption on or off the premises.	To: alcohol consumption (+)<br><br>From: LA powers to regulate the NTE and alcohol licensing (-); mixed land use (+)	"The number of licenced premises continues to grow rapidly so that [LA] has one of the highest densities of pubs, bars, clubs and off licences in the country and second highest in London after the City of Westminster." (Licensing Policy 2013-2017)<br><br>"I: So from your perspective, what would you say are the major kind of alcohol related challenges in [LA]?<br>R: *There are too many licensed premises."* (Interview, Police licensing officer)
	Alcohol consumption	The amount of alcohol an individual consumes. Can be measured in the context of a single drinking event, or to describe patterns of consumption.	To: crime / ASB / disturbance (+); alcohol-related health harms (+)<br><br>From: alcohol outlet density (+) ; alcohol availability (+)	"Drinking above recommended maximum limits has become much more common over the past two decades, particularly among younger and middle aged men and women of all social groups. This trend is mirrored in [LA]. […] The effects of the increase in alcohol consumption seen over the past two decades are now being seen in the significant increases in alcohol-related attendances and admissions in the NHS." (Licensing Policy 2011-2014)<br><br>"Alcohol consumption is increasingly identified as a major factor behind violent crime and disorder in the borough with serious consequences to victims, businesses and local communities." (Licensing Policy 2013-2017)
	Alcohol-related health harms	Individual health harms experienced as a result of alcohol consumption.	To: LA support for the levy (+); emergency service usage (+)<br><br>From: alcohol consumption (+); crime / ASB / disturbance (+); police and community safety presence (-)	"In reviewing our Licensing Policy we have been mindful that [LA's] residents suffer from high levels of alcohol-related ill health and early deaths." (Licensing Policy 2013-2017)<br><br>"Studies assessing the effectiveness of limiting the density of alcohol outlets showed greater alcohol outlet density to be associated with increased alcohol consumption and harms including injury, violence, crime and medical harm. Small numbers of concentrated problematic nightlife venues often cause a large proportion of alcohol-related harm, violence and injuries in city centres." (Public Health Report 2012)
Local	Mixed land use	The degree to which areas within the LA are mixed residential and commercial.	To: residential reputation (-); alcohol outlet density (+)<br><br>From: population change (+)	"[LA name] is a densely occupied area, with no clear delineation between residential and commercial premises and the Licensing Authority will need to carefully balance the conflicting needs of residents, patrons and businesses in relation to the introduction of flexible opening hours for the sale and supply of alcohol and late night refreshments." (Licensing Policy 2011-2014)

For peer review only - http://bmjopen.bmj.com/site/about/guidelines.xhtml

System	Variable	Description	Connected to/from (positive or inverse relationship)	Example(s) (source)
	Population change	The demographic characteristics of the local population and its rate of change.	To: mixed land use (+)<br><br>From: residential reputation (+)	"[LA], however, is undergoing a process of rapid change and this is likely to continue. The number of people living here has grown from 178,000 in 2001 to an estimated 199,130 in 2010. One of the reasons for this is the increase in the number of young adults who are moving into inner London, and starting families. […] This has turned many parts of the borough, which were previously exclusively commercial, into mixed-use hubs incorporating commercial and residential premises in very close proximity." (Licensing Policy 2011-2014)
	Residential reputation	The extent to which the local area is viewed as a desirable place to live.	To: population change (+); council tax (+)<br><br>From: perceptions of safety (+); mixed land use (-); nightlife reputation (+/-); strength of the local economy (+)	"I spoke to the public health lead for alcohol in [neighbouring LA] last week and he was saying this, exactly. […] Having all those bars, having all those people drinking, that's what they call regeneration, whereas in [this LA], my impression what they call regeneration is a good place to live, not a good place to party." (Interview, Public Health specialist)<br><br>"[LA name's] nightlife is marvellous and one of the reasons I love being a resident here." (Consultation response, resident)
	Nightlife reputation	The extent to which a local area is viewed as a desirable place to go out.	To: residential reputation (+/-); customer numbers (+)<br><br>From: perceptions of safety (+); diversity of NTE offer(+)	[Participant] almost always goes out in [LA] when he goes out. He likes the variety of places and that you can get fairly cheap drinks and still be in Zone 1. (Excerpt from fieldnotes)<br><br>"[LA] has a reputation for its vibrant nightlife and the council, along with its partners the Metropolitan Police and London Fire Brigade recognise that many people are working hard to ensure that [LA]'s clubs, pubs and bars provide a safe and attractive venue for customers." (Licensing Policy 2011-2014)
	Cost of policing and managing the NTE	The resources required to manage and regulate the NTE; includes police, community safety, street cleaning, licensing and trading standards.	To: LA support for the levy (+)<br><br>From: emergency service usage (+); crime / ASB / disturbance (+)	"[LA] has a well established night-time economy that has continued to grow since the introduction of the Licensing Act in 2005. The number of late-night and 24 hours premises is high and they are spread across the borough. The costs of policing the late night economy are substantial." (Consultation response, Executive Member for Community Safety).

For peer review only

System	Variable	Description	Connected to/from (positive or inverse relationship)	Example(s) (source)
Local	LA support for the levy	The degree to which key stakeholders responsible for local area, support the need to introduce the levy; includes elected members and responsible authorities.	To: Late Night Levy<br><br>From: LA powers to regulate NTE and alcohol licensing (+); cost of policing and managing the NTE (+); alcohol-related health harms (+); strength of the local economy (-); litter / vomit / urine / graffiti (+); support for PPP schemes (-)	"The supporting statement for the consultation at Appendix A sets out why the Council, supported by the police, believes the levy is necessary and this is still felt to be strong and compelling." (Consultation response, Executive Member for Community Safety).
	Perceptions of safety	The extent to which individuals feel safe in their local environment; includes both residents and visitors perceptions.	To: residential reputation (+); nightlife reputation (+)<br><br>From: police and community safety presence (+)	"[LA] is a safe place to socialise, this is my perception and common among my going out friends" (Consultation response)<br>"I've lived in a lot of places – [LA] and, in particular, [popular NTE area] are hardly a war zone!" (Consultation response)
	Emergency service use	The use of emergency services, including ambulances, A&E and police.	To: cost of policing and managing the NTE (+)<br><br>From: police and community safety presence (-); alcohol-related health harms (+)	"Generally, as density of licensed premises in LA increases so does the number of ambulance callouts (shown on map) and also levels of alcohol-related crime." (Public Health Report, 2012)
	Crime / ASB/ Disturbance	The prevalence of crime, anti-social behaviour, disturbance and nuisance that is fuelled by alcohol consumption.	To: alcohol-related health harms (+); cost of policing and managing the NTE (+)<br><br>From: alcohol consumption (+); police and community safety presence (-); numbers of intoxicated individuals on the street (+)	"A comparison of alcohol related violence prior to deregulating licensing hours in 2004 with 2011 shows that in 2004 alcohol related crime peaked between the hours of 11pm and midnight. By 2011 the peak hours for alcohol related crime had expanded and shifted to midnight to 5am with a corresponding 600% increase in alcohol related crime." (Licensing Policy 2013-2017)<br>"I'm very pleased to see [name] council taking this initiative. I live in [area] which has more than enough pubs and clubs and where noisy customers in the street are a problem in the small hours." (Consultation response, resident)
Lo cal	Police and community safety presence	The visible presence of police and community safety officers on	To: emergency service usage (-); alcohol-related	"From October 2012, a new late night levy will empower local areas to make those businesses that sell alcohol late into the night contribute towards the

System	Variable	Description	Connected to/from (positive or inverse relationship)	Example(s) (source)
		the streets and in and around licensed premises.	health harms (-); crime / ASB / disturbance (-); perceptions of safety (+); litter / vomit / urine / graffiti (-)<br><br>From: resources for managing / policing the NTE (+)	cost of policing and wider local authority action. This will help enable visible and proactive policing at targeted locations where there are local needs." (The Government's Alcohol Strategy, 2012)<br><br>"The proposals we have for spending the levy are to: · have a uniformed presence patrolling at weekends to deal with enforcement issues, drunkenness, street urination, rowdy and nuisance behaviour and assistant to vulnerable individuals identified. · Provide additional policing to support operations targeting crime and disorder. […]" (LNL Consultation, 2013)
	Resources for managing / policing the NTE	The funds, staff and equipment needed to police and manage the NTE.	To: police and community safety presence (+) ; street cleaning services (+)<br><br>From: Late Night Levy; business rates / licence fees (+); council tax (+); failure of PPP schemes (-)	"The fees set for licensed premises have not increased since their introduction in 2005. During the intervening eight years, the demands for services have increased along with the number of premises opening late and it is now necessary to look to alternative ways of financing the services needed to manage the impacts." (LNL Consultation, 2013)<br><br>"[The Late Night Levy] could potentially curb some of the more dangerous behaviour and sales of alcohol, as well as provide the Council and Police with additional resources in recognition of the extra costs involved in policing, monitoring and cleaning the areas around premises with late alcohol licences." (Consultation response, political party)
	Business rates / license fees	The fees that premises pay to the LA; the business rate is based on the rateable value of the premise and the license fee is applicable for all premises with a license to sell alcohol for on- or off-premise consumption.	To: resources for managing / policing the NTE (+)<br><br>From: premise closures (-); industry profitability (+)	"Like I said, I don't want boarded up buildings. I can't have a whole area of [LA]. Because I live here, it's not good for the economy, it's not good for crime and disorder, you know. It's, it's not good, you know, it's not good for the Council, you know, we don't get business rates if premises are empty." (Interview, Licensing officer)<br><br>"The council and police should already be budgeting for such a problem. Bars and pubs already pay a licence fee to the council – what's that currently paying for?" (Consultation response, licence holder)
	Council tax	Tax paid by households to LA based on the value of the property to fund LA services.	To: resources for managing / policing the NTE (+)<br><br>From: residential reputation (+)	"Business rates/council tax is already astronomically high so I don't see why this shouldn't already be covered?" (Consultation response)
Local	Support for PPP schemes	The degree to which alcohol retailers and the alcohol industry support voluntary partnerships with the police and local authority, as well as other	To: failure of PPP schemes (+); alcohol-industry lobbying / discourse-shaping activity (+); LA support for the	"It penalises the hard work that many operators have undertaken in partnerships with the local authority and the police." (Consultation response, licence holder)<br><br>"[LA] has engaged in active partnership working with its licensed premises so as to ensure high standards of management that will prevent nuisance

For peer review only - http://bmjopen.bmj.com/site/about/guidelines.xhtml

System	Variable	Description	Connected to/from (positive or inverse relationship)	Example(s) (source)
		businesses, including the BIDs, PubWatch, Best Bar None, etc.	levy (-)<br><br>From: Late Night Levy	and crime. It has participated in Central Government initiatives, held training events and developed a number of best practice schemes, including the Community Alcohol Partnership programme […] There are six Pubwatches operating throughout the borough though this still sees only a small percentage of businesses actively engaging with the Council and police. Despite this engagement and the standards achieved within premises, [LA] continues to have a high level of alcohol related crime." (Consultation response, Executive Member for Community Safety).
	Failure of PPP schemes	The number of PPP schemes that licensees choose to no longer support.	To: street cleaning services (-); resources for managing / policing the NTE (-); customer numbers (-)<br><br>From: support for PPP schemes (-)	"It is also unrealistic in the current economic climate to expect operators to have funds available to support involvement in voluntary initiatives AND pay the late night levy. Where no discount is available to encourage participation in such schemes and to provide some financial relief for doing so, such schemes will wither on the vine as membership falls away." (Consultation response, Pub company)
	Opening hours	The hours until which a premise is licensed to sell alcohol.	To: alcohol availability (+); clustering of closing times (-); Late Night Levy<br><br>From: Late Night Levy	"[…] we understand that a large number of our members' business will choose to voluntarily restrict their hours. Generally, it is only dedicated late night businesses which will generate sufficient revenues after midnight to justify retaining licensing hours within the levy period. Many traditional pubs or restaurants will have later closing times but will not regularly use them and, as the levy is based on permission not use, will therefore voluntary relinquish them rather than face an additional cost." (Consultation response, trade association representing on-licence trade)
	Clustering of closing times	The degree to which premises close at the same time.	To: numbers of intoxicated individual on streets (+); diversity of NTE offer (-)<br><br>From: opening hours (-)	"One consequence of the levy is likely to be that a significant number of premises will reduce their hours to 12 midnight resulting in a return to a non staggered closing time culture, contrary to government policy." (Consultation response, supermarket chain).
**Local**	Number of intoxicated individuals on the street	The number of people out on the street who have been drinking.	To: crime / ASB / disturbance (+); litter / vomit / urine / graffiti (+)<br><br>From: clustering of closing times (+)	"The Council should also consider the risk of a defacto uniform terminal hour for premises arising as operators cut back their premises licences to cease at whenever the levy starts to bite. We have highlighted the actual experience of [LA with a levy] above. This will mean more customers on the street at the same time with resultant pressure on resources such as taxis, fast food outlets and policing." (Consultation response, Pub company)
	Alcohol availability	The ease and convenience of purchasing availability;	To: diversity of NTE offer (+); customer numbers	"Availability of and access to alcohol has an important influence on levels of alcohol consumption. Generally speaking, changes in the availability of

System	Variable	Description	Connected to/from (positive or inverse relationship)	Example(s) (source)
		availability is comprised of the density of licensed premises (physical availability), the hours in which alcohol is sold (temporal availability), and the cost of alcoholic beverages (economic availability).	(+); alcohol consumption (+)<br><br>From: opening hours (+); premise closures (-)	alcohol tend to be reflected sooner or later in changes in levels of alcohol consumption and alcohol-related harm." (Public Health Report, 2012)
	Premise closures	The number of alcohol-retailing premises who cease operating.	To: alcohol availability (-); business rates / licence fees (-); Late Night Levy<br><br>From: Late Night Levy	"There is a very real risk that [LA] Council will be responsible for premises closures, loss of jobs and income from rates (with higher outgoings in dealing with those people put out of work) from over-taxing those premises already struggling to cope with the burden they currently have." (Consultation response, Pub and bar operator)
	Street cleaning services	The services to keep streets clean, including clearing rubbish and recycling, washing away vomit and urine, and removing graffiti.	To: litter / vomit / urine / graffiti (-)<br><br>From: resources for managing / policing the NTE (+)	"The proposals we have for spending the levy are to: […] provide additional cleaning and service such as litter removal, graffiti removal and cleaning." (LNL Consultation, 2013)
	Litter/vomit/urine/graffiti	Quality of the physical environment; physical manifestations of incivility.	To: LA support for the levy (+)<br><br>From: street cleaning services (-); police and community safety presence (-); number of intoxicated individuals on the street (+)	"the Licensing Authority has seen an increase in concerns raised by local residents, Councillors and local businesses about the impact that the night time economy is having on the local environment in this area. Typical issues of concern include: • Public urination • Litter • Noise nuisance from patrons of licensed premises • Drug dealing • Thefts • Damage to property and vehicles • Obstruction of the public highway" (Licensing Policy, 2011-2014)<br><br>"For instance, a significant majority [of] litter on the streets at night comes from premises not licensed to tell alcohol at that time, such as takeaways and shops with late opening hours. It is illogical and unfair to make payment for cleaning up the sole responsibility of premises selling alcohol." (Consultation response, Pub company and brewer).
Local	Customer numbers	Number of patrons frequenting licensed premises.	To: footfall (+); industry profitability (+)<br><br>From: alcohol availability (+), nightlife reputation (+); failure of PPP schemes (-)	"Any reduction to its licensed hours made by venue operators will unquestionably have a detrimental impact on other local businesses in the area, including [LA's] many eateries, as there are likely to be fewer customers in the area for a shorter period of time, thus further affecting the financially lucrative late night economy." (Consultation response, licensee).
	Footfall	The number of individuals in the LA on a given night.	To: attractive area for businesses (+)	"We are bound to ask whether the Council has considered the consequences of closures not just to the licence holders in [LA] but the migration of customers into the neighbouring boroughs which will not only

For peer review only

System	Variable	Description	Connected to/from (positive or inverse relationship)	Example(s) (source)
			From: customer numbers (+)	affect the earlier evening trade but will also have the effect of damaging other businesses through lower footfall in that earlier evening." (Consultation response, Hospitality company)
	Attractive area for business	The degree to which an area is perceived as attractive for a business, includes number of residents and visitors, other types of establishments and 'friendliness' of Council to businesses.	To: local jobs (+); diversity of NTE offer (+)<br><br>From: footfall (+)	"The Council should consider it decision very carefully before [LA] becomes a place that becomes unattractive as a place to trade. [Pub company] will decide its future expansion and growth plans within London on the basis of how business friendly the incumbent Council is in any particular area." (Consultation response, pub company)
	Local jobs	The number of jobs in a local area, some of which are in the alcohol-retailing and hospitality sector.	To: strength of the local economy (+)<br><br>From: attractive area for businesses (+)	"This is an unfair tax on vulnerable businesses who provide wealth and jobs for the borough." (Consultation response, licence holder)<br><br>"But we're a poorer borough, we need people to have jobs, we don't want boarded up or empty premises". (Interview, Licensing officer)
	Strength of the local economy	The degree to which there are wealth generating activities in the LA. The NTE is one contributor to the local economy.	To: LA support for the levy (-); residential reputation (+)<br><br>From: local jobs (+)	"The council recognises that the entertainment and alcohol industry contributes to the borough by providing a variety of opportunities for entertainment as well as employment and career opportunities." (Licensing Policy 2011-2014)<br><br>"This is a significant additional cost for businesses to bear and it will affect profitability and viability for many. Some will choose to close earlier to avoid paying this, with knock on effects on turnover, GVA to the local economy and employment patterns." (Consultation response, trade association representing on-licence trade)
Local	Diversity of NTE offer	Different types of premises operating in the NTE, including: pubs, bars, clubs, restaurants, live music venues, off-licences, supermarkets, clubs, hotels and the characteristics of those premises (e.g. catering to different types of clientele).	To: nightlife reputation (+)<br><br>From: alcohol availability (+); clustering of closing times (-); attractive area for businesses (+)	"The council is keen to preserve a diverse mix of premises through the borough and wants to work with businesses, residents and partners through its Licensing Policy to resist the saturation of licensable premises of similar types within identified local areas." (Licensing Policy 2011-2014)<br><br>"We feel that there has not been enough research carried out into the benefits of a mixed and diverse night time economy in [LA} which could stagnate on the introduction of a levy. Premises where alcohol does not contribute significantly to profit but is offered after midnight may well close early. This will harm the diversity of offer, leaving only larger pubs and nightclubs willing to pay the levy." (Consultation response, pub company and brewer)

Abbreviations: A&E: Accident and Emergency; BID: Business Improvement District; CIP: Cumulative Impact Policy; EMRO: Early Morning Restriction Order; LA: Local Authority; LNL: Late Night Levy; NTE: night time economy; PPP: public-private partnership

For peer review only - http://bmjopen.bmj.com/site/about/guidelines.xhtml

Figure 1: system map

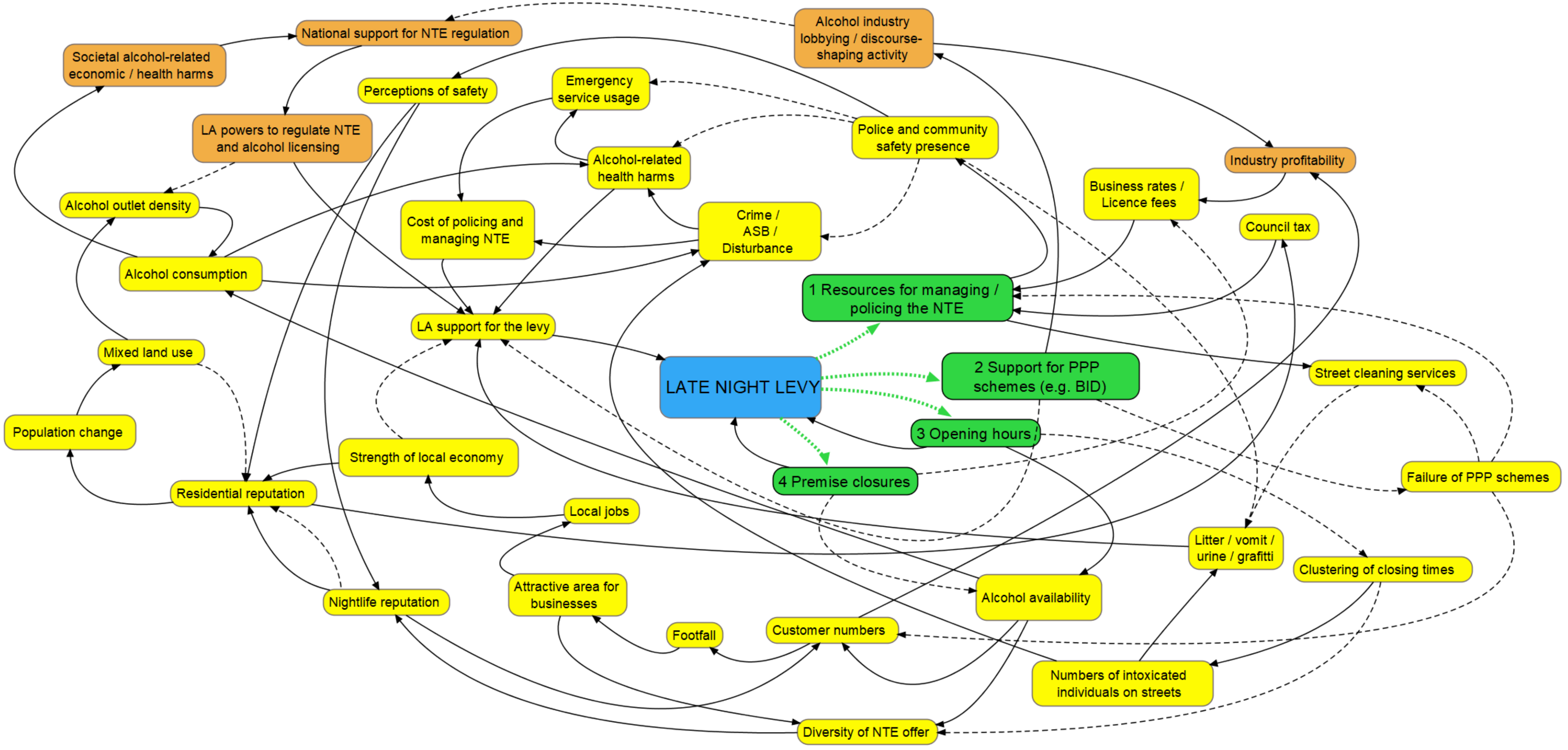

Orange bubbles = national variables; Yellow bubbles = local variables; Green bubbles = immediate theorised impacts stemming from levy introduction
Solid line: positive relationship between variables; Dashed line: inverse relationship between variables; Dotted green line: theorised impacts stemming from the levy introduction
